# Site‐Specific Polymer‐Protein‐Polymer Conjugates for the Preparation of Dual Responsive Multilayer Nanoparticles

**DOI:** 10.1002/smll.202500531

**Published:** 2025-03-04

**Authors:** Melina I. Feldhof, Simon Walber, Sandro Sperzel, Susanne Boye, Ulla I.M. Gerling‐Driessen, Laura Hartmann

**Affiliations:** ^1^ Department of Organic and Macromolecular Chemistry Heinrich‐Heine‐University Düsseldorf Universitätsstraße 1 40225 Düsseldorf Germany; ^2^ Institute for Macromolecular Chemistry University of Freiburg Stefan‐Meier‐Str. 31 D‐79104 Freiburg i.Br. Germany; ^3^ Advanced Macromolecular Structure Analysis Leibniz‐Institut für Polymerforschung Dresden Hohe Str. 6 01069 Dresden Germany

**Keywords:** dual responsive multilayer nanoparticles, ligand‐receptor interaction, polymer‐protein‐polymer conjugates, rebridging agent, thiol‐induced light activated controlled radical polymerization (TIRP)

## Abstract

Protein‐polymer‐based materials demonstrate high potential in advanced applications. However, controlled combinations of multiple proteins and polymers to obtain multimaterial systems is limited due to the complexity of retaining protein structure and function and achieving high structural control for the polymers simultaneously. Here, the first combination of a rebridging agent and thiol‐induced, light‐activated controlled radical polymerization (TIRP) is introduced to directly enable site‐specific conjugation of two different polymers to native proteins. Specifically, poly(*N*‐isopropyacrylamide) (pNIPAM) is attached to bovine serum albumin (BSA), followed by incorporation of a new rebridging agent, and initiating a second TIRP to introduce a glycopolymer, giving highly defined pNIPAM‐BSA‐glycopolymer conjugates. Above the lower critical solution temperature (LCST), nanoparticles with a glycopolymer corona are formed. The addition of a glycan‐specific lectin leads to the formation of a second protein corona and so‐called multilayer nanoparticles. Depending on the sequence of stimuli, the particles can either undergo a step‐wise or one‐step disassembly. Furthermore, by controlling the ratio of binding/non‐binding glycopolymers in the multilayer nanoparticles, either distinct nanoparticles or large clusters can be formed. Thus, dual‐responsive multilayered polymer‐protein nanoparticles are now accessible with controlled and programmable material properties such as assembly and disassembly while maintaining the protein's native structure and thus function.

## Graphical abstract

Dual‐responsive multilayer nanoparticles are obtained from combining a new rebridging agent and thiol‐induced, light‐activated controlled radical polymerization giving access to site‐selective polymer‐protein‐polymer conjugates that enable dual‐responsive nanoparticle self‐assembly and disassembly.

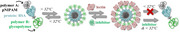

## 1 Introduction

Recently, proteins have gained increasing interest for the use in material applications as biobased alternative to fuel‐based polymeric materials.^[^ ^1^ ^]^ In order to optimize properties and obtain materials with specific functions, proteins are either enhanced, e.g., through biological engineering or through chemical modifications to optimize properties beyond the original protein itself.^[^ ^2^ ^]^ Protein‐polymer conjugates are a well‐established class of such modified proteins and are widely recognized for their relevance in various applications, from medical applications of protein drugs to the use of enzymes in biocatalysis.^[^ ^3^ ^]^ Proteins alone would often be insufficiently stable, e.g., for prolonged circulation times in vivo or under non‐aqueous conditions for catalysis;^[^ ^3^, ^4^ ^]^ whilst polymers alone do not provide the advanced functionality of proteins. It is the combination of both, polymer and protein, that enables the desired properties and functions.^[^ ^3^, ^5^ ^]^ However, in order to achieve such synergism, proteins and polymers have to be linked in a way that does not affect the protein structure and allows high control over the polymer, e.g., in terms of dispersity, chain length and site‐selective attachment to the protein.^[^ ^3b^ ^]^ Controlled design of protein‐polymer conjugates then allows to derive materials with advanced properties as is demonstrated in this study for the preparation of multilayer protein‐polymer nanoparticles from the stimuli‐responsive self‐assembly of highly controlled polymer‐protein conjugates.

Nanoparticles are defined as colloidal structures in nanometer range (1–1000 nm in diameter) that can be generated, e.g., through emulsification, thermal structuring, hydrophobic interactions, and nanoprecipitation.^[^ ^4^, ^6^ ^]^ Nanoparticles composed exclusively of proteins exhibit inherent biocompatibility and biodegradability, which reduces the potential for toxicity and adverse immune responses.^[^ ^6^, ^7^ ^]^ However, these nanoparticles exhibit notable stability limitations, which can be enhanced by incorporating polymers.^[^ ^7^ ^]^ Site‐selective polymer‐protein conjugation with, e.g., thermal‐ or pH‐responsive polymers are well known for the stimuli‐responsive self‐assembly of protein‐based nanoparticles.^[^ ^8^ ^]^ For instance, poly(*N*‐isopropyl acrylamide) (pNIPAM) conjugated proteins self‐assemble into nanoparticles below their lower critical solution temperature (LCST),^[^ ^9^ ^]^ while poly(2‐(diisopropylamino)ethyl methacrylate) conjugated proteins form pH‐responsive nanoparticles,^[^ ^8b^ ^]^ both featuring a hydrophobic core and a hydrophilic protein shell. Although polymer‐protein conjugates demonstrate significantly improved stability, they also exhibit limitations in terms of potential loss of protein functionality and complexity of synthesis.^[^ ^10^ ^]^

Typically, the synthesis of protein‐polymer conjugates involves controlled polymerization methods, such as atom transfer radical polymerization (ATRP)^[^ ^3^, ^11^ ^]^ or reversible addition−fragmentation chain‐transfer (RAFT)^[^ ^3^, ^12^ ^]^ performing either a grafting‐to or grafting‐from synthesis.^[^ ^13^ ^]^ However, challenges in controlled polymerizations include potential protein denaturation and inactivation due to chemical conditions such as organic solvents or high temperatures.^[^ ^14^ ^]^ More recent advances introduced the use of aqueous^[^ ^3^, ^15^ ^]^ or light‐activated polymerizations^[^ ^16^ ^]^ and particularly the combination of both, recently introduced by Haddleton^[^ ^15b^ ^]^ and Velonia and Anastasaki,^[^ ^3f^ ^]^ which minimizes potential damage to the protein structure and thus preserves bioactivity. In our own work, we have recently introduced a new synthetic methodology to give straightforward access to site‐specific conjugation of polymers to native proteins and enzymes without any prior protein modifications and maintaining the protein's native structure, stability and activity by using thiol‐induced, light‐activated controlled radical polymerization (TIRP).^[^ ^17^ ^]^

**Figure 1:**
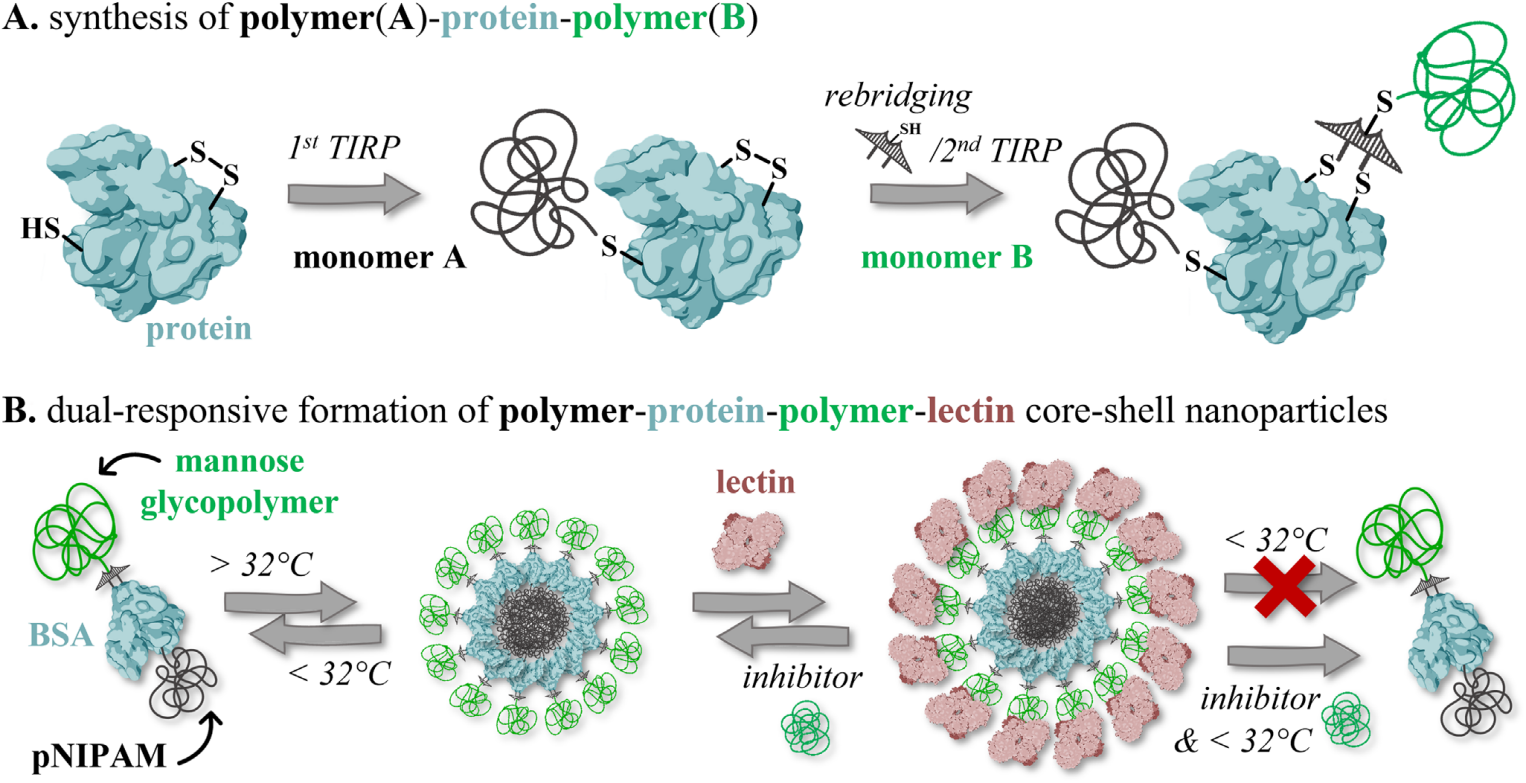
A) Schematic representation of the synthesis of polymer(pNIPAM)‐protein‐polymer via 1^st^ TRIP followed by first 2^nd^ TIRP introduced by the rebridging agent. Β) Schematic representation of dual responsive formation of multilayer (core‐shell) nanoparticles. Potentially, particles have a separated or mixed BSA‐pNIPAM core above the LCST. In this schematic drawing this is, however, depicted as a core‐shell structure only for simplicity reasons. At this time, we have no experimental evidence to proof which type of core (core‐shell or mixed) is formed. This type of schematic drawing is used throughout the manuscript.

However, so far, this method only allowed for polymer conjugation on native proteins presenting a free thiol side chain and introducing the same polymer at all available positions. Here, we now present a new strategy that gives straightforward access to highly controlled, multifunctional protein‐polymer conjugates presenting site‐selectively two different polymers on one protein. To do so, we combine TIRP and so‐called rebridging agents,^[^ ^18^ ^]^ now allowing also disulfide groups in the protein structure to be accessed for TIRP. Rebridging agents, e.g., based on bissulfones,^[^ ^19^ ^]^ disubstituted maleimides like bromomaleimides,^[^ ^18c^ ^]^ arsenous acids,^[^ ^20^ ^]^ dibromopyridazinediones,^[^ ^21^ ^]^ alkynes,^[^ ^22^ ^]^ allyl sulfones,^[^ ^23^ ^]^ or diethynyl phosphonites^[^ ^24^ ^]^ are well‐established for introduction of functional handles into the disulfide bond of native proteins thereby retaining the protein structure and function.^[^ ^25^ ^]^ For this study, we developed a new rebridging agent, based on a symmetrical dibromomaleimides, allowing us to introduce an additional free thiol residue without compromising the original disulfide bond. The symmetrical design ensures that after intramolecular disulfide reduction of the rebridging agent, both fragments are chemically identical, even if potentially the rebridging agent would react by bridging two proteins in the first insertion step. In short, the protein – here bovine serum albumin (BSA) is first site‐selectively functionalized with one type of polymer at the free cysteine residue (Cys’34) using TIRP.^[^ ^17^ ^]^ Next, exactly one of the intramolecular disulfide bridges of the protein is opened by reduction and the rebridging agent is introduced. However, at this time, we do not know which one of the disulfide bonds is accessed as this would require more complex protein analysis. This newly introduced thiol is then used in a second TIRP introducing a second type of polymer. Thus, to the best of our knowledge, we present for the first time the preparation of polymer‐protein‐polymer conjugates where two different polymers are site‐selectively, covalently attached to a protein. We demonstrate the variability of this approach by introducing two different functional polymers at two selected positions in the protein structure – pNIPAM for temperature‐induced nanoparticle formation and glycopolymers for ligand‐induced formation of a lectin corona – and how this enables the formation of dual responsive multilayer nanoparticles (**Figure** **1**). We can also demonstrate, that the lectin corona protects the nanoparticles against temperature‐induced disassembly and only in the presence of high affinity competing ligands, nanoparticles disassemble back into single polymer‐protein conjugates. This dual responsive formation of multilayer nanoparticles opens new opportunities in the development of functional, protein‐based materials, e.g., for biomedical applications such as drug delivery, or the use of enzymes as advanced biocatalysts.

## 2 Results and Discussion

### 2.1 Synthesis of Protein‐Polymer and Polymer‐Protein‐Polymer Conjugates

**Figure 2:**
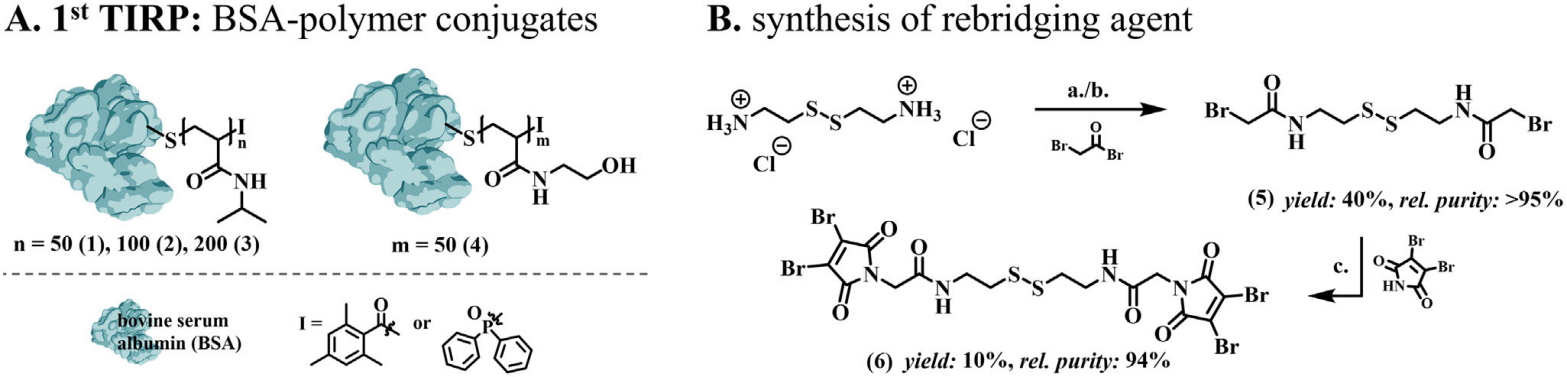
A) Presentation of the TIRP‐based synthesized BSA‐pNIPAM conjugates (**1**–**3**) and BSA‐pHEAA conjugate (**4**). B) Synthesis of the rebridging agent (**6**) for conjugation to BSA‐polymer precursor **1** and **4** to produce defined bifunctionalized, protein‐polymer conjugates. The yields and relative purities are presented as percentages. The relative purity was determined using LC‐MS. Reaction conditions: **a**. NaOH, H_2_O, 15 min, RT, **b**. CDCl_3_, 2 h, RT, **c**. K_2_CO_3_, THF, N_2_, 2 h, RT.

As protein for polymer functionalization, we chose well‐studied BSA as it is extensively used for various applications.^[^ ^26^ ^]^ We first synthesized BSA‐pNIPAM conjugates with different polymer length (**1**–**3**) and BSA‐p(*N*‐hydroxyethyl acrylamide) (BSA‐pHEAA) (**4**) using TIRP on native BSA, see **Figure** **2**A.^[^ ^17^ ^]^ Protein‐polymer conjugates were analyzed by ^1^H‐NMR spectroscopy, asymmetric flow field flow fractionation with light scattering detection (AF4‐LS), circular dichroism (CD), sodium dodecyl sulfate‐polyacrylamide gel electrophoresis (SDS‐PAGE), infrared (IR) spectroscopy and dynamic light scattering (DLS) (see Supporting Information). As previously demonstrated,^[^ ^17^ ^]^ also for these conjugates polymer conjugation does not affect the protein structure and stability. To introduce a second, different polymer chain at another position in the protein, we developed a new type of rebridging agent that allows us to introduce another free thiol into the protein. We chose the rebridging process, as this has been shown to preserve the 3D structure of the protein and thereby also the protein's function.^[^ ^18a^ ^]^ Rebridging agents, e.g., based on bissulfones,^[^ ^19^ ^]^ disubstituted maleimides like bromomaleimides,^[^ ^18c^ ^]^ arsenous acids,^[^ ^20^ ^]^ dibromopyridazinediones,^[^ ^21^ ^]^ alkynes,^[^ ^22^ ^]^ or allyl sulfones^[^ ^23^ ^]^ are well‐established for introduction into a disulfide bond of native proteins thereby retaining the protein structure and function but also introducing an orthogonal functional group for selective conjugation to the protein.^[^ ^25^ ^]^ Here we developed a new rebridging agent allowing us to introduce an additional free thiol residue without compromising the original disulfide bond. Notably, disulfide bonds are integral to the structural integrity and 3D conformation of proteins, thus a simple reductive opening of the disulfide to achieve a free thiol would likely result in a destabilization of the protein structure and thus loss of function.

The new rebridging agent was obtained by reacting cystamine with bromoacetyl bromide in aqueous sodium hydroxide solution to obtain intermediate structure **5** and subsequent reaction with 2,3‐dibromomaleimide to final rebridging agent **6** (Figure 2B, see Supporting Information for analytical data and further information on the synthesis). The symmetrical design of the rebridging agent is intended to prevent the formation of two products during conjugation, thereby enabling the uniform modification of the proteins. Dibromomaleimide was chosen as the reactive group as it forms irreversible linkages, making retro‐Michael deconjugation highly unlikely and persists even after hydrolysis, ensuring the stability of the rebridged protein during further application and analysis.^[^ ^27^ ^]^

Introduction of the rebridging agent into the BSA‐pNIPAM conjugate **1** and BSA‐pHEAA conjugate **4** was achieved following a protocol by Heredia et al.^[^ ^28^ ^]^ which allows for the reduction of only one of the native disulfide bonds of BSA by using 10 equivalents of tris(2‐carboxyethyl)phosphine (TCEP) as reducing reagent, as is also confirmed by an Ellman's Assay (see Supporting Information for further information). After removal of the reducing agent, the rebridging agent (**6**) was added. Successful conjugation was monitored by ultraviolet–visible (UV/Vis) spectrophotometry and fluorescence measurements (Supporting Information), as the formed rebridged protein is UV active with an intrinsic fluorescence, as exemplified by O'Reilly et al.^[^ ^29^ ^]^ Next, to release the free thiol required for TIRP, 10 eq. TCEP as reducing agent was added again, and removed again through dialysis, the second polymerization was performed using TIRP at previously developed reaction conditions. As monomers, acrylate‐modified mannose (**S1**), and acrylate‐modified galactose (**S2**) was used to modify the BSA‐pNIPAM precursor (**1**) and NIPAM were used to modify the BSA‐pHEAA (**4**) precursor. After polymerization, all protein‐polymer (**7**–**9**) conjugates were purified via dialysis (**Table** **1**).

**Table 1:** Presentation of the defined biofunctionalized BSA‐polymer conjugates synthesized via rebridging in combination with TIRP: non‐functional system (**7**) and two glycopolymer conjugates bases a mannose (**8**) and galactose (**9**). Visualization of potential hydrolysis of the conjugated rebridging agent.^[^ ^27b^ ^]^

Sample Labeling	Structure	1^st^ Polymer	2^nd^ Polymer
pHEAA‐BSA‐pNIPAM (**7**)	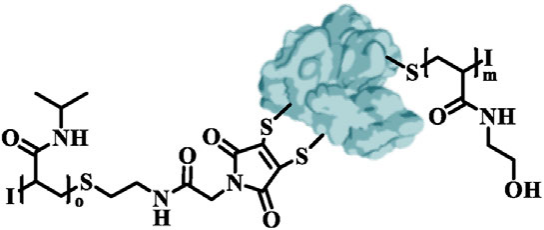	pHEAA	pNIPAM
pNIPAM‐BSA‐pMan (**8**)	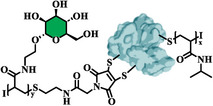	pNIPAM	pMan
pNIPAM‐BSA‐pGal (**9**)	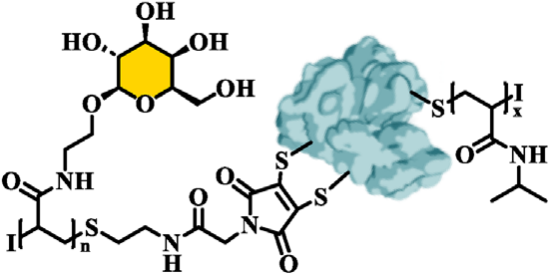	pNIPAM	pGal

**Figure 3:**
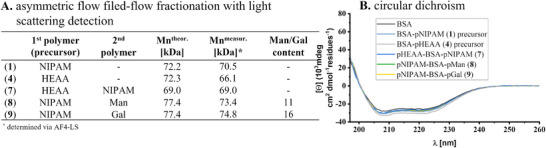
A) Analytical data for molar masses of monomeric BSA population of protein–polymer conjugates **1**, **4**, **7**–**9** via AF4‐LS in PBS buffer (pH 7.4 at 25 °C). The theoretical molecular weights were determined based on the measured AF‐4 data of the precursors, taking into account the intended polymer length. B) Presentation of CD spectra of BSA, BSA–polymer precursor **1**, **4**, and bifunctionalized rebridged structures **7**–**9** for investigation of structural properties. The measurements were performed in PBS buffer (pH 7.4, at 20 °C) with an exact concentration of 251 µg mL^−1^ adjusted by the Pierce‐Protein‐Assay.

Successful polymerization and formation of polymer‐protein‐polymer conjugates were confirmed by proton nuclear magnetic resonance (^1^H‐NMR), AF4‐LS, and CD spectroscopy (see Supporting Information for further information). AF4‐LS^[^ ^30^ ^]^ was found to be effective in gently separating the different BSA populations (monomeric BSA, BSA dimer, and trimer/multimer), and corresponding BSA–polymer conjugate.^[^ ^17^ ^]^ By analyzing the structures presented here, the analysis simplifies its approach by concentrating on the molar masses of the monomeric population while similar trends are observed in the dimer fractions (see Supporting Information for further information). AF4‐LS measurements confirmed that the protein–polymer conjugates (**7**–**9)** with two different polymers obtained higher molar masses in comparison to the initial BSA‐polymer conjugate (**1** or **4)** **Figure** **3**A.

We confirmed by CD measurements that also after rebridging and second polymerization the conformation of the protein was retained, see Figure 3B (see also Supporting Information).

### 2.2 Nanoparticle Formation from Defined Polymer‐Protein‐Polymer Conjugates

Based on the different types of polymers we introduced into to protein‐polymer and polymer‐protein‐polymer conjugates, we can now use two different stimuli to induce the formation of aggregates. On the one hand, temperature induced aggregation from the LCST behavior of the pNIPAM chains is possible. On the other hand, recognition and binding of glycopolymers by a lectin (carbohydrate‐recognizing protein) leads to the formation of glycopolymer‐protein complexes.^[^ ^17^ ^]^

First, we studied the temperature dependent cluster formation of the BSA‐pNIPAM precursors **1**–**3** with varying chain length of the pNIPAM (50, 100, or 200 repeating units). The temperature‐induced aggregation and nanoparticle formation was investigated using DLS in phosphate buffered system (pH 7.4). As expected, all protein‐polymer conjugates show higher hydrodynamic diameter (D_h_) than the native BSA. We observe no aggregation based on LCST for native BSA or BSA‐pHEAA (**4**) (further information in SI). For all BSA‐pNIPAM conjugates, temperature‐dependent cluster formation is clearly observed. The formed clusters have very low dispersities (PdI = 0.03–0.25) and can be completely restored to their initial non‐aggregated state by lowering the temperature again below the LCST (**Table** **2**).

**Table 2:** Summary of all data determined via DLS of the synthesized conjugates **1**–**4** (c = 0.5 mg mL^−1^) and native BSA. The corresponding DLS runs are shown in the Supporting Information.

Structure	Mn [kDa]^a^ ^)^	< LCST [20 °C]		temp.Ramp [min]	> LCST [44 °C]		LCST^d^ ^)^ [°C]
		D_h_[nm]^b^ ^)^	PdI^b^ ^)^		D_h_[nm]^c)^	PdI^c)^	
(**1**)	50	10.6 ± 0.3	0.35 ± 0.02	2	130 ± 0.3	0.10 ± 0.01	35.5
				10	136 ± 0.9	0.14 ± 0.01	35.2
				20	189 ± 0.4	0.19 ± 0.01	33.6
(**2**)	100	16.1 ± 0.39	0.56 ± 0.01	2	130 ± 0.7	0.05 ± 0.02	35.0
				10	165 ± 0.9	0.04 ± 0.02	33.6
				20	363 ± 0.2	0.13 ± 0.02	33.3
(**3**)	200	17.2 ± 0.61	0.48 ± 0.01	0	156 ± 0.1	0.04 ± 0.01	35.8
				2	158 ± 0.9	0.06 ± 0.01	34.9
				10	186 ± 0.8	0.11 ± 0.01	33.7
				20	260 ± 0.1	0.16 ± 0.01	32.4
(**4**)	50	10.7 ± 0.19	0.35 ± 0.01	2	14.8 ± 1.	0.23 ± 0.15	−
				10	12.9 ± 1.8	0.23 ± 0.06	−
				20	13.1 ± 0.1	0.20± 0.07	−
BSA native	66.5	8.2 ± 0.32	0.17 ± 0.04	2	8.44 ± 0.1	0.23 ± 0.01	−
				10	9.34 ± 0.2	0.26 ± 0.02	−
				20	9.30 ± 0.0	0.26 ± 0.00	−

^a)^ determined via AF4/SEC measurements;

^b)^ determined via DLS ninefold measurements;

^c)^ determined via DLS triplicate measurements;

^d)^ LCST calculation via Hill 1 Fit.

**Figure 4:**
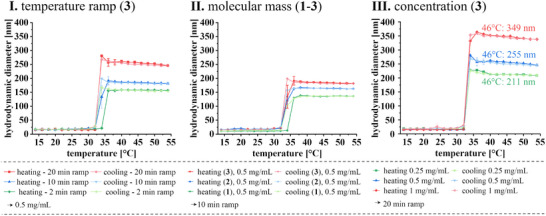
Comparison of the DLS measurement results with respect to the **I**: molar mass (**1**–**3**), **II**: temperature ramp effect (1–**3**), **III**: concentration (**3**) of the conjugates: BSA‐pNIPAM (**1**), BSA‐pNIPAM (**2**), BSA‐pNIPAM (**3**) in PBS buffer (pH 7.4).

LCST of the conjugates is in the range of 32.4–35.8 °C and thus close to the LCST of free pNIPAM chains^[^ ^31^ ^]^ and comparable protein‐pNIPAM conjugate systems in the literature.^[^ ^32^ ^]^ Size of the nanoparticles can be tuned in the range from 130 to 363 nm by varying the molar mass of the pNIPAM, the heating ramp, and the weight concentration of the conjugates in solution (see **Figure** **4**).

Next, we investigated the temperature‐induced clustering of the polymer‐protein‐polymer conjugates (**8**,**9**). The revealed hydrodynamic diameters for pMan‐BSA‐pNIPAM conjugate **8** and pGal‐BSA‐pNIPAM conjugate **9** show hydrodynamic diameters below LCST (20 °C) of 62 and 71 nm, respectively. This substantial increase in hydrodynamic diameter in comparison to the BSA‐pNIPAM precursor with 10.6 nm is attributed to the hydrate shell of the glycopolymer and supports the successful formation of a polymer‐protein‐polymer conjugate. Furthermore, conjugates **8** and **9** exhibit lower critical solution temperatures at 34.3 °C and 34.8 °C, respectively, compared to BSA‐pNIPAM **1** at 35.5 °C. Studies on other materials combining pNIPAM and carbohydrates have shown that increased hydrophilicity by incorporating carbohydrates in pNIPAM conjugates can lead to a decrease in the LCST, which is potentially attributed to faster elimination of the pNIPAM hydration layer due to the H‐bonds of the carbohydrates.^[^ ^33^ ^]^ We observe this effect in our systems, attributed to the presence of hydrophilic glycopolymers in the protein‐polymer‐protein conjugates. We hypothesize that in the polymer‐protein‐polymer cluster above LCST, the nanoparticles have a glycopolymer corona while the pNIPAM and BSA form the core of the clusters – potentially also as core‐shell or a mixed core structure. Presentation of the glycopolymers on the outside of the clusters should thus also allow us to add a second protein – a carbohydrate recognizing lectin – that can now bind to the nanoparticles forming another layer or shell.

To study this, we utilized the model lectin Concanavalin A (Con A) which is well characterized for its specific binding to mannose ligands such as in the Man‐presenting glycopolymers of conjugate **8**. We start with the pMan‐BSA‐pNIPAM (**8**) conjugate below the LCST at 28 °C (LBB buffer, pH 7.4) showing an initial hydrodynamic diameter of 69 nm. This size can likely not only be attributed to carbohydrate‐induced hydration but may also be affected by the formation of pre‐assembled structures driven by hydrogen bonding. Above the LCST (38 °C, 2 min ramp) nanoparticles with hydrodynamic diameters of 196 nm are formed, presenting glycopolymer chains with an average of 11 mannose side chains per polymer chain (degree of polymerization). Then we added ConA in LBB buffer pH 7.4 and observed a further increase in size of the nanoparticle to now 52 nm. ConA alone has a hydrodynamic diameter of ≈8.8 nm,^[^ ^34^ ^]^ thus in the formed nanoparticles roughly three ConA molecules have attached in the formation of the lectin corona in height.

This Man to ConA ratio aligns with previous studies from Haddleton et al.^[^ ^35^ ^]^ examining the number of ConA (7 units) binding sites on mannose‐based glycopolymers (23 mannose units) with an acrylate backbone under identical measurement conditions. Consequently, the data obtained for the three ConA units on the corona indicates a near‐maximum loading of the cluster. The formation of nanoparticles was further validated by fluorescence microscopy using fluorescently labeled ConA (Figure 6B). When adding ConA to conjugate **8** below the LCST (28 °C) and only then increasing the temperature above the LCST, similarly sized nanoparticles are formed. This nicely demonstrates that nanoparticle formation is controlled by the design of the polymer‐protein‐polymer conjugates rather than cluster formation conditions. To confirm that formation of conjugate‐lectin clusters is based on specific interactions, inhibition experiments through addition of free pMan were performed. A decrease in size back to D_h_ of 191 nm was observed, which corresponds to the size of the cluster without ConA above LCST and thus confirms the complete removal of the lectin from the cluster. Specificity is also demonstrated from the negative control experiment using pGal‐BSA‐pNIPAM conjugate (**9**) as ConA does not bind to galactose. Indeed, here no increase in nanoparticle size is observed upon addition of ConA. Overall, we can show that through the conjugation of two distinct polymers to BSA, we can achieve dual‐responsive aggregation behavior and form‐controlled polymer‐protein nanoparticles.

**Figure 5:**
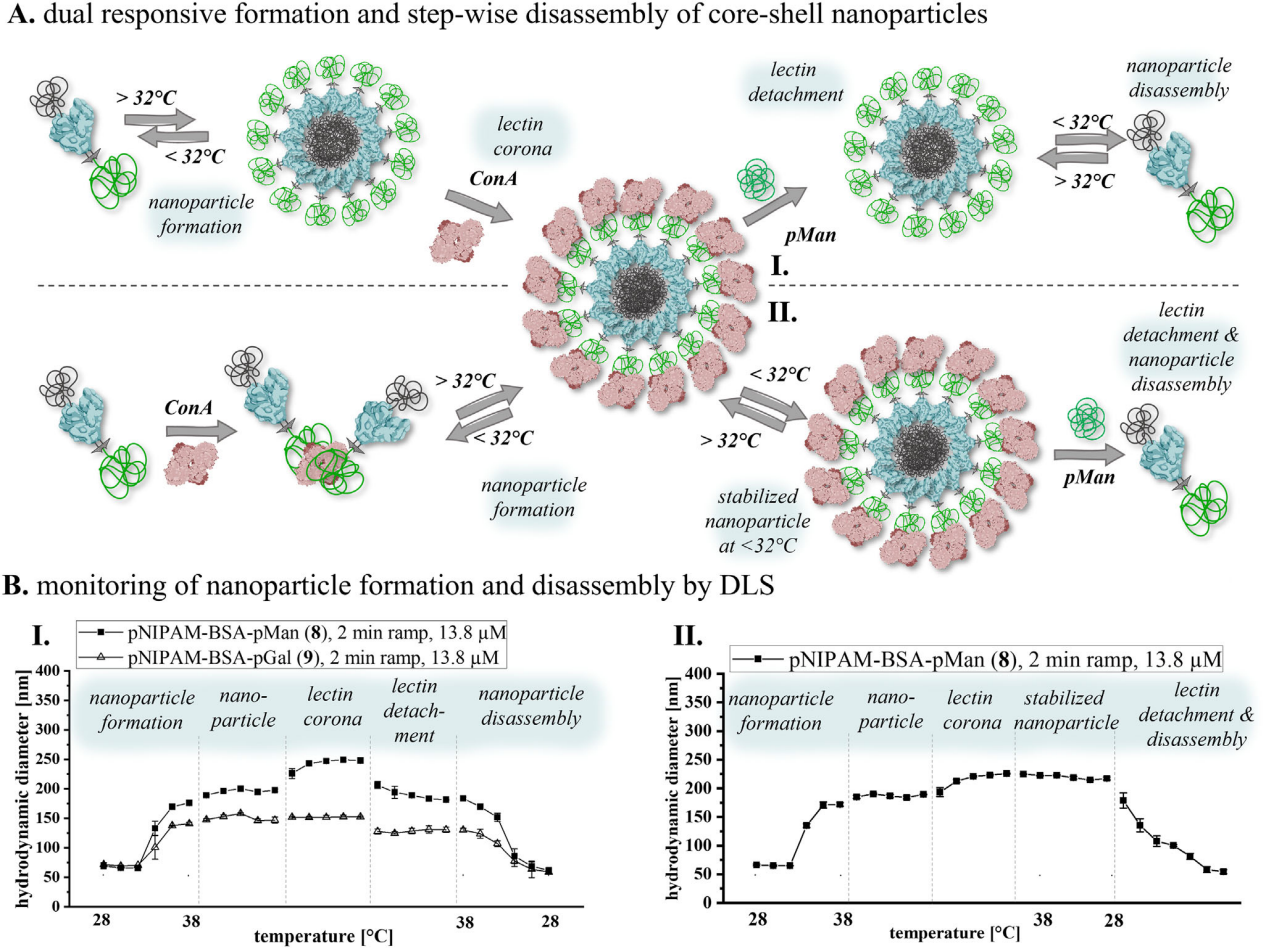
A) Schematic demonstration of two ways for thermally switchable core‐multi‐shell hybrid nanoparticle formation with presentation of hydrophilic carbohydrate polymers on the surface for specific binding to the lectin ConA. In addition, the schematic disassembly of the lectin corona above the LCST followed by the thermal disassembly of the cluster or the nanoparticle stabilized by the lectin below the LCST. B) **I**. Comparison of DLS measurements of mannose‐ and galactose‐based bivalent systems. Formation of the defined cluster owing to the thermosensitive polymer conjugate. Thereupon, the formation of a specific interaction with ConA, exclusively the mannose‐based cluster, and the associated increasing hydrodynamic diameter. The galactose‐based cluster did not interact with lectin. Thereafter, detachment with the pMan polymer (**S3**) and decreasing D_h_ before the defined cluster is dissolved again by lowering the temperature. **II**. The difference from set‐up I is that after the addition of ConA, the temperature is first lowered (< LCST) and the cluster can thus be fixed. Only then is the inhibitor added, and the entire cluster is dissolved.

**Figure 6:**
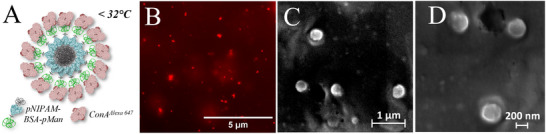
A) Schematic demonstration of the investigated state over fluorescence microscopy and SEM. B) Fluorescence image of the pNIPAM‐BSA‐pMan (**8**) below the LCST with addition of the lecin ConA^Alexa 647^ Fluorescence image (excitation: 641 nm, emission: 690 nm). C./D. SEM images of pNIPAM‐BSA‐pMan (**8**) fixed via ConA as nanoparticles in LBB below the LCST with concentrations at 0.46 µm.

Finally, we investigated whether – similar to the stepwise formation of the nanoparticles from the two distinct assembly stimuli – we can also disassemble the nanoparticles in a controlled manner. Such reversible, stimuli‐responsive aggregation and nanoparticle formation has great relevance for the potential future applications of these materials, e.g., for drug encapsulation and release. As control experiment, we already showed that addition of excess of free pMan leads to selective disassembly of the lectin corona. As expected, when reducing the temperature below the LCST, the nanoparticle disassembles completely back into the non‐aggregated polymer‐protein‐polymer conjugates. Interestingly, when starting again from the multi‐shell nanoparticles with lectin corona and first reducing the temperature below the LCST, no change in the hydrodynamic diameter of the nanoparticles is observed (**Figure** **5**B**II**). Thus, the lectin corona stabilizes the clusters against disassembly. Stabilized nanoparticles can also be visualized and confirmed by fluorescence microscopy using fluorescently labeled Alexa^647^‐ConA as well as by SEM (see **Figure** **6**C,D), with the nanoparticle being stable for several days (see Supporting Information).

Only when we add pMan as inhibitor, do we observe disassembly, now immediately into the free polymer‐protein‐polymer conjugates. Thus, depending on the sequence of the applied stimuli (temperature, glycan inhibitor) we can control the disassembling process either in a stepwise fashion or in a spontaneous complete disassembly back into the free conjugates.

To confirm this finding, we also performed microscopy studies using fluorescently labeled Alexa^647^‐ConA and scanning electron microscope (SEM) images, see Figure 6.

### 2.3 Binding and Non‐Binding Carbohydrate Mixed Multi‐Shell Hybrid Nanoparticle

**Figure 7:**
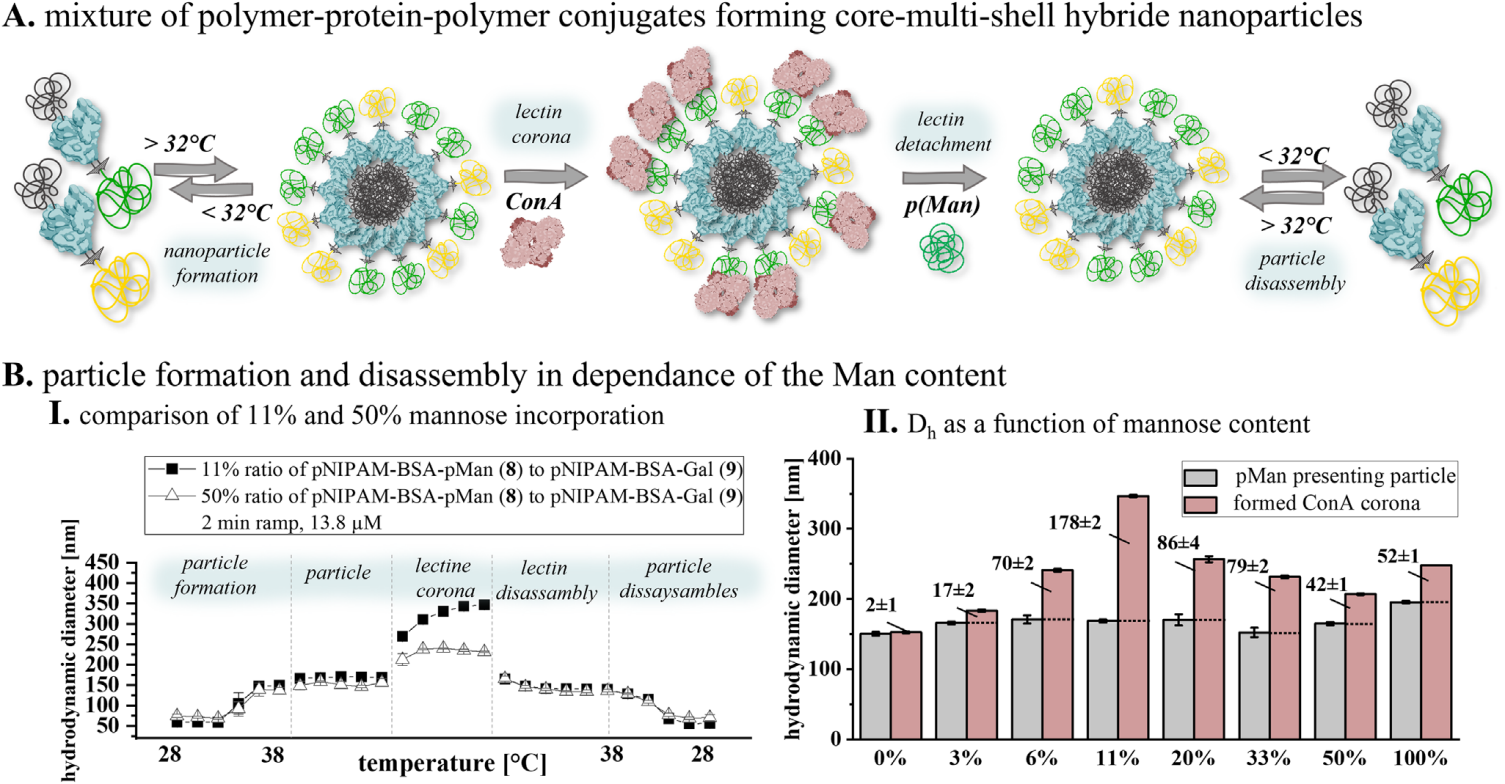
A) Schematic demonstration of core‐multi‐shell hybrid nanoparticle formation out of a mixture of mannose (**8** – binding ligand) or galactose (**9** – non‐binding ligand) bifunctionalized protein‐polymer conjugates. B) **I**. Comparison of DLS curves between 11% and 50% mannose (**8**) ratio in comparison to galactose (**9**) (LBB buffer, 13.8 µm, 2 min ramp). **II**. Plot of hydrodynamic diameter bevor and after ConA adding and the corresponding increase in D_h_ at different mannose to galactose ratios. All experimental data are available in detail in the SI, including cluster formation and inhibition of lectin.

This study has demonstrated the capability to generate and stimuli‐control highly defined multi‐shell nanoparticles from polymer‐protein‐polymer conjugates made accessible through TIRP in combination with a new rebridging agent. Specifically, we used pNIPAM to induce temperature‐dependent nanoparticle formation, followed by using the glycopolymers in the corona of the nanoparticle presenting either mannose‐ or galactose to specifically bind lectins and thereby add a second protein shell to the nanoparticles. Since both types of conjugates, mannose‐ or galactose‐presenting nanoparticles, are derived from the same BSA‐pNIPAM precursor, it is now also feasible to combine these two conjugates while maintaining controlled nanoparticle formation. One of the key binding mechanisms of carbohydrate‐lectin interactions is multivalent binding. Single carbohydrate interactions are typically weak (in the µm to mm) range.^[^ ^36^ ^]^ Nature uses the multivalent presentation of such low affinity carbohydrates, e.g., in glycoprotein conjugates to ensure high avidity and high selectivity binding.^[^ ^36^, ^37^ ^]^ However, an increase in binding through multivalent interactions is not achieved through a simple “the more the better” principle. Rather it is well understood, that the combination of binding and non‐binding carbohydrates in both, natural and mimetic glycoconjugates plays a key role in maximizing multivalent binding.^[^ ^38^ ^]^ From mixing the two polymer‐protein‐polymer conjugates with mannose or galactose‐functionalization, respectively, we can now easily vary the ratio of the two carbohydrates in the resulting nanoparticle and study the effect on lectin binding, **Figure** **7**A.

Different ratios of conjugates **8** and **9** were mixed in solution (0, 3, 6, 11, 20, 33, 50, or 100% of mannose‐conjugates mixed with the corresponding percentage of the galactose‐conjugates) and then treated under the same aggregation conditions as previously established for the single conjugate systems (first increasing the temperature above the LCST, followed by addition of ConA). For samples containing no (0%) or only 3% of mannose‐conjugate, we observe nanoparticles that correspond in size to the clusters prior to ConA addition, with a slight increase for the 3% samples (Figure 7BII). The increase in hydrodynamic diameter of nanoparticles formed at 50% quite are similar to those formed from all mannose conjugates (100%). Interestingly, for nanoparticles formed with 6–33% mannose‐conjugates we observe increasing hydrodynamic diameters with increasing mannose‐conjugate contents. This can be explained by more ConA binding to the polymer‐protein‐polymer nanoparticles which is in line with previous observations where lower density of binding carbohydrates, e.g., among non‐binding epitopes in glycopolymers, has enabled more lectin binding.^[^ ^38^, ^39^ ^]^ Notably, the sample with 6% mannose incorporation exhibits no reduction in hydrodynamic diameter, suggesting that the ConA content remains unchanged. This indicates that a minimal quantity of binding ligand may be adequate to attract the maximum amount of lectin. The 11% sample shows an increase of noticeable 178 nm after ConA addition, which suggests that factors beyond the increased ConA content, such as the limited availability of mannose ligands, may also be playing a role. We hypothesize that ConA with its four binding sites bridges mannose ligands from two different nanoparticles, which is supported by our hydrodynamic diameter measurements. DLS measurements over various mannose ratios exhibit a predominantly monomodal distribution before and after lectin addition, suggesting well‐defined, uniform nanoparticles. Only the 11% sample displays a broad, multimodal distribution by lectin corona building, indicating diverse conformations and supporting the hypothesis of lectin‐bridged nanoparticles (see Supporting Information). To proof that cluster formation is still induced by specific carbohydrate‐lectin interactions, inhibition experiment by adding mannose‐presenting free glycopolymer were performed again and show full disassembly of cluster aggregates for all systems. We also observed that the kinetics of disassembly are different for different mannose‐to‐galactose ratios in the clusters, however, we will need more advanced time‐resolved measurements to study this further.

## 3 Conclusion

We introduce the site‐specific conjugation of two different polymers – pNIPAM and glycopolymer – to native proteins by combining a novel rebridging agent and TIRP giving access to dual‐responsive multilayer nanoparticles with stimuli‐triggered self‐assembly and disassembly. First, temperature‐induced aggregation of pNIPAM leads to the formation of nanoparticles that are stabilized by a glycopolymer corona. A second protein corona can then be added by leveraging specific glycopolymer‐lectin interactions. This outer particle corona of a non‐covalently bound lectin efficiently protects the nanoparticles from temperature‐induced disassembly. Thus, depending on the sequence of stimuli, the particles can be disassembled in a step‐wise or one‐step fashion. Furthermore, from mixing polymer‐protein conjugates presenting different types of glycopolymers – lectin binding and non‐binding – at defined ratios, the formation of defined multilayer nanoparticles or large nanoparticle clusters can be controlled. This novel approach expands the functionality of protein‐based materials, which can now also be transferred to enzymes in future research, opening new possibilities for applications, e.g., in drug delivery or biocatalysis.

## 4 Experimental Section

### Standard Protocol for Introducing the Rebridging Agent on Previous Synthesized BSA‐Polymer Conjugates and Reduction of the Disulfide Bridge from the Rebridging Agent

40 mg (0.6 µmol) of BSA‐p(HEAA) (**4**) or BSA‐pNIPAM (**1**) conjugate was dissolved in 1.5 mL PBS buffer. To this, 100 µL of a 60 mm (10 eq.) tris(2‐carboxyethyl)phosphine (TCEP) stock solution in PBS buffer was added and stirred for 10 min at room temperature. The solution was briefly purified via dialysis (exclusion volume 50 kDa, refilled to 1.5 mL with PBS), followed by a second addition of 100 µL of TCEP stock solution (60 mm in PBS) and further stirring for 10 min at room temperature. The remaining TCEP residues were completely removed by repeated purification with deoxygenated PBS buffer via dialysis (exclusion volume 50 kDa, refilled to 1.5 mL with PBS). Ten equivalents of TCEP were used based on Heredia et al.’s demonstration that one disulfide bridge is opened under these conditions.^[^ ^28^ ^]^ To the reduced protein, 50 µL (1 eq.) of the rebridging stock solution (12 mm in DMSO) was added and stirred for 10 min, producing a yellow coloration upon successful conjugation. The protein was then purified again via dialysis (exclusion volume 50 kDa, refilled to 1.5 mL with PBS) to remove the unconjugated rebridging agent. To reduce the rebridging agent's disulfide bridge, the following steps were repeated three times: the protein conjugate was adjusted to 1.5 mL with PBS buffer, mixed with 100 µL of TCEP stock solution (60 mm in PBS), stirred for 10 min, and rapidly purified via dialysis (exclusion volume 50 kDa, refilled to 1.5 mL with PBS). The protein conjugate was subsequently dialyzed three more times with PBS buffer only and lyophilized.

### Standard Protocol for Preparation of Functionalized BSA‐Polymer Conjugates via TIRP

50 mg (0.77 µmol) BSA‐pNIPAM‐r.b. (**8**) was dissolved in 2 mL PBS buffer and deoxygenated with nitrogen for 20 min. Subsequently, 0.13 mg (0.38 µmol) TPO in 20 µL DMSO was added to the protein solution and deoxygenated for another 20 min. Separately, 25 eq. (18.8 µmol) of the vinyl monomer were dissolved in PBS (50 mg mL^−1^), and 0.05 mol% (0.0095 µmol) Ir‐Cat. in 20 µL DMSO was added before deoxygenation for 20 min. The protein/TPO solution was irradiated for 3 min under a UV lamp (λ = 405 nm) at 114 mW cm^−^^2^. Then, the monomer/Ir‐Cat. mixture was added and irradiated for 60 min at 5700 mW cm^−^^2^. The reaction mixture was purified by dialysis (exclusion volume 50 kDa) and lyophilized.

### DLS Experiments for Particle Formation

Hydrodynamic diameters of thermally induced particles were measured for homovalent conjugates BSA‐pNIPAM (**1**‐**3**) in polystyrene cuvettes from SARSTEDT at concentrations of 1, 0.5, and 0.25 mg mL^−1^ in PBS buffer. Measurements were conducted cyclically, with a forward run (14–54 °C) followed by a return run (54–14 °C). Samples were tested at different heating rates, with a temperature increment of 2 °C and equilibration times of 2, 10, and 20 min between steps.

### DLS Experiments for Particle Formation in Combination with Competition‐Inhibition Assay in Solution of Heterovalent BSA‐Polymer Conjugates and the Mannose and Galactose Mixed Particle

The double‐switchable systems for “I ConA inhibition before particle dissociation” were evaluated using hydrodynamic diameters via DLS measurements in four phases. First, 400 µL (13.8 µm in LBB: 50 mm sodium chloride, 1 mm manganese (II) chloride tetrahydrate, 1 mm calcium chloride, and 10 mm HEPES in ultrapure water, pH 7.4) of the conjugate was placed in a SARSTEDT polystyrene cuvette and heated above the LCST from 28 to 38 °C, with a 2 min equilibration between temperature steps. Subsequently, five measurements were taken at 38 °C with the same equilibration time. In the second phase, 30 µL of ConA solution (5 µm in LBB), preheated to 38 °C, was added, followed by five measurements at 38 °C. In phase three, 100 µL (15 mm) of the pMan (**S3**) inhibitor solution was added at 38 °C, and five more measurements were taken at 38 °C. In the final phase, the temperature was reduced from 38 to 28 °C with a 2 min equilibration between steps.

For the second experiment, “II Lectin stabilized particle below LCST followed by inhibition,” phases one and two were identical to the first experiment. However, in phase three, the temperature was reduced from 38 to 28 °C with five measurements taken with a 2 min equilibration, stabilizing the ConA particle below LCST. In phase four, 100 µL (15 mm) of the pMan (**S3**) inhibitor was added at 28 °C to dissolve the specific binding and the particle.

For the mixed systems, the measurement approach was similar to the four phases of “I ConA inhibition before particle dissociation,” except that phase one included mixtures of conjugates **8** and **9**. Additionally, a measurement was conducted with 11% mannose in an acetate buffer (100 mm sodium acetate, 100 mm sodium chloride, 5 mm calcium chloride, and 5 mm manganese (II) chloride tetrahydrate, pH 5.2). The ratios are provided in the following **Table** **3**:

**Table 3:** Summary of the volumes for the mixed measurements.

Mannose Content	Mannose Conjugate (8) [13.8 µm] [µL]	Galactose Conjugate (9) [13.8 µm] [µL]
50%	200	200
30%	133.3	266.7
20%	80	320
11%	44.4	355.6
6%	23.5	376.5
3%	12.1	387.9

### UV/Vis and Fluorescence Measurements of BSA‐pNIPAM‐r.b. (**8**)

UV/Vis and fluorescence measurements were conducted to verify the conjugation of the rebridging agent to the conjugates. UV/Vis was measured in quartz cuvettes from 200–800 nm. Fluorescence was measured using a microplate reader with an excitation of 396.5 ± 25 nm and an emission range of 480 ± 10 to 605 ± 10 nm. As references, absorbance and fluorescence were independently measured from the conjugate (14 µm in PBS with 10 µL DMSO, deoxygenated with N_2_) and the pure rebridging agent (**6**) (14 µm in PBS). Next, 1 mg of the BSA‐polymer conjugate was dissolved in 1 mL of 140 µm (10 eq.) tris(2‐carboxyethyl)phosphine hydrochloride (TCEP) solution, deoxygenated for 10 min, and its absorbance and fluorescence were measured. Then, 10 µL of the rebridging agent stock solution (1.4 mm in DMSO) was added, and absorbance was measured. As the highest absorbance occurred at ≈20 min, fluorescence was also measured at this time.

### Fluorescence Microscope

Fluorescence images were taken in µ‐Slides 8 Well with glass bottom from ibidi, after 30 min of ozone cleaning. The glass surface was blocked with 1 mg mL^−1^ BSA for 15 min to prevent unspecific interactions, followed by washing five times with LBB. The chamber was then filled with 100 µL pNIPAM‐BSA‐pMan (**8**) (4.6 µm in LBB), heated at 1 °C min^−1^, and equilibrated for 20 min at 38 °C. Subsequently, 5 µL of a 5 µm ConA stock solution in LBB was added and equilibrated for another 20 min. The temperature was reduced to 28 °C at 1 °C min^−1^, and after 20 min of equilibration, 20 µL of the pMan (**S3**) inhibitor stock solution (15 mm in LBB) was added and equilibrated for another 20 min. To test particle formation with ConA added below the LCST, 5 µL ConA (5 µm in LBB) was added to 100 µL pNIPAM‐BSA‐pMan (**8**) (4.6 µm in LBB) at 28 °C, then the temperature was raised to 38 °C at 1 °C min^−1^.

### SEM Imaging

Samples for SEM were prepared similarly to DLS experiments, but after adding lectin to fix the particles, the solution was removed and diluted from 4.6 to 0.46 µm with LBB buffer at 38 °C. The solutions were then dried on 10‐well coverslips, sputtered with gold, and measured.

Standard protocols for a) preparation of BSA‐polymer conjugates via TIRP, b) Pierce‐Protein‐Assay, c) SDS‐PAGE, d) CD measurements, and e) AF4‐LS measurements have already been published by Feldhof et.al.^[^ ^17^ ^]^

## Conflict of Interest

The authors declare no conflict of interest.

## Supporting information



Supporting Information

## Data Availability

The data that support the findings of this study are available from the corresponding author upon reasonable request.
