# Supplementary material for: Site‐Specific Polymer‐Protein‐Polymer Conjugates for the Preparation of Dual Responsive Multilayer Nanoparticles
Source: Small. 2025 Mar 4;21(14):2500531. doi: 10.1002/smll.202500531 (PMC11983248; doi:10.1002/smll.202500531)
Supplement: Supplementary file 1 — Supporting Information [file SMLL-21-2500531-s001.pdf]

NANO · MICRO
small

Supporting Information

for *Small*, DOI 10.1002/smll.202500531

Site-Specific Polymer-Protein-Polymer Conjugates for the Preparation of Dual Responsive Multilayer Nanoparticles

*Melina I. Feldhof, Simon Walber, Sandro Sperzel, Susanne Boye, Ulla I.M. Gerling-Driessen and Laura Hartmann**

Supporting Information

Site-Specific Polymer-Protein-Polymer Conjugates for the Preparation of Dual Responsive Multilayer Nanoparticles

Melina I. Feldhof, Simon Walber, Sandro Sperzel, Susanne Boye, Ulla I.M. Gerling-Driessen, and Laura Hartmann*

M. I. Feldhof, S. Sperzel, Department of Organic and Macromolecular Chemistry, Heinrich-Heine-University Düsseldorf, Universitätsstraße 1, 40225 Düsseldorf, Germany, E-mail: laura.hartmann@hhu.de
Dr. S. Boye, Macromolecular Structure Analysis, Polymer Separation, Leibniz-Institut für Polymerforschung Dresden, Hohe Str. 6, 01069 Dresden, Germany
Simon Walber, Dr. U.I.M. Gerling-Driessen, Prof. Dr. L. Hartmann, Institute for Macromolecular Chemistry, University of Freiburg, Stefan-Meier-Str. 31, D-79104 Freiburg i.Br., Germany
*corresponding author

Table of Contents

1. Experimental Parts 2
  1.1 Materials 2
  1.2 Instrumentation 2
  1.3 General Methods 7
2. Synthesis and analytical data 11
  2.1 Synthesis of carbohydrate monomers **S1**-**S2** 11
  2.2 Synthesis of protein-polymer conjugates **1**-**4** 11
  2.2 Synthsis of rebridging agent 19
  2.3 Conjugation of the rebridging agent to precursor conjugates **1** and **4** 27
  2.4 DLS Particle Experiments 39
  2.5 Inhibition-Competition experiment of double switchable systems 45
  2.6 DLS inhibition Competition Man mix Gal 51
  2.7 Synthesis of inhibitor polymers **S3** and **S4** 65

1. Experimental Parts

1.1 Materials

All used chemicals and solvents were used without further purification and were purchased from commercial sources. Type I water, purified by Barnstead™ MicroPure™ ThermoFisher SCIENTIFIC ultrapure water system, was used unless otherwise mentioned.

Acros Organics: N-isopropylacrylamide ≥ 99%; *Alfa Aesar*: cystamine dihydrochloride ≥ 98%; *Bio-Rad:* 10xtris-glycin/SDS-running buffer, pH 8.6, coomassie brilliant blue, precision plus protein™ kaleidoscope™; *BLDpharm*: 3,4-Dibromo-1h-pyrrole-2,5-dione ≥ 99.4%; *Chemsolute*: sodium hydroxide ≥ 99%; *Eurisotope*: chloroform-d ≥ 99.8%; *Fisher Chemicals: potassium carbonate* ≥ 99%; *Fisher BioReagents™*: 2-(4-(2-hydroxyethyl)-1-piperazinyl)-ethane sulfonic acid (HEPES) ≥ 99%; *Fisher scientific*: Concanavalin A Alexa 647, chloroform ≥ 99.8%; *Honeywell*: sodium sulphate ≥ 99%, acetonitrile ≥ 99.9%, diethyl ether ≥ 99.8%; *J&K*: tris(2-phenylpyridine)iridium(III) ≥ 99%; *Merck*: calcium chloride ≥ 90%, deuterated water ≥ 99.9%, sodium carbonate ≥ 99.7%, sodium chloride 100%, tetrahydrofuran ≥ 99.9%; *PanReac AppliChem*: phosphate buffered saline tablet (PBS), dimethyl sulfoxide (DMSO) ≥ 99.9%; *Sigma Aldrich*: Concanavalin A (ConA), *N*-hydroxyethyl acrylamide ≥ 97.4%, *bovine serum albumin* (BSA) ≥ 96%, DMSO-d_6 ≥ 99.9%, manganese(II) chloride tetrahydrate ≥ 99%; *TCI*: bromoacetyl bromide ≥ 98%,diphenyl(2,4,6-trimethylbenzoyl)phosphine oxide (TPO) ≥ 98%, tris(2-carboxyethyl)phosphine (TCEP) ≥ 98%; *Thermo-Fisher*: pierce BCA protein assay kit; *VWRChemicals*: magnesium sulfate 100%, acetone 100%.

1.2 Instrumentation

Nuclear magnetic Resonance Spectroscopy (NMR):

^{1}H-NMR and ^{13}C-NMR spectrum were measured with a Bruker Avance III 600 (600 MHz) at room temperature. The magnetic field has a strength of 14.10 Tesla. This corresponds to an absorption frequency in the ^{1}H-NMR of 600 MHz and in the ^{13}C-NMR of 150 MHz. Chemical shifts were reported in delta (δ) expressed in oarts per million (ppm) for all ^{1}H-NMR spectrum. D_2O serves as the deuterated solvents for all ^{1}H-NMR spectrum. Parts of non-deuterated solvent was used as an internal standard with δ = 4.79 for HDO. Multiplicities were abbreviated as the following: singlet (s), dublet (d), triplet (t), quartet (q), multiplet (m). The abbreviations CH_3-

primary carbon, CH_2-secondary carbon, CH-tertiary carbon and Cquart-quartary carbon were used for the ^{13}C-NMR and spectrum. The assignment of carbohydrate signals on NMR followed the chronological enumeration of protons/carbon atoms within a monosaccharide starting from the reducing end.

Reversed Phase High Pressure Liquid Chromotography Mass Spectrometry (RP-HPLC-MS): RP-HPLC-MS measurements were performed on Agilent Technologies 1260 Infinity series coupled with Agilent quadrupole mass spectrometer with an Electrospray Ionization (ESI) source operating in a m/z range of 200 to 2000. All spectrum were measured with A: 95% H_2O, 5% ACN, 0.1% formic acid and B: 5% H_2O, 95% ACN, 0.1% formic acid. Separation is performed at 25 °C using an Agilent MZ-Aqua Perfect C_{18} 3 μm (50 x 3.0 mm) column with a linear solvent gradient starting at 100% A, ending at 50% B in 17 minutes at a flow rate of 0.4 mL/minute. Indicated purities were determined by integration of the UV-signal detected by a wavelength detector set to 214 nm with the OpenLab ChemStation software for LC/MS from Agilent Technologies.

Matrix-assisted laser desorption/ionization with time-of-flight mass spectrometer (MALDI-TOF-MS): MALDI-TOF-MS spectrum was performed using a Bruker Daltonics UltfelXtreme device. The matrix used was 2,5 dihydroxybenzoic acid (DHB).

High-resolution-electrospray-ionisation mass-spectrometry (HR-ESI): HR-ESI-MS were performed on UHR-QTOF maXis 4G from Bruker Daltonics.

Dynamic light scattering (DLS): DLS measurements were performed at a Malvern HPPS 3.3 instrument (Malvern Panalytical, Kassel, Germany) equipped with a 633 nm He/Ne-laser with the detector set in backscattering configuration (171°). Protein-polymer conjugates (1-0.25 mg/mL) in 1 cm polystyrene cuvettes were analyzed in a temperature range of 14-54 °C or 28-38 °C with a temperature increment of 2°C and a 2, 10, 20 min equilibration time lag between the temperature steps. For evaluation the autocorrection function, the decay constant and first cumulant were determined by exponential fits the and the hydrodynamic radii were calculated by the Stokes-Einstein relation.

UV/Vis spectroscopy: UV/Vis spectroscopy was performed with a dual-trace spectrometer Specord® 210 Plus from Analytik Jena AG. The measurements were conducted in quartz glass

cuvettes (Starna GmbH d = 1 cm) at 20°C. The instrument was operated with Win ASPECT PLUS software.

UV-Irradiation: UV irradiation of the polymerization was conducted with a UV-LED spot with a wavelength of 405 nm and a maximal irradiance of 114-5700 mW/cm^2 operated by a LEDControl both purchased from Opsytec Dr. Gröbel.

Microplate Reader: Absorption measurements were conducted on CLARIOstar® from BMG LABTECH. Measurements were performed at room temperature and analysed by using BMG Mars software. 96 F-bottom corrugated plates from Greiner BIO-ONE were used for all measurements.

Sodium Dodecyl Sulfate – PolyAcrylamid Gel Electrophoresis (SDS-PAGE): Separations were performed with a 10% acrylamide Page at a voltage of 200 mV and current of 200 mA for about 36 min. 10xTris glycine/SDS buffer, which was diluted down to 1x, serves as the running buffer. Coomassie Brilliant Blue is used to stain the protein bands.

Asymmetrical flow field-flow fractionation with light scattering detection (AF4-LS): AF4-LS measurements were conducted on Eclipse Neon (Waters/Wyatt Technologies) with Agilent pump system (1260, Infinity Series) at 25°C in PBS buffer (1 mM, pH 7.4) according Ref. 2. A short channel with fixed height and regenerated cellulose (cut-off: 10 kDa) was used for all measurements. Detection was performed using the LS detector (DAWN Neon, Waters/Wyatt Technologies λ = 660 nm), RI detector (Optilab T rEX, Wyatt) and UV detectors of various wavelengths (λ = 280, 300, 310, 330 nm). Triplicate measurements with 50 µl injection volume were performed. The following protocol was applied in order to separate the different populations of BSA: channel flow was set to 1.0 mL/min with a detector flow of 0.4 mL/min, focusing was performed with a focus flow of 3.0 mL/min for 6 min followed by an isocratic elution step with a cross flow (Fx) of 3 mL/min for 15 min. The last step proceeds without Fx (0 mL/min) for 20 min. Molar masses were calculated from the LS data applying a Berry fit. Refractive index increments (dn/dc) were determined by manual injection of varied sample concentrations into above mentioned RI detector.

Circular dichroism (CD): CD measurements were conducted on a Jasco J-710 CD spectrometer from Jasco Deutschland GmbH with quartz glass cuvettes (Starna GmbH d = 1 cm) at 20 °C in PBS buffer (pH 7.4). University of Duisburg-Essen, Center for Medical Biotechnology and Structural and Medical Biochemistry, AG Bayer.

Fluorescence microscopy: The microscopic fluorescence images were generated by Epifluorescence IX83 microscope from Olympus (Olympus Corporation, Tokyo, JPN) equipped with a 60x, NA 1.25 objective (Olympus Corporation, 60x Planfluor PH3 UPLFLN60XOIPH/1.25, Tokyo, JPN). The temperature was set using Tempcontroller 2000-2 (PeCon GmbH, Erbach, GER). F49-641 640/30 ET Bandpass Excitation Filter, F47-690 690/50 ET Bandpass Emission Filter and F48-660 Beam Splitter T660 LPXR were used as beam splitters. Heinrich-Heine Universität Düsseldorf, Institute of Experimental Medical Physics AG Monzel.

SEM: SEM Images were generated by ZEISS SUPRA 55V high-resolution scanning electron microscope has a field emission cathode (FEG: Field Emission Gun) with VP (variable pressure) mode from 2 - 133 Pascal, adjustable in 1 Pascal steps with an SE2 detector. We would like to acknowledge the Center for Advanced Imaging (CAi) at Heinrich Heine University and especially Steffen Köhler for performing the sample preparation and the imaging at the transmission electron microscope.

Infrared spectroscopy (IR): IR spectroscopic measurements were conducted on a Nicolet iS50 FT-IR from Thermo Scientific with. The analysis is carried out with the OMNIC spectroscopy software from thermoscientific.

Aqueous gel permeation chromatography (GPC): GPC measurements of the protein-polymer conjugates were conducted using an Agilent 1200 series HPLC system equipped with a GE Healthcare GPC column (Superdex 75 10/300). The eluent used was MilliQ water containing 50 mM NaH_2PO_4, 150 mM NaCl, 250 ppm NaN_3 and pH 7 with 30 vol% acetonitrile. was used. This was passed through an inline membrane filter of 0.1 um, with filtered at a flow rate of 0.8 mL/min. The average molecular weights were determined by multi-angle light scattering. determined. A miniDAWN TREOS from Wyatt was used for this purpose. The detection was performed with an Optilab rEX from Wyatt.

Aqueous SEC-MALS-RI: SEC-MALS-RI measurements of the polymeric Inhibitors *S7* and *S8* were conduted using Wyatt systems, isocratic pump, degasser, AS of 1260 Infinity series, LS detector: DAWN Heleos-II (Wyatt), λ = 660 nm RI detector: Optilab T-rEX (Wyatt), λ = 660 nm UV detector: UV detector SPD-M20 (Shimadzu): With variable wavelengths Channel (1) λ = 220 nm, Channel (2) λ = 230 nm Channel (3) λ = 250 nm, Channel (4) λ = 290 nm, Flow rate: 1 mL/min, temperature: 25 °C, Concentration: 3-4 mg/mL, Injection volume: 100 µL. Precolumn (50 mm, 2 x 160 Å of 300 mm and 1000 Å of 300 mm), two main columns (GE Healthcare GPC column Superdex 75 10/300, 8 mm diameter and 5 µm particle size), eluent: MilliQ water:acetonitrile 7:3 (v/v), 50 mM, NaH_2PO_4, 150 mM NaCl and 250 ppm NaN_3, pH = 7.0.

1.3 General Methods

Standard protocols for a) preparation of BSA-polymer conjugates via TIRP, b) Pierce-Protein-Assay, c) SDS-PAGE, d) CD measurements and e) AF4-LS measurements have already been published by Feldhof et.al.[1]

Ellman's Assay: The measurements were carried out in triplicates, for each measurement: 196 µL of Ellman's buffer (0.1M sodium phosphate, pH 8.0, containing 1mM EDTA) was mixed with 20 µL of the protein stock solution or the reduced form with 10eq. TCEP (0.75 mM in Ellman's buffer) followed by 4 µL of the Ellman's stock solution (1 mg/mL in Ellman's buffer) and incubated for 15 min. The absorbance was measured at 412 nm and the amount of accessible thiols was determined based on a previously established calibration curve. For unmodified BSA, the measured value of 54 ± 8% aligns with previously values in the literature.[1,2] When BSA is reduced with TCEP, the value increases to 257 ± 8%, representing a 200% rise compared to the untreated sample. This observation indicates that using 10 eq. of TCEP results in the exposure of only one disulfide bond, making two additional thiol groups available. This value is also supported by the literature.[2] Data were evaluated using one-way ANOVA analysis followed by Bonferroni correction and shows a significant difference (*<0.05).

Standard protocol for introducing the rebridging agent on previous synthesized BSA-polymer conjugates and reduction of the disulfide bridge from the rebridging agent: 40 mg (0.6 µmol) of BSA-p(HEAA) (**4**) or BSA-pNIPAM (**1**) conjugate was dissolved in 1.5 mL PBS buffer. To this, 100 µL of a 60 mM (10 eq.) tris(2-carboxyethyl)phosphine (TCEP) stock solution in PBS buffer was added and stirred for 10 min at room temperature. The solution was briefly purified via dialysis (exclusion volume 50 kDa, refilled to 1.5 mL with PBS), followed by a second addition of 100 µL of TCEP stock solution (60 mM in PBS) and further stirring for 10 min at room temperature. Remaining TCEP residues were completely removed by repeated purification with deoxygenated PBS buffer via dialysis (exclusion volume 50 kDa, refilled to 1.5 mL with PBS). Ten equivalents of TCEP were used based on Heredia et al.'s demonstration that one disulfide bridge is opened under these conditions.[2] To the reduced protein, 50 µL (1 eq.) of the rebridging stock solution (12 mM in DMSO) was added and stirred for 10 min, producing a yellow coloration upon successful conjugation. The protein was then purified again via dialysis (exclusion volume 50 kDa, refilled to 1.5 mL with PBS) to remove unconjugated rebridging agent. To reduce the

rebridging agent's disulfide bridge, the following steps were repeated three times: the protein conjugate was adjusted to 1.5 mL with PBS buffer, mixed with 100 µL of TCEP stock solution (60 mM in PBS), stirred for 10 min, and rapidly purified via dialysis (exclusion volume 50 kDa, refilled to 1.5 mL with PBS). The protein conjugate was subsequently dialyzed three more times with PBS buffer only and lyophilized.

Standard protocol for preparation of functionalized BSA-polymer conjugates via TIRP: 50 mg (0.77 µmol) BSA-pNIPAM-r.b. (**8**) was dissolved in 2 mL PBS buffer and deoxygenated with nitrogen for 20 minutes. Subsequently, 0.13 mg (0.38 µmol) TPO in 20 µL DMSO was added to the protein solution and deoxygenated for another 20 minutes. Separately, 25 eq. (18.8 µmol) of the vinyl monomer were dissolved in PBS (50 mg/mL), and 0.05 mol% (0.0095 µmol) Ir-Cat. in 20 µL DMSO was added before deoxygenation for 20 minutes. The protein/TPO solution was irradiated for 3 minutes under a UV lamp (λ = 405 nm) at 114 mW/cm². Then, the monomer/Ir-Cat. mixture was added and irradiated for 60 minutes at 5700 mW/cm². The reaction mixture was purified by dialysis (exclusion volume 50 kDa) and lyophilized.

DLS Experiments for particle formation: Hydrodynamic diameters of thermally induced particles were measured for homovalent conjugates BSA-pNIPAM (**1**-**3**) in polystyrene cuvettes from SARSTEDT at concentrations of 1 mg/mL, 0.5 mg/mL, and 0.25 mg/mL in PBS buffer. Measurements were conducted cyclically, with a forward run (14-54 °C) followed by a return run (54-14 °C). Samples were tested at different heating rates, with a temperature increment of 2 °C and equilibration times of 2, 10, and 20 minutes between steps.

DLS experiments for particle formation in combination with competition-inhibition assay in solution of heterovalent BSA-polymer conjugates and the mannose and galactose mixed particle: The double-switchable systems for "I ConA inhibition before particle dissociation" were evaluated using hydrodynamic diameters via DLS measurements in four phases. First, 400 µL (13.8 µM in LBB: 50 mM sodium chloride, 1 mM manganese (II) chloride tetrahydrate, 1 mM calcium chloride, and 10 mM HEPES in ultrapure water, pH 7.4) of the conjugate was placed in a SARSTEDT polystyrene cuvette and heated above the LCST from 28-38 °C, with a 2-minute equilibration between temperature steps. Subsequently, five measurements were taken at 38 °C with the same equilibration time. In the second phase, 30 µL of ConA solution (5 µM in LBB),

preheated to 38 °C, was added, followed by five measurements at 38 °C. In phase three, 100 µL (15 mM) of the pMan (**S3**) inhibitor solution was added at 38 °C, and five more measurements were taken at 38 °C. In the final phase, the temperature was reduced from 38 °C to 28 °C with a 2-minute equilibration between steps.

For the second experiment, “II Lectin stabilized particle below LCST followed by inhibition,” phases one and two were identical to the first experiment. However, in phase three, the temperature was reduced from 38 °C to 28 °C with five measurements taken with a 2-minute equilibration, stabilizing the ConA particle below LCST. In phase four, 100 µL (15 mM) of the pMan (**S3**) inhibitor was added at 28 °C to dissolve the specific binding and the particle.

For the mixed systems, the measurement approach was similar to the four phases of "I ConA inhibition before particle dissociation," except that phase one included mixtures of conjugates **8** and **9**. Additionally, a measurement was conducted with 11% mannose in an acetate buffer (100 mM sodium acetate, 100 mM sodium chloride, 5 mM calcium chloride, and 5 mM manganese (II) chloride tetrahydrate, pH 5.2). The ratios are provided in the following table:

Table S1: Summary of the volumes for the mixed measurements.

mannose content	mannose conjugate (8) (13.8 µM) [µL]	galactose conjugate (9) (13.8 µM) [µL]
**50%**	200	200
**30%**	133.3	266.7
**20%**	80	320
**11%**	44.4	355.6
**6%**	23.5	376.5
**3%**	12.1	387.9

UV/Vis and fluorescence measurements of BSA-pNIPAM-r.b. (*8*): UV/Vis and fluorescence measurements were conducted to verify the conjugation of the rebridging agent to the conjugates. UV/Vis was measured in quartz cuvettes from 200-800 nm. Fluorescence was measured using a microplate reader with an excitation of 396.5 ± 25 nm and an emission range of 480 ± 10 nm to 605 ± 10 nm. As references, absorbance and fluorescence were independently measured from the conjugate (14 µM in PBS with 10 µL DMSO, deoxygenated with N_2) and the pure rebridging agent (**6**) (14 µM in PBS). Next, 1 mg of the BSA-polymer conjugate was dissolved in 1 mL of 140 µM (10 eq.) tris(2-carboxyethyl)phosphine hydrochloride (TCEP) solution, deoxygenated for 10 min, and its absorbance and fluorescence were measured. Then, 10 µL of the rebridging agent stock

solution (1.4 mM in DMSO) was added, and absorbance was measured. As the highest absorbance occurred at approximately 20 min, fluorescence was also measured at this time.

Fluorescence microscope: Fluorescence images were taken in µ-Slides 8 Well with glass bottom from ibidi, after 30 min of ozone cleaning. The glass surface was blocked with 1 mg/mL BSA for 15 min to prevent unspecific interactions, followed by washing five times with LBB. The chamber was then filled with 100 µL pNIPAM-BSA-pMan (**8**) (4.6 µM in LBB), heated at 1 °C/min, and equilibrated for 20 min at 38 °C. Subsequently, 5 µL of a 5 µM ConA stock solution in LBB was added and equilibrated for another 20 min. The temperature was reduced to 28 °C at 1 °C/min, and after 20 min of equilibration, 20 µL of the pMan (**S3**) inhibitor stock solution (15 mM in LBB) was added and equilibrated for another 20 min. To test particle formation with ConA added below the LCST, 5 µL ConA (5 µM in LBB) was added to 100 µL pNIPAM-BSA-pMan (**8**) (4.6 µM in LBB) at 28 °C, then the temperature was raised to 38 °C at 1 °C/min.

SEM imaging: Samples for SEM were prepared similarly to DLS experiments, but after adding lectin to fix the particles, the solution was removed and diluted from 4.6 µM to 0.46 µM with LBB buffer at 38 °C. The solutions were then dried on 10-well coverslips, sputtered with gold, and measured.

2. Synthesis and analytical data

2.1 Synthesis of carbohydrate monomers S1-S2

S1 α-D-Mannopyranosyl-ethylacrylamid (Man) and

S2 β-D-Galaktopyranosyl-ethylacrylamid (Gal)

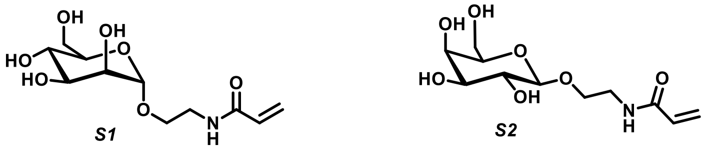

The syntheses of structures S1 and S2 have already been published by Feldhof et.al.[1]

2.2 Synthesis of protein-polymer conjugates 1-4

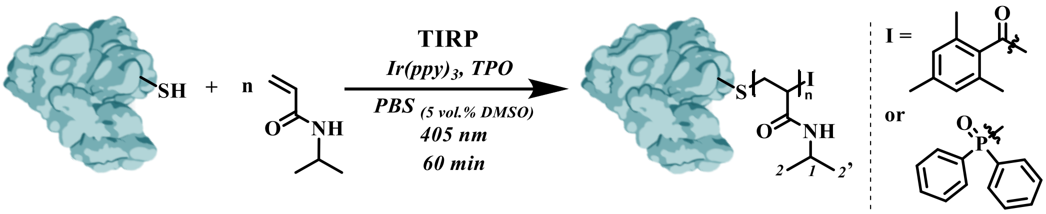

200 mg (3 µmol) of *bovine serum albumin* was dissolved in 5 mL of deoxygenated phosphate buffered saline (PBS buffer). Separate solutions *I-III* were prepared. *I*: **A** mg **B** µmol) of *N*-isopropylacrylaminde (NIPAM) in **C** µL of PBS buffer (50 mg/mL), *II*: **D** mg (**E** µmol) of catalyst tris(2 phenylpyridine)iridium (Ir-Cat.) in 0.1 mL of dimethyl sulfoxide, *III*: 0.54 mg (1.5 µmol) of diphenyl(2,4,6-trimethylbenzoyl)phosphine oxide (TPO) in 0.1 mL of dimethyl sulfoxide. Ir-Cat. solution *II* is then added to monomer solution *I* and deoxygenated with nitrogen for 10 minutes. In parallel, the TPO solution *III* is slowly dropped to the protein solution and also deoxygenated with nitrogen for 10 minutes before it was irradiated in the focus of the UV lamp (405 nm) for 3 minutes at an intensity of 1.15 mW/cm^2. Subsequently, the mixture of monomer *I* and catalyst solution *II* was also added to the protein and the entire mixture was irradiated for another 60 minutes at 5.22 mW/cm^2 intensity. The reaction mixture was purified by dialysis (exclusion volume 50 kDa) and then lyophilized. A white, fluffy powder was obtained.

Table S2: Formulation values of the monomers and buffer volumes of polymerizations.

structure	mass of monomer A [mg]	amount of monomer B [µmol]	PBS buffer volume C [µL]	mass of Ir-Kat D [mg]	amount of monomer E [µmol]	yields [mg]
**1**	17.0	150	340	0.05	0.075	209.44
**2**	34.2	300	680	0.10	0.15	211.31
**3**	68.3	600	1360	0.20	0.30	243.50

1 – BSA-pNIPAM$_{50}$ conjugate

The characterization of this structure was carried out completely in Feldhof et al.[1]

2 – BSA-pNIPAM$_{100}$ conjugate

^{1}H-NMR (600 MHz, D_2O, 297.9 K) δ (ppm) 9.36 – 5.69 (m, *H_{BSA}*), 4.59 – 0.00 (m, H_{BSA}, *$H_{NIPAM\text{-}side\ chain}$*, *$H_{NIPAM\text{-}backbone}$*, overlap with water peak), 3.91 (m_{br}, *H1*), 1.67 (s_{br}, *H2, H´2*).

AF4-LS (1 mM PBS, pH = 7.4, 298,15 K, monomer fraction): Mn = 75.8 kDa, Mw = 81.6 kDa, Đ = 1.08, Rh = 2.6 nm.

IR (ATR) $\tilde{\nu}$ = 1066.66 cm^{-1} (deformation C-OH). All other wavelengths identical to BSA.

CD: area 215 nm – 245 nm: 221 nm, -18.84 mdeg, area 198 nm – 215 nm: 210.0 nm, -18.94 mdeg.

SDS PAGE see Fig. S10.

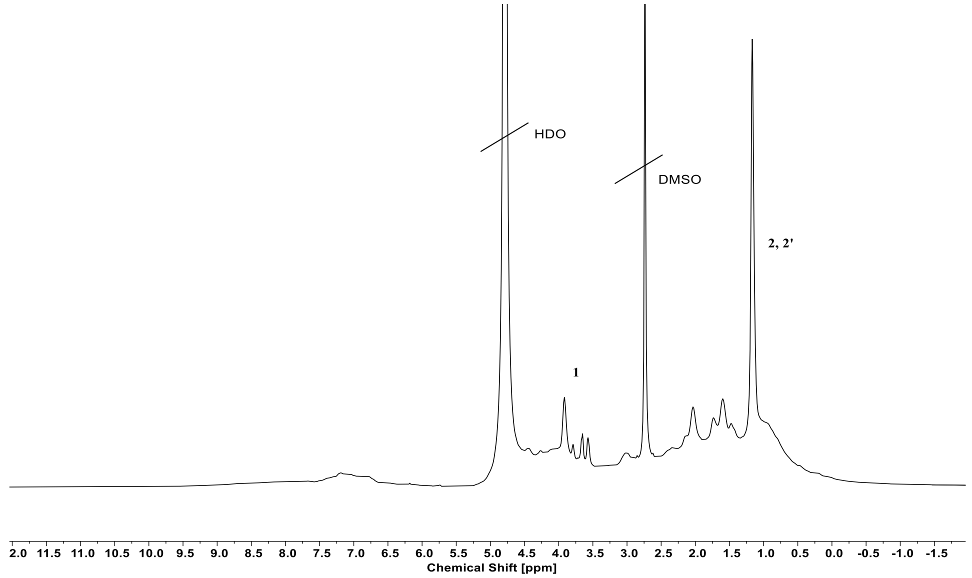

*Fig. S1: ^{1}H-NMR spectrum (600 MHz, D_2O, 297.9 K) of BSA-pNIPAM$_{100}$ conjugate (**2**).*

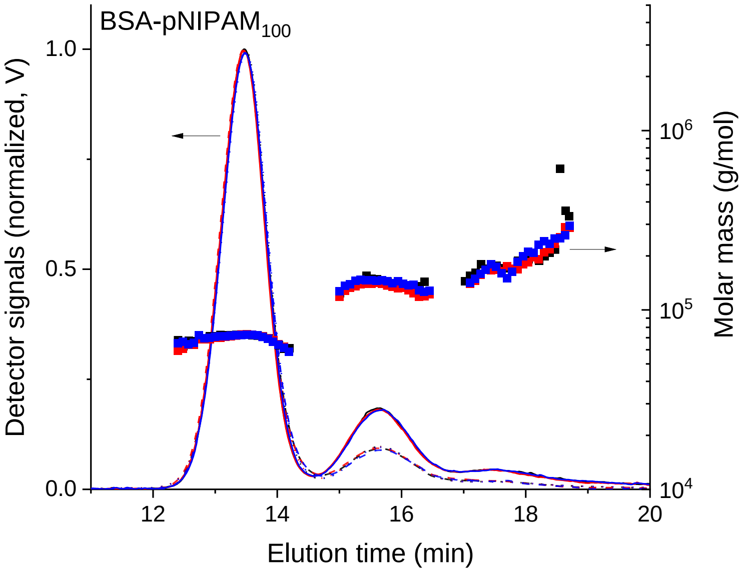

*Fig. S2: Triplicate measurements and presentation of fractograms of BSA-pNIPAM$_{100}$ conjugate (**2**) opt. separation, RI (dashed line), UV (dotted line) and LS signal (solid line), molar masses (symbols) vs. elution time.*

Table S3: Results of the triplicate AF4-LS measurements.

**measurement**	**M_n***1 (kg/mol)	**M_w***1 (kg/mol)	**Đ** (M_w/M_n)
**1.** (50 µl)	76.4	82.8	1.08
**2.** (50 µl)	75.5	80.8	1.07
**3.** (50 µl)	75.5	81.3	1.08
**average (whole peak)**	**75.8**	**81.6**	**1.08**

*1 dn/dc = 0.172 ml/g

Table S4: Analysis of the individual fractions of AF4-LS. The values shown are average values from the triplicate measurements.

**fraction**	**M_n***1 (kg/mol)	**M_w***1 (kg/mol)	**Đ** (M_w/M_n)	**mass fraction** (%)	**mol fraction** (%)
1 - monomer	72.1	72.1	1.00	85.5	92.5
2 – dimer	143	143	1.00	11.8	6.3
3- trimer / multimer	176	177	1.01	2.7	1.2

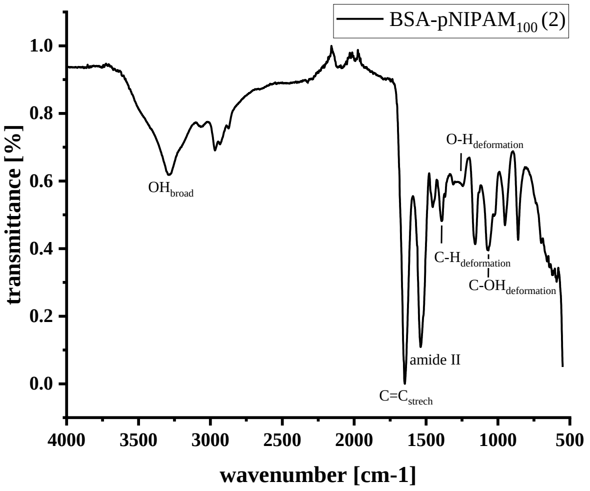

*Fig. S3: IR spectrum BSA-pNIPAM100 conjugate (**2**).*

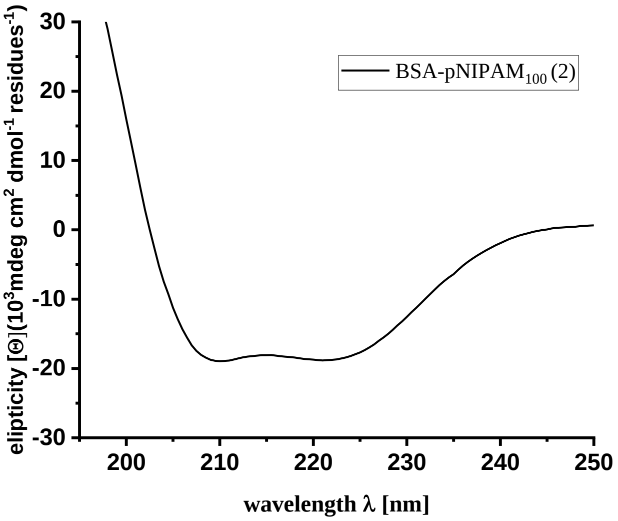

Fig. S4: CD spectrum of BSA-pNIPAM conjugate (2).

3 – BSA-pNIPAM₂₀₀ conjugate

^{1}H-NMR (600 MHz, D_2O, 297.9 K) δ (ppm) 9.18 – 6.20 (m, H_{BSA}), 4.48 – 0.00 (m, H_{BSA}, $H_{NIPAM\text{-}side\ chain}$, $H_{NIPAM\text{-}backbone}$, overlap with water peak), 3.92 (m_{br}, *H1*), 1.67 (m_{br}, *H2, H´2*).

AF4-LS (1 mM PBS, pH = 7.4, 298,15 K, monomer fraction): Mn = 74.8 kDa, Mw = 80.9 kDa, Đ = 1.08, Rh = 2.6 nm.

Aqueous SEC: M_n: 96 kDa with 1,045 Đ.

IR (ATR) $\tilde{\nu}$ = 1077.21 cm^{-1} (deformation C-OH). All other wavelengths identical to BSA.

CD: area 215 nm – 245 nm: 218.5 nm, -28.05 mdeg, area 198 nm – 215 nm: 208.0 nm, -30.4 mdeg.

SDS PAGE see Fig. S10.

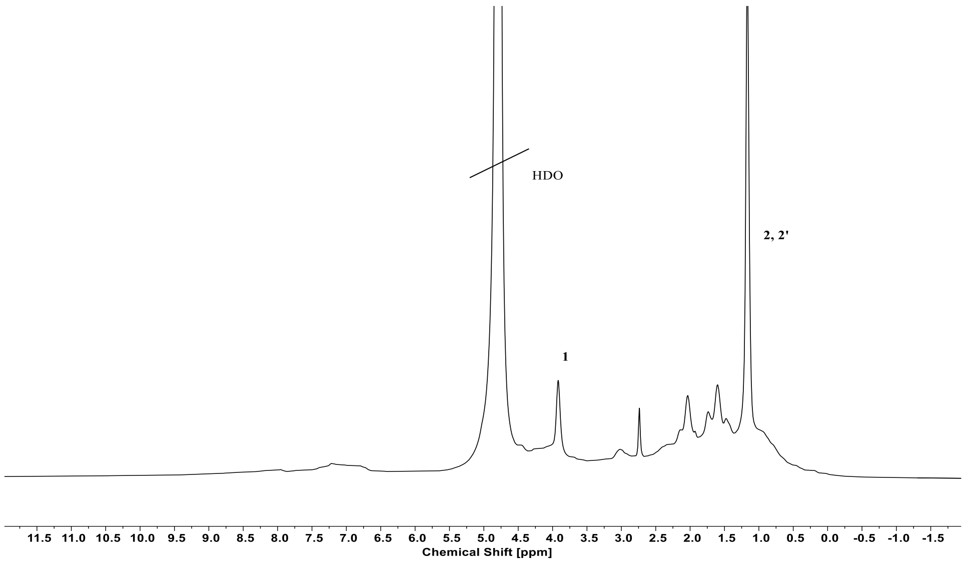

*Fig. S5: ^{1}H-NMR spectrum (600 MHz, D_2O, 297.9 K) of BSA-pNIPAM₂₀₀ conjugate (**3**).*

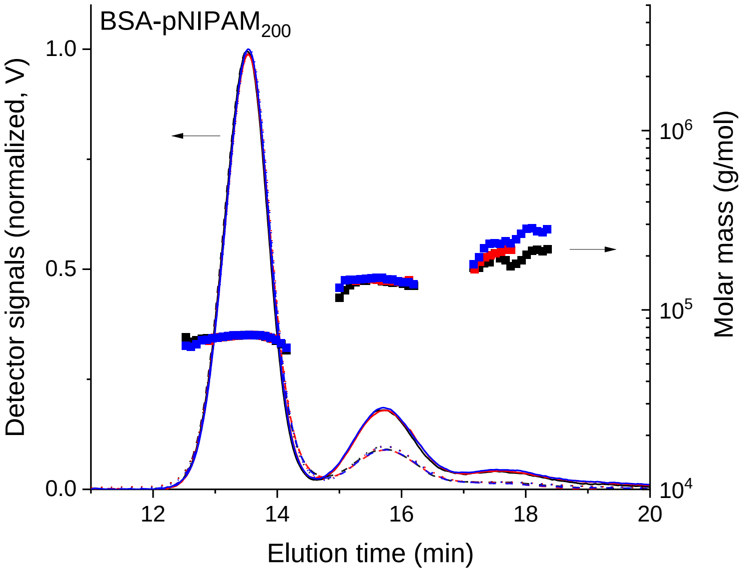

*Fig. S6: Triplicate measurements and presentation of fractograms of BSA-pNIPAM$_{100}$ conjugate (**2**) opt. separation, RI (dashed line), UV (dotted line) and LS signal (solid line), molar masses (symbols) vs. elution time.*

Table S5: Results of the triplicate AF4-LS measurements.

**Measurement**	**M_n*1** (kg/mol)	**M_w*1** (kg/mol)	**Đ** (M_w/M_n)
**1.** (50 µl)	74.5	80.0	1.07
**2.** (50 µl)	74.7	80.7	1.08
**3.** (50 µl)	75.2	81.9	1.09
**average (whole peak)**	**74.8**	**80.9**	**1.08**

*1 dn/dc = 0.172 ml/g

Table S6: Analysis of the individual fractions of AF4-LS. The values shown are average values from the triplicate measurements.

**fraction**	**M_n*1** (kg/mol)	**M_w*1** (kg/mol)	**Đ** (M_w/M_n)	**mass fraction** (%)	**mol fraction** (%)
1 - monomer	71.6	71.6	1.00	86.6	93.5
2 – dimer	146	146	1.00	10.8	5.9
3- trimer / multimer	209	211	1.01	2.6	0.6

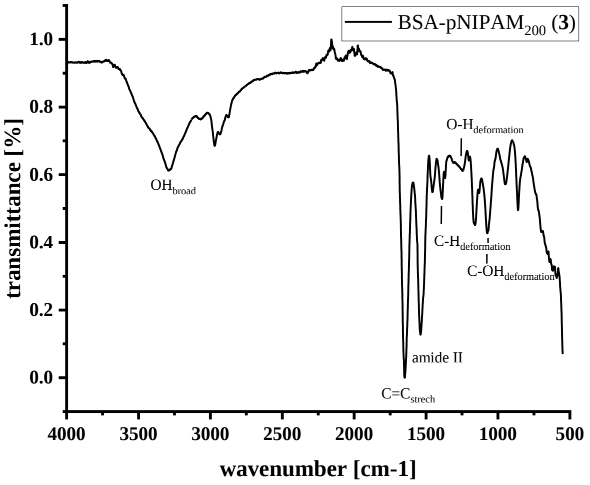

*Fig. S7: IR spectrum BSA-pNIPAM$_{200}$ conjugate (**3**).*

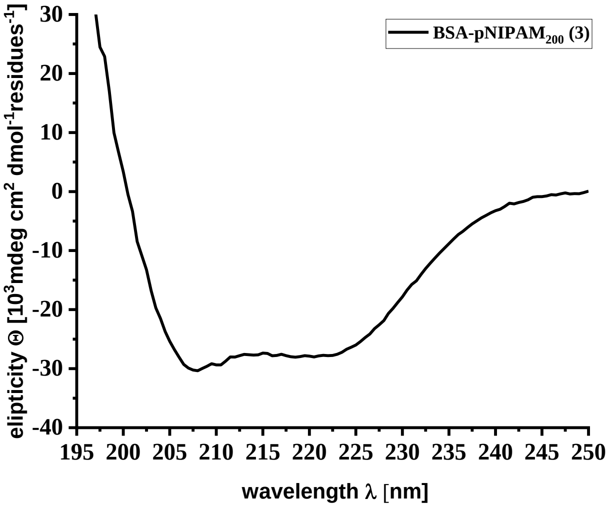

*Fig. S8: CD spectrum of BSA-pNIPAM$_{200}$ conjugate (**3**).*

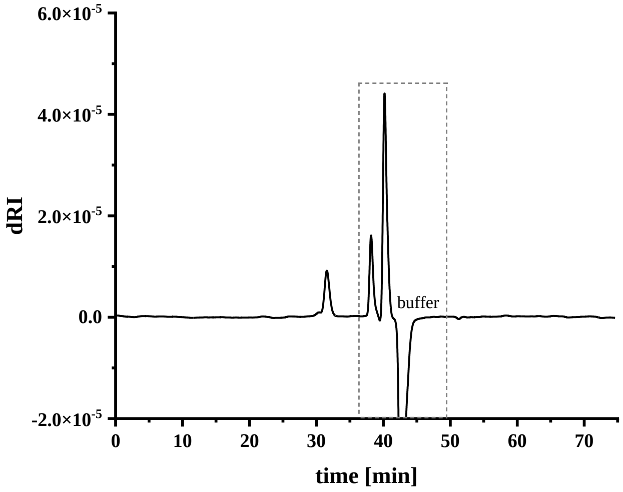

*Fig. S9: Aqueous SEC-MALS measurement of BSA-pNIPAM$_{200}$ (**3**).*

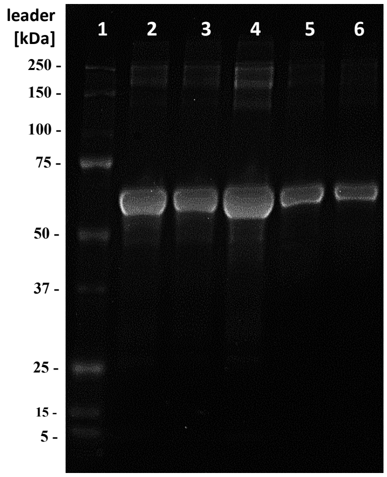

lane	*loading*
1	protein leader
2	BSA_{native}
3	BSA-pHEAA (4)
4	BSA-pNIPAM (1)
5	BSA-pNIPAM (2)
6	BSA-pNIPAM (3)

*Fig. S10: SDS-PAGE of BSA_{native} BSA-pNIPAM$_{50}$ (**1**), BSA-pNIPAM$_{100}$ (**2**), BSA-pNIPAM$_{200}$ (**3**). Since SDS-PAGE estimates the molecular weight of denatured proteins, the observed differences in the molecular weights of individual protein-polymer conjugates were marginal and did not reflect the actual molecular weights. This is expected, as polymers behave differently from proteins under denaturing conditions. The polymers likely remain highly coiled, not significantly influencing the denatured protein's behavior in SDS-PAGE.*

4 – BSA-pHEAA$_{50}$ conjugate

The characterization of this structure was carried out completely in Feldhof et al.[1]

2.2 Synthsis of rebridging agent

5 – Synthesis of N,N' (disulfanediylbis(ethan2,1diyl))-bis(2bromo-acetamide)

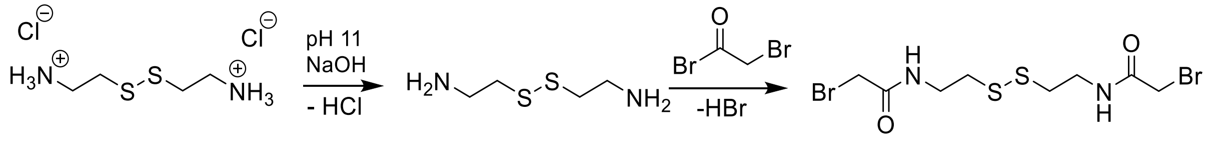

1 g (4.44 mmol) of cystamine dihydrochloride was added to 10 mL of water and adjusted to a pH value of 10 with sodium hydroxide. The cystamine was extracted four times with 40 mL chloroform each time. Then 0.81 mL (9.3 mmol) bromoacetyl bromide was slowly added and the reaction solution was stirred for 2 h at room temperature (21 °C). The product mixture was washed three times with water and the organic phase was dried using magnesium sulfate. Finally, the solvent was removed under reduced pressure. A colorless powder with a yield of 0.7 g (40%) was obtained.

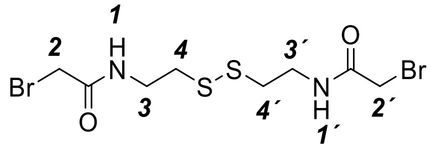

^{1}**H-NMR** (600 MHz, $CDCl_3$, 297.9 K) δ (ppm) 7.03 (s_{br}, 2H, *H1, H1´*), 3.90 (s, 4H, *H2, H2´*), 3.69 – 3.57 (q, ^{3}J = 6.3 Hz, 4H, *H3, H3´*), 2.91 – 2:77 (t, ^{3}J = 6.4 Hz, 4H, *H4, H4´*).

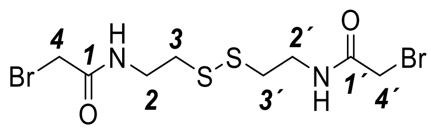

^{13}**C-NMR** (600 MHz, $CDCl_3$, 297.9 K) δ (ppm) 166.18 (2C, *C1, C1´*), 39.14 (2C, *C2, C2´*), 37.41 (2C, *C3, C3´*), 29.17 (2C, *C4, C4´*).

LC-MS: m/z calculated for $C_8H_{14}Br_2N_2O_2S_2$ $[M+H]^+$ 394.88, found 394.80 und $[M+Na]^+$ 416.88, found 416.80 certain relative purity: >95%.

HR-ESI-MS: m/z calculated for $C_8H_{14}Br_2N_2O_2S_2$ $[M+H]^+$ 394.88 found 394.89.

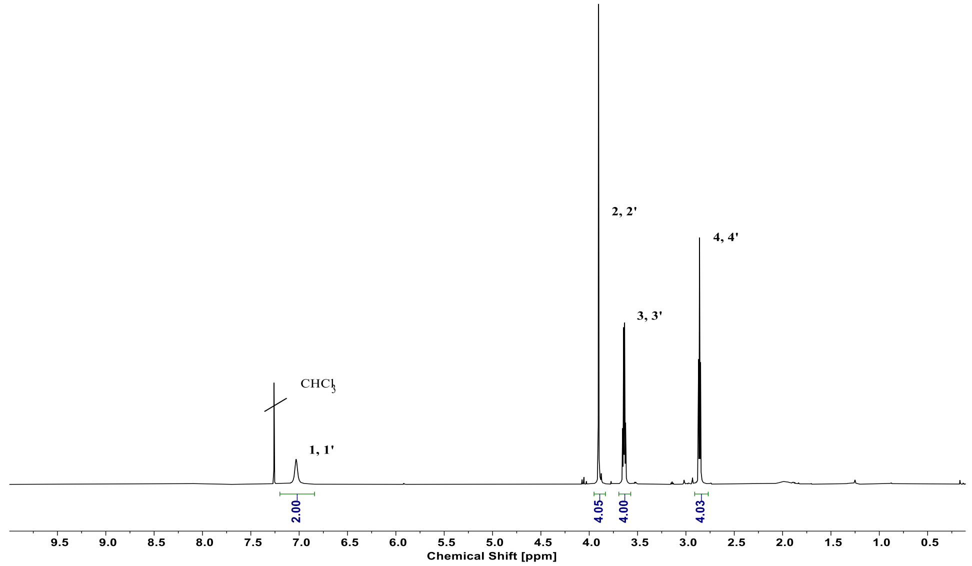

*Fig. S11. ^{1}H-NMR spectrum (600 MHz, $CDCl_3$, 297.9 K) of N,N' (disulfanediylbis(ethan2,1diyl))-bis(2bromo-acetamide) (**5**).*

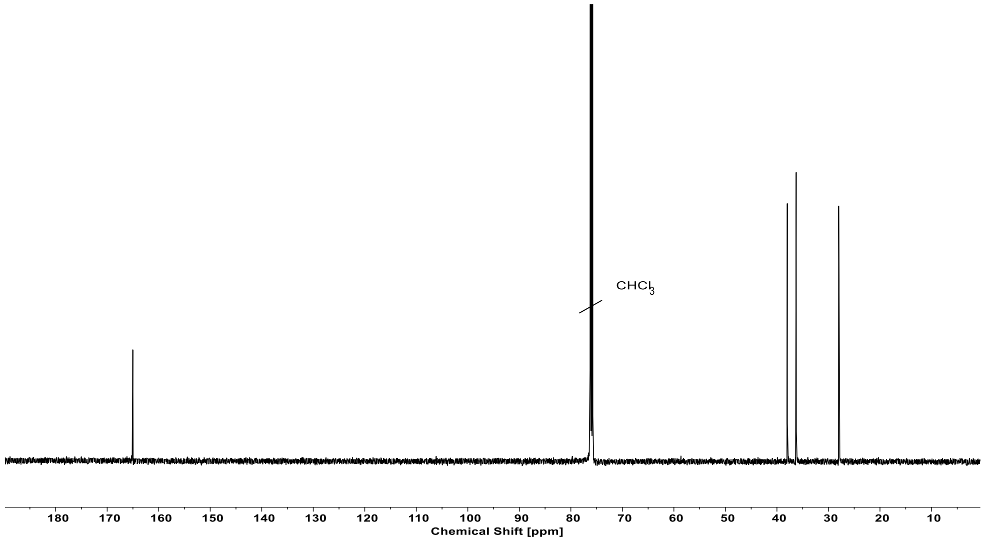

*Fig. S12: ^{13}C-NMR spectrum (150 MHz, $CDCl_3$, 297.9 K) of N,N' (disulfanediylbis(ethan2,1diyl))-bis(2bromo-acetamide) (**5**).*

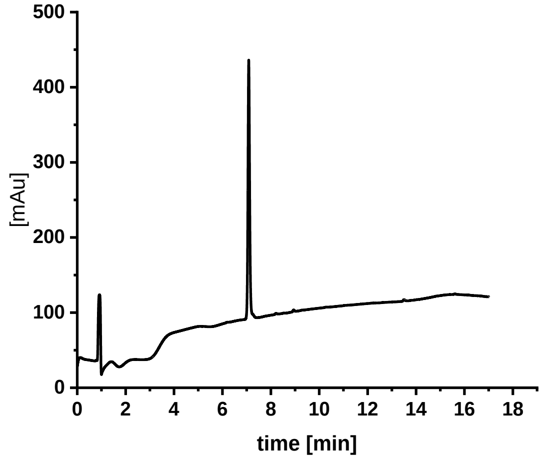

*Fig. S13: RP-HPCL chromatogram of the N,N' (disulfanediylbis(ethane2,1diyl))-bis(2bromoacetamide) (**5**) (gradient from 5 to 95vol.% acetonitrile/water with 0.1vol.% formic acid, run time: 17 min).*

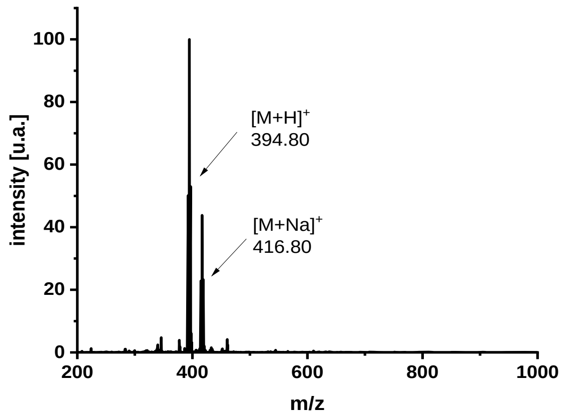

*Fig. S14. ESI mass spectrum of N,N' (disulfanediylbis(ethane2,1diyl))-bis(2bromoacetamide)(**5**) at t = 7.2 min (gradient from 5 to 95 vol.% acetonitrile/water with 0.1 vol.% formic acid, run time: 17 min).*

Acquisition Parameter

Source Type	ESI	Ion Polarity	Positive	Set Nebulizer	0.3 Bar
Focus	Not active	Set Capillary	4000 V	Set Dry Heater	180 °C
Scan Begin	50 m/z	Set End Plate Offset	-500 V	Set Dry Gas	4.0 l/min
Scan End	1500 m/z	Set Collision Cell RF	600.0 Vpp	Set Divert Valve	Source

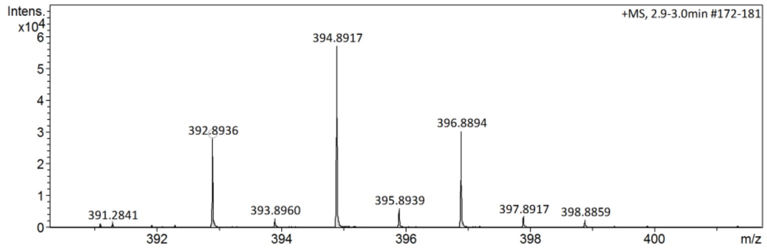

*Fig. S15: HR-ESI-MS spectrum of N,N' (disulfanediylbis(ethane2,1diyl))-bis(2bromoacetamide) (**5**).*

6 – N,N'-(disulfanediylbis(ethan-2,1-diyl))bis(2-(3,4-dibromo-2,5-dioxo-2,5-dihydro-1H-pyrrol-1-yl)acetamide (rebridging agent)

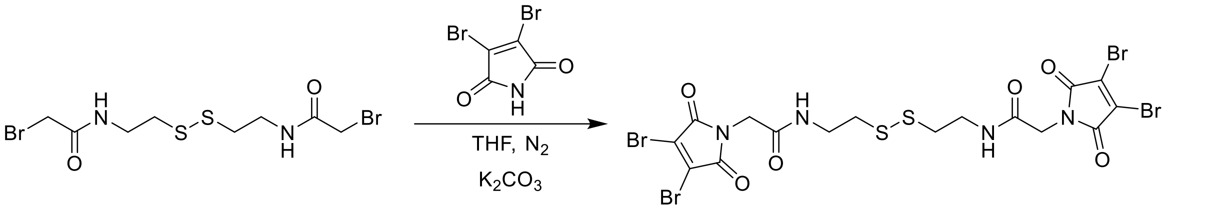

3 g (11.77 mmol) of 3,4-dibromopyrrole-2,5-dione and 8.04 g (58.2 mmol) of potassium carbonate were placed in 30 mL of dry tetrahydrofuran and purged with nitrogen for 15 min. 1.53 g (3.99 mmol) of N,N'-(disulfanediylbis(ethane-2,1-diyl))bis(2-bromoacetamide) was suspended in 15 mL of tetrahydrofuran and slowly added to the reaction solution. The reaction mixture was purged with nitrogen for a further 2 h and stirred overnight at room temperature (21 °C). The solvent was removed under reduced pressure, the oily residue was taken up with 50 mL chloroform, washed three times with 30 mL water each time, the organic phase was dried over magnesium sulfate and the solvent was removed under reduced pressure. The product mixture was recrystallized in ethyl acetate and a white powder with a yield of 0.21 g (7%) was obtained.

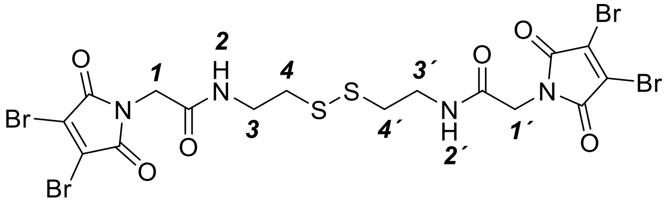

^{1}H-NMR (600 MHz, DMSO-*d₆*, 297.9 K) δ (ppm) 8.54 – 8.3 (t, ^3J = 5.7 Hz, 2H, *H2, H2´*), 4.10 (s, 4H, *H1, H1´),* 3.48 – 3.21 (q, ^3J = 6.5 Hz, 4H, *H3, H3´*), 2.89 – 2.66 (t, ^3J = 6.8 Hz, 4H, *H4, H4´*).

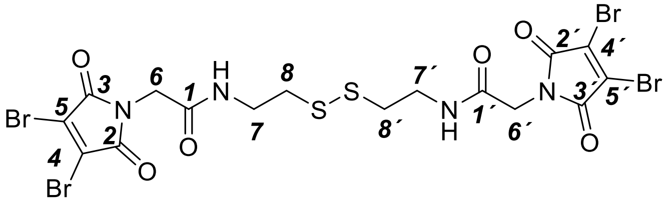

^{13}C-NMR (600 MHz, DMSO-d_6, 297.9 K) δ (ppm) 165.76 (2C, *C1, C1´*), 163.93 (4C, *C2, C2´,C3, C3´),* 129.56 (4C, *C4, C4´, C5, C5´*), 41.39 (2C, *C6, C6´*), 38.09 (2C, *C7, C7´*), 36.75 (2C, *C8, C8´*).

LC-MS: m/z calculated for $C_{16}H_{14}Br_4N_4O_6S_2$ $[M+H]^+$ 742.70, found 742.70, certain relative purity: 94%.

HR-ESI-MS: m/z calculated for $C_{16}H_{14}Br_4N_4O_6S_2$ $[M+H]^+$ 742.70 found 742.71.

MALDI-TOF: m/z calculated mass $[M+H]^+$ 742.70 found 742.71, m/z calculated mass $[M+Na]^+$ 762.70 found 762.63.

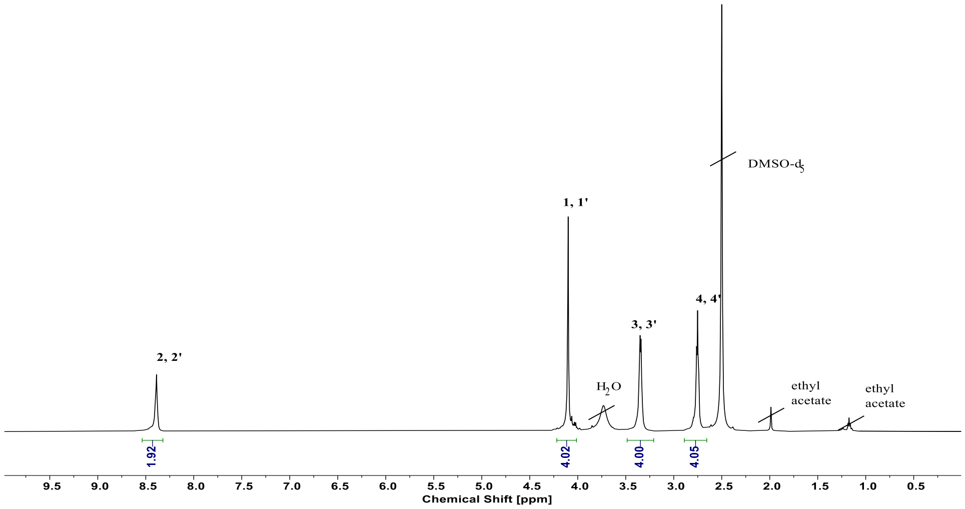

*Fig. S16: ^{1}H-NMR spectrum (600 MHz, DMSO-d_6, 297.9 K) of N,N'-(disulfanediylbis(ethan-2,1-diyl))bis(2-(3,4-dibromo-2,5-dioxo-2,5-dihydro-1H-pyrrol-1-yl)acetamide (rebridging agent) (**6**).*

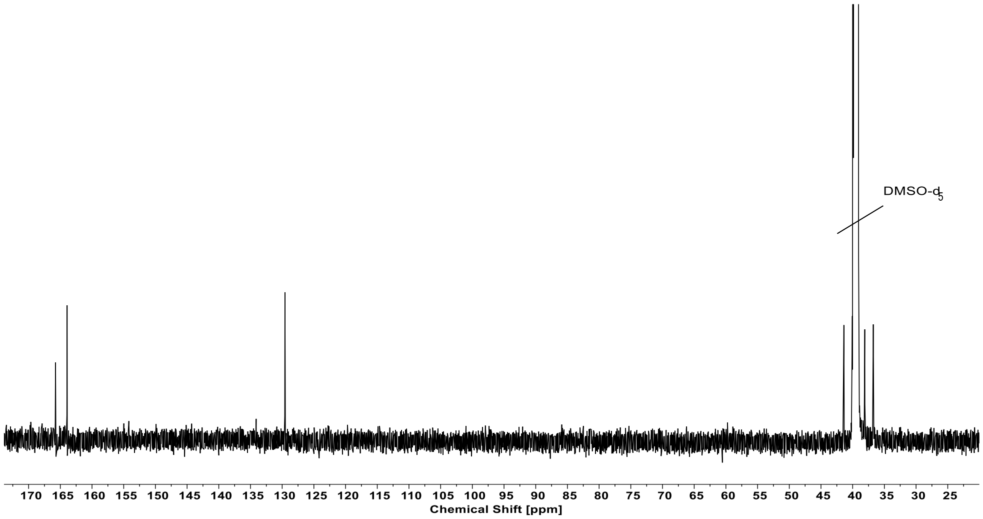

*Fig. S17: ^{13}C-NMR spectrum (150 MHz, $CDCl_3$, 297.9 K) of N,N'-(disulfanediylbis(ethan-2,1-diyl))bis(2-(3,4-dibromo-2,5-dioxo-2,5-dihydro-1H-pyrrol-1-yl)acetamide (rebridging agent) (**6**).*

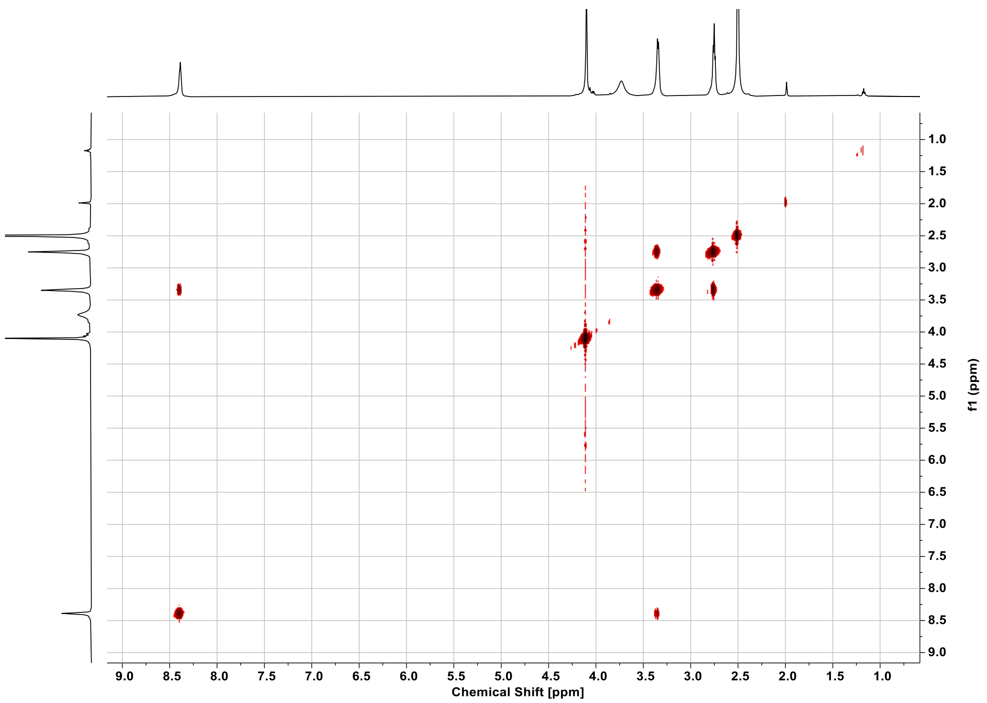

*Fig. S18: COSY-NMR spectrum (600 MHz, DMSO-d$_6$, 297.9 K) of N,N'-(disulfanediylbis(ethan-2,1-diyl))bis(2-(3,4-dibromo-2,5-dioxo-2,5-dihydro-1H-pyrrol-1-yl)acetamide (rebridging agent) (**6**).*

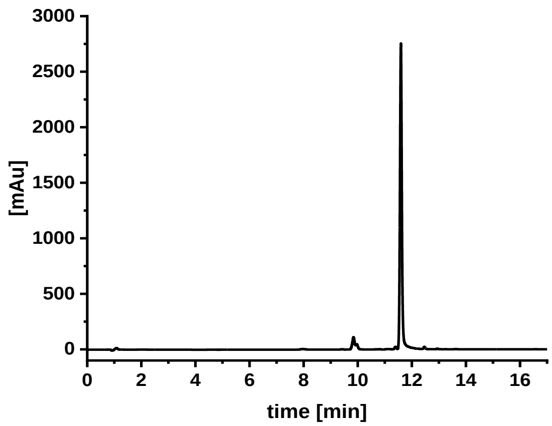

*Fig. S19: RP-HPLC chromatogram of N,N'-(disulfanediylbis(ethan-2,1-diyl))bis(2-(3,4-dibromo-2,5-dioxo-2,5-dihydro-1H-pyrrol-1-yl)acetamide (rebridging agent) (**6**) (gradient from 5 to 95vol.% acetonitrile/water with 0.1vol.% formic acid, run time: 17 min).*

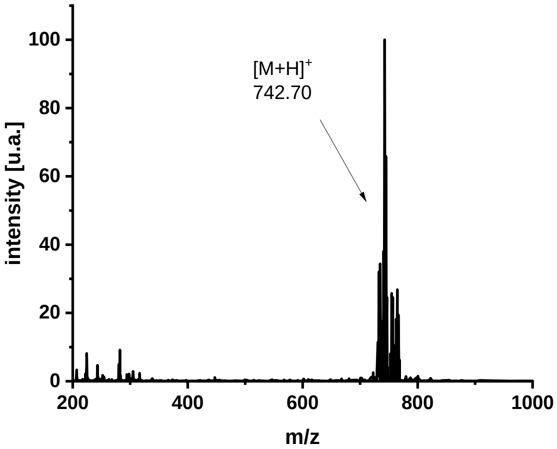

*Fig. S20: ESI mass spectrum of N,N'-(disulfanediylbis(ethan-2,1-diyl))bis(2-(3,4-dibromo-2,5-dioxo-2,5-dihydro-1H-pyrrol-1-yl)acetamide (rebridging agent) (**6**) at t = 11.86 min (gradient from 5 to 95 vol.% acetonitrile/water with 0.1 vol.% formic acid, run time: 17 min).*

Acquisition Parameter

Source Type	ESI	Ion Polarity	Positive	Set Nebulizer	0.3 Bar
Focus	Not active	Set Capillary	4000 V	Set Dry Heater	180 °C
Scan Begin	50 m/z	Set End Plate Offset	-500 V	Set Dry Gas	4.0 l/min
Scan End	1500 m/z	Set Collision Cell RF	600.0 Vpp	Set Divert Valve	Source

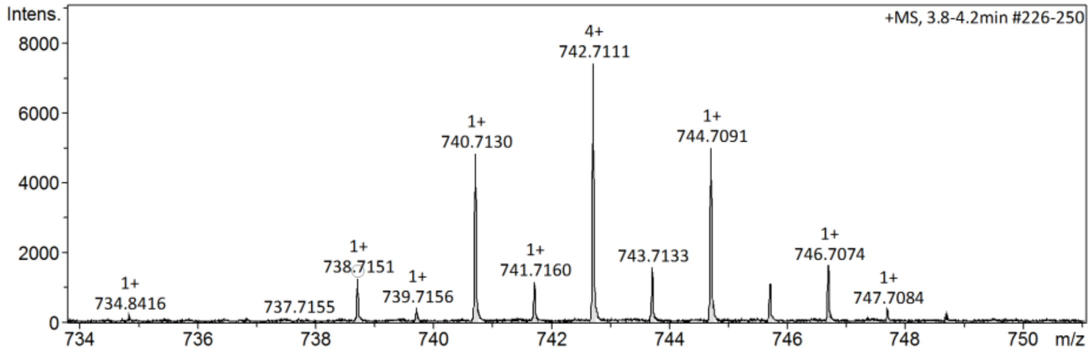

*Fig. S21: HR-ESI-MS spectrum of N,N'-(disulfanediylbis(ethan-2,1-diyl))bis(2-(3,4-dibromo-2,5-dioxo-2,5-dihydro-1H-pyrrol-1-yl)acetamide (rebridging agent) (**6**).*

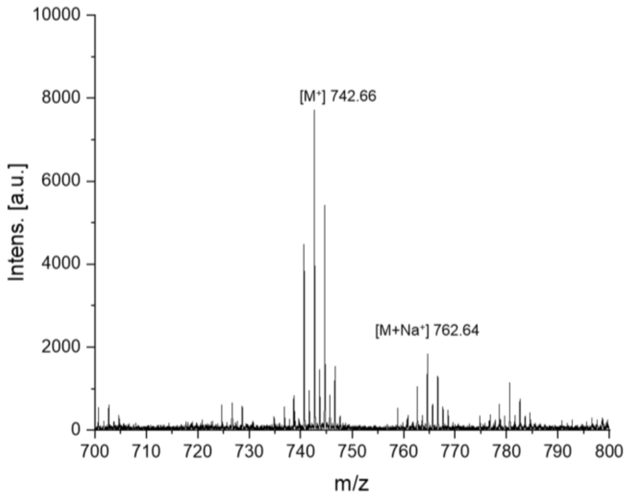

*Fig. S22: MALDI-TOF-MS of N,N'-(disulfanediylbis(ethan-2,1-diyl))bis(2-(3,4-dibromo-2,5-dioxo-2,5-dihydro-1H-pyrrol-1-yl)acetamide (rebridging agent) (**6**).*

2.3 Conjugation of the rebridging agent to precursor conjugates 1 and 4

Based on the analytical methods currently available to our group and collaboration partners, at this time, we cannot yet quantify the conjugation efficiency and determine how much BSA might remain unfunctionalized.

S4 – Synthesis of BSA-pHEAA conjugated with rebridging agent (BSA-pHEAA-r.b.)

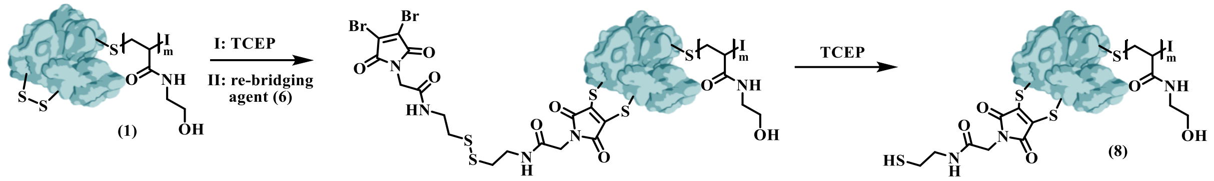

The synthesis was carried out as described in the standard protocol and a yellowish solid was obtained.

CD: area 215 nm – 245 nm: 219 nm, -25.93 mdeg, area 198 nm – 215 nm: 208.0 nm, -29.08 mdeg.

UV/Vis: maximum 382 nm.

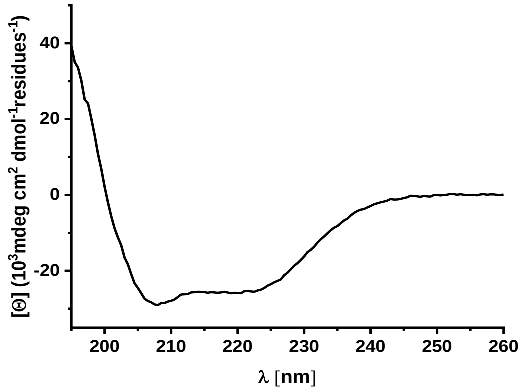

*Fig. S23: CD spectrum of BSA-p(HEAA)-rebridging agent (**S4**).*

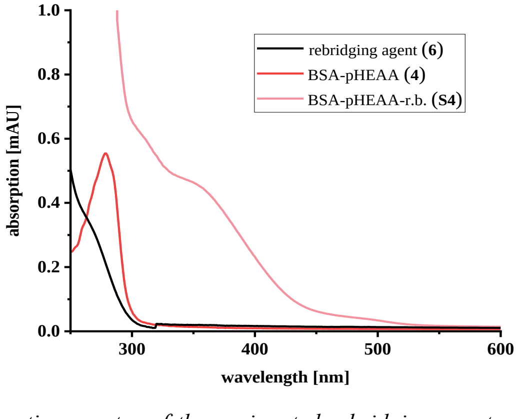

*Fig. S24: Presentation of the absorption spectra of the conjugated rebridging agent on BSA-pHEAA (**4**) and BSA-pHEAA)-r.b. (**S4**). The maximum absorption after four minutes is at 382 nm.*

S5 – Synthesis of BSA-pNIPAM conjugated with rebridging agent (BSA-pNIPAM-r.b.)

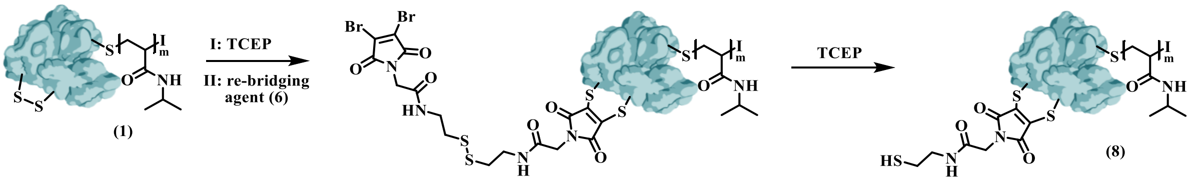

The synthesis was carried out as described in the standard protocol and a yellowish solid was obtained.

CD: area 215 nm – 245 nm: 220 nm, -26.59 mdeg, area 198 nm – 215 nm: 208.0 nm, -29.21 mdeg.

UV/Vis: maximum 382 nm.

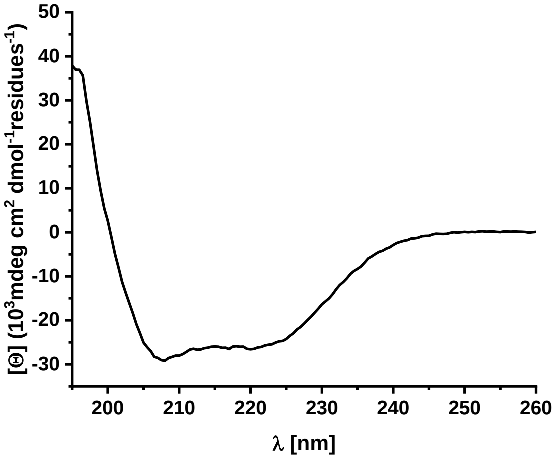

*Fig. S25: CD spectrum of BSA-pNIPAM-rebridging agent (**S5**).*

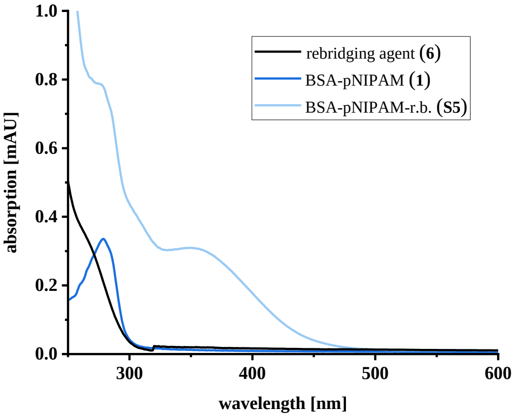

*Fig. S26: Presentation of the absorption spectra of the conjugated rebridging agent on BSA-pNIPAM (**1**) and BSA-pNIPAM-r.b. (**S5**). The maximum absorption after four minutes is at 382 nm.*

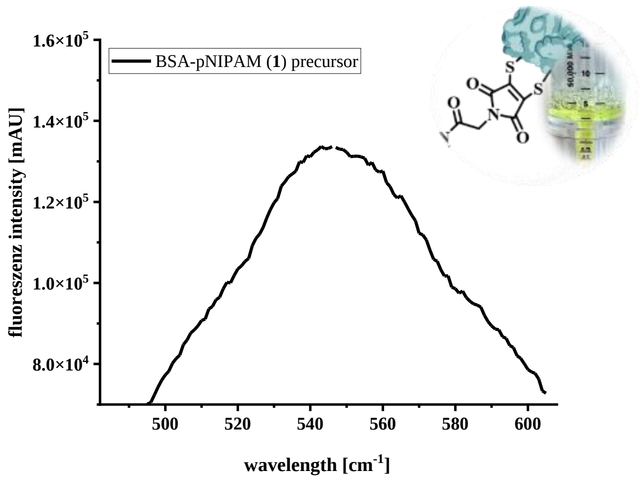

*Fig. S27: Exemplary presentation of the fluorescence spectrum of the conjugated rebridging agent on BSA-pNIPAM (**1**) precursor*

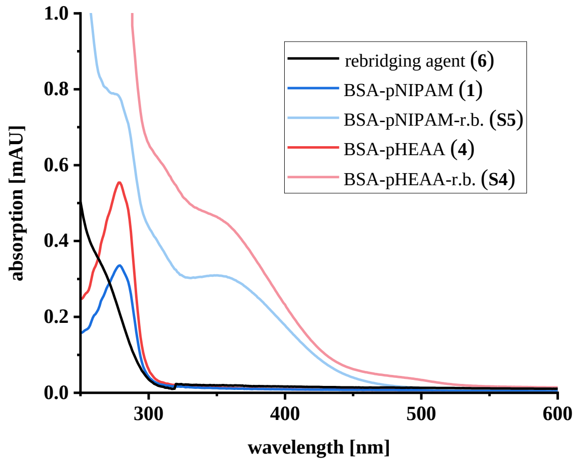

*Fig. S28: Comparison of the absorption spectra of the conjugated rebridging agent on BSA-pNIPAM (**1**) and BSA-pHEAA (**4**). The maximum absorption after four minutes is at 382 nm.*

7 – Synthesis of pHEAA-BSA-pNIPAM

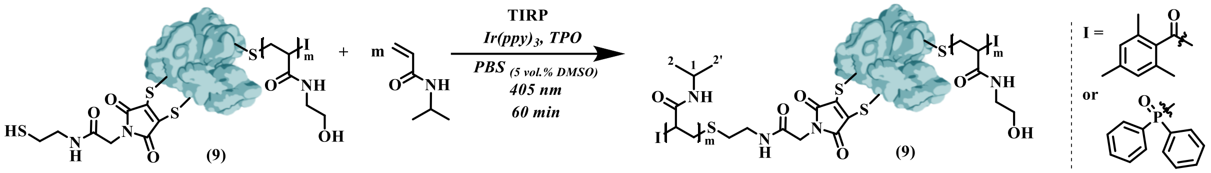

The synthesis was carried out as described in the standard protocol and a yellowish solid was obtained.

^{1}H-NMR (600 MHz, D_2O, 297.9 K) δ (ppm) 8.43 – 6.25 (m, H_{BSA}), 4.53 – 0.00 (m, H_{BSA}, $H_{HEAA\text{-side chain}}$, $H_{NIPAM\text{-side chain}}$ $H_{HEAA\text{-backbone}}$, $H_{NIPAM\text{-backbone}}$, overlap with water peak), 3.78 – 3.19 (m, $H_{HEAA\text{-Seitenkette}}$), 3.93 ($s_{br}$, *H1*), 1.18 ($s_{br}$, *H2, H´2*).

AF4-LS (1 mM PBS, pH = 7.4, 298,15 K, monomer fraction): Mn = 71.3 kDa, Mw = 71.3 kDa, Đ = 1.00, mol fraction: 58.4% compared to the monomer fraction of BSA-p(HEAA) (***4***) with 66.1 kDa, the re-bridged derivative ***9*** is about 5.2 kDa larger than the mono functional derivate ***4***.

CD: area 215 nm – 245 nm: 219.5 nm, -27.05 mdeg, area 198 nm – 215 nm: 208.0 nm, -30.29 mdeg.

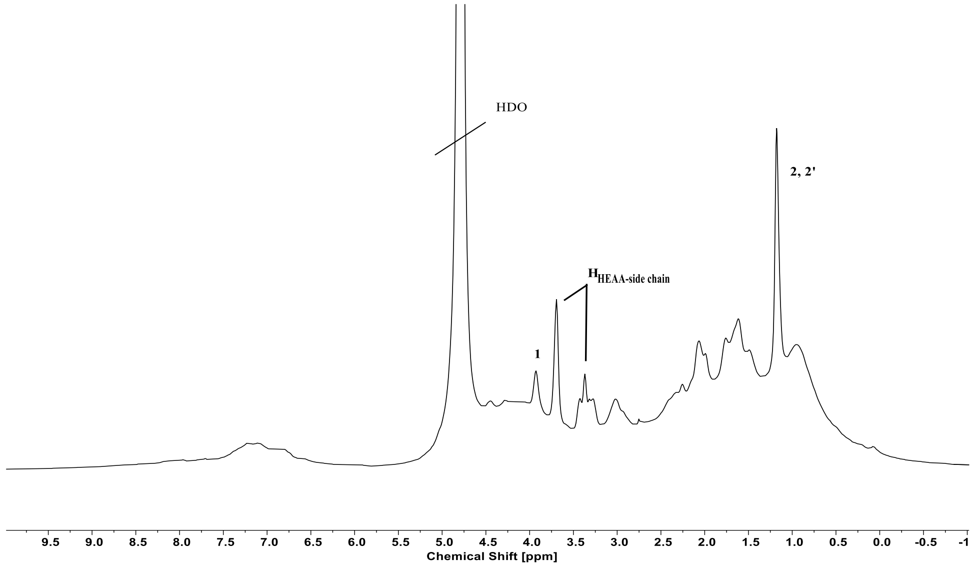

*Fig. S29: ^{1}H-NMR spectrum (600 MHz, DMSO-d$_6$, 297.9 K) of Synthesis of BSA-pHEAA-r.b.-pNIPAM (**7**).*

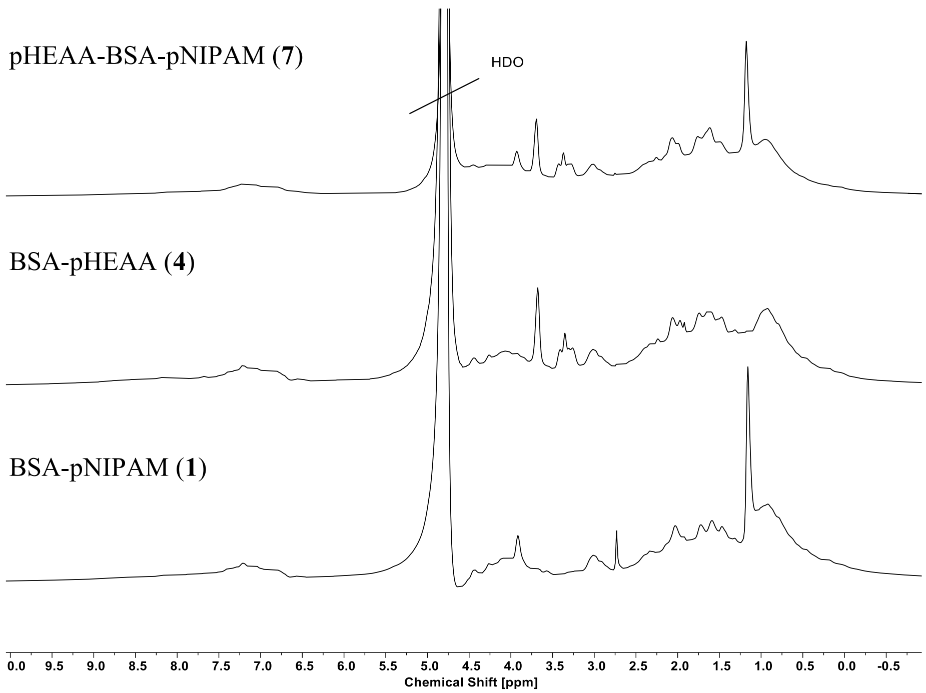

*Fig. S30: Comparison of ^{1}H-NMR spectrum (600 MHz, DMSO-d_6, 297.9 K) of pHEAA-BSA-pNIPAM (**7**), BSA-pHEAA (**4**), BSA-pNIPAM (**1**).*

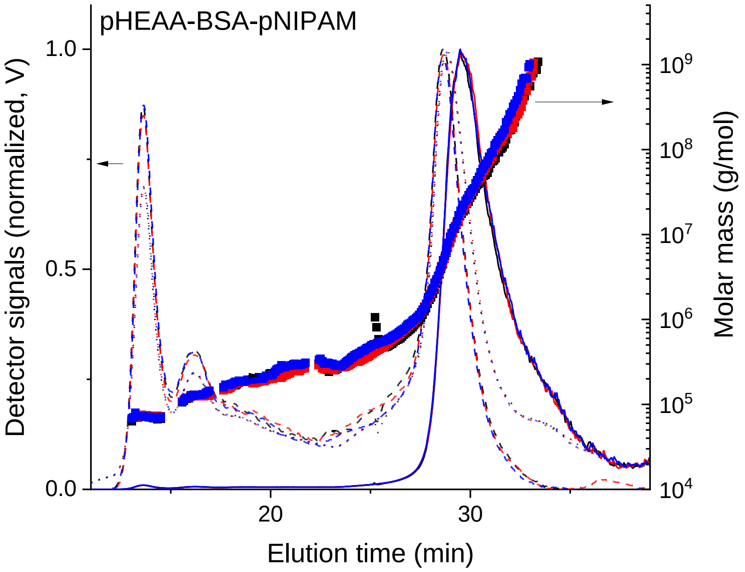

*Fig. S31: Triplicate measurements and presentation of fractograms of pHEAA-BSA-pNIPAM (**7**) opt. separation, RI (dashed line), UV (dotted line) and LS signal (solid line), molar masses (symbols) vs. elution time.*

Table S7: Results of the triplicate AF4-LS measurements.

**Measurement**	**M_n*1** (kg/mol)	**M_w*1** (kg/mol)	**Đ** (M_w/M_n)	**R_g** (nm)	**R_h** (nm)
**1.** (50 µl)	205	10,500	51.4	89.6	91.7
**2.** (50 µl)	209	11,500	55.4	92.0	88.8
**3.** (50 µl)	207	13,200	63.8	93.9	87.1
**average (whole peak)**	**207**	**11,800**	**56.8**	**91.8**	**89.2**

*1 dn/dc = 0.172 ml/g, average of all components

Table S8: Analysis of the individual fractions of AF4-LS. The values shown are average values from the triplicate measurements.

**fraction**	**M_n*1** (kg/mol)	**M_w*1** (kg/mol)	**Đ** (M_w/M_n)	**mass fraction** (%)	**mol fraction** (%)
1 - monomer	71.3	71.3	1.00	19.8	58.4
2 – dimer	125	125	1.00	12.0	20.8
3- trimer / multimer	211	212	1.01	12.1	12.1
4 - aggregates	1,280	23,800	18.5	56.1	8.7

**1 dn/dc = 0.172 ml/g, average of all components*

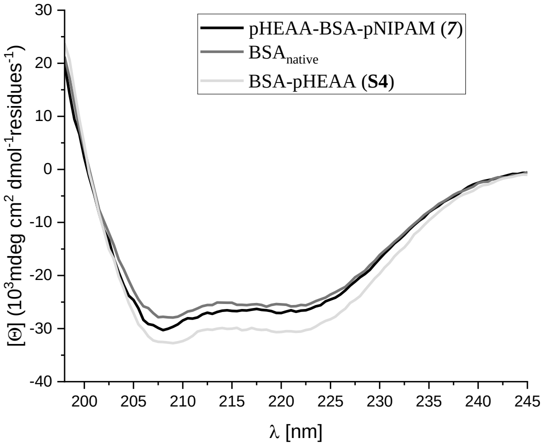

*Fig. S32: Comparison of the CD spectra of the BSA_{native}, BSA-pHEAA (**4**), BSA-pHEAA-r.-b. (**S4**) und pHEAA-BSA-pNIPAM (**7**).*

8 – Synthesis of pNIPAM-BSA-pMan

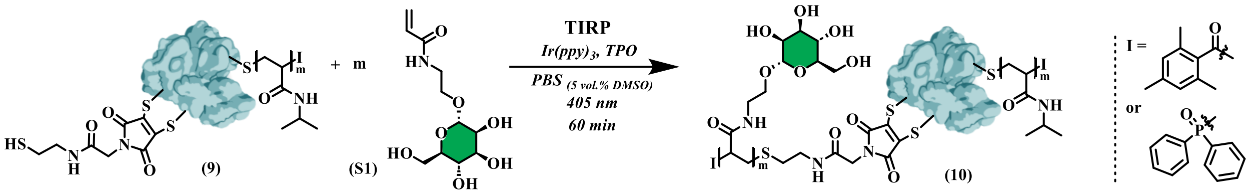

The synthesis was carried out as described in the standard protocol and a yellowish solid was obtained.

^{1}H-NMR (600 MHz, D_2O, 297.9 K) δ (ppm) 8.37 – 6.43 (m, H_{BSA}), 4.47 – 0.00 (m, H_{BSA}, $H_{NIPAM\text{-}side\ chain}$, $H_{Man\text{-}side\ chain}$, $H_{NIPAM\text{-}backbone}$, $H_{Man\text{-}backbone}$, overlap with water peak), 4.06 – 3.55 (s_{br}, $H_{Man\text{-}side\ chain}$), 3.92 ($s_{br}$, *H1*), 1.17 ($s_{br}$, *H2, H´2*).

AF4-LS (1 mM PBS, pH = 7.4, 298,15 K, monomer fraction): Mn = 73.4 kDa, Mw = 73.4 kDa, Đ = 1.00, mol fraction: 57.5% compared to the monomer fraction of BSA-pNIPAM (**1**) with 70.5 kDa, the re-bridged derivative **8** is about 2.9 kDa larger than the mono functional derivate ***1***.

CD: area 215 nm – 245 nm: 220.0 nm, -27.47 mdeg, area 198 nm – 215 nm: 209.0 nm, -30.24 mdeg.

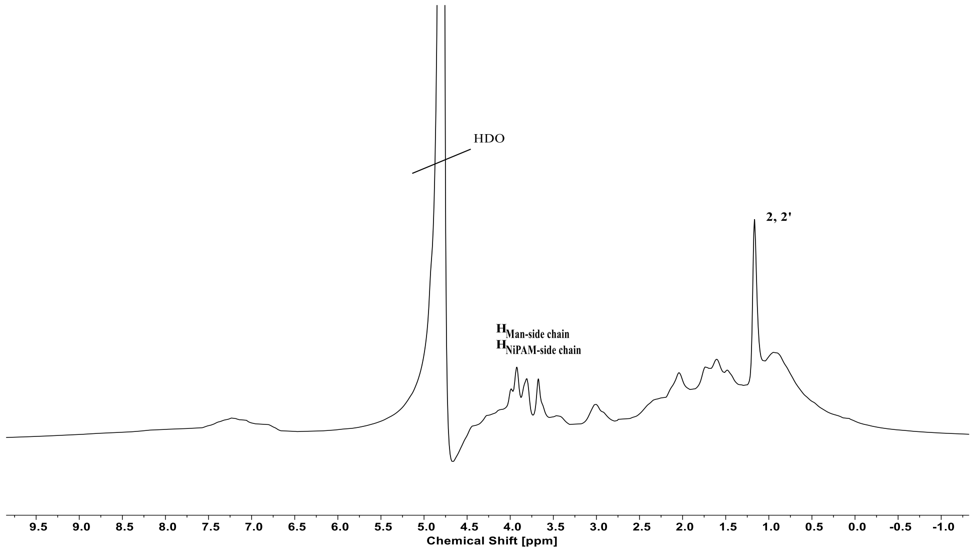

*Fig. S33: ^{1}H-NMR spectrum (600 MHz, DMSO-d$_6$, 297.9 K) of Synthesis of pNIPAM-BSA-pMan (**8**).*

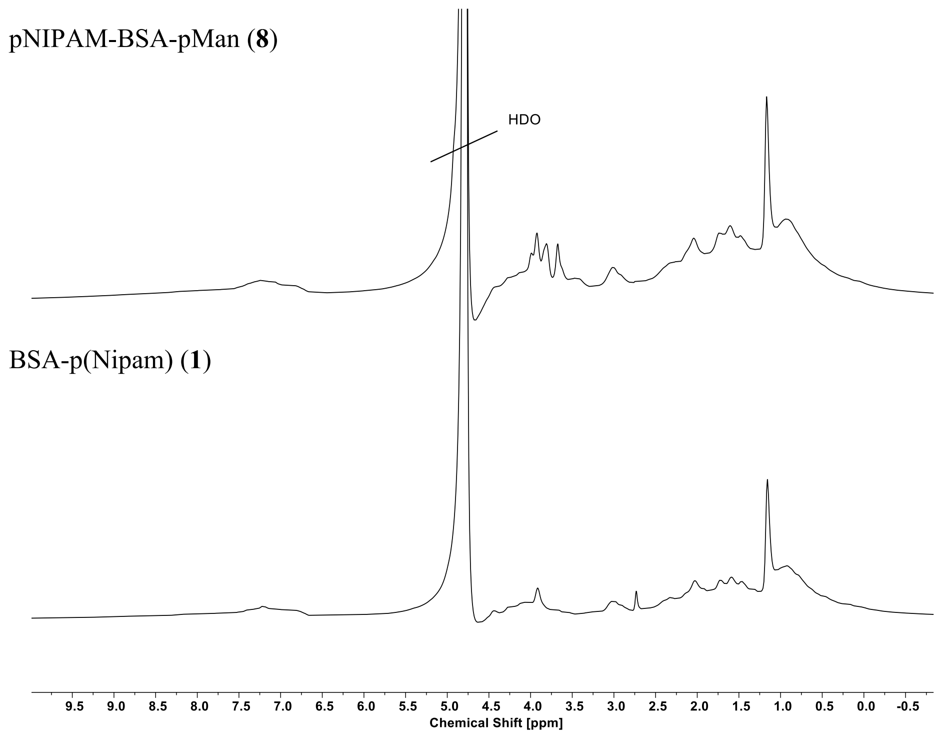

*Fig. S34: Comparison of ^{1}H-NMR spectrum (600 MHz, DMSO-d$_6$, 297.9 K) of pNIPAM-BSA-pMan (**8**), BSA-pNIPAM (**1**).*

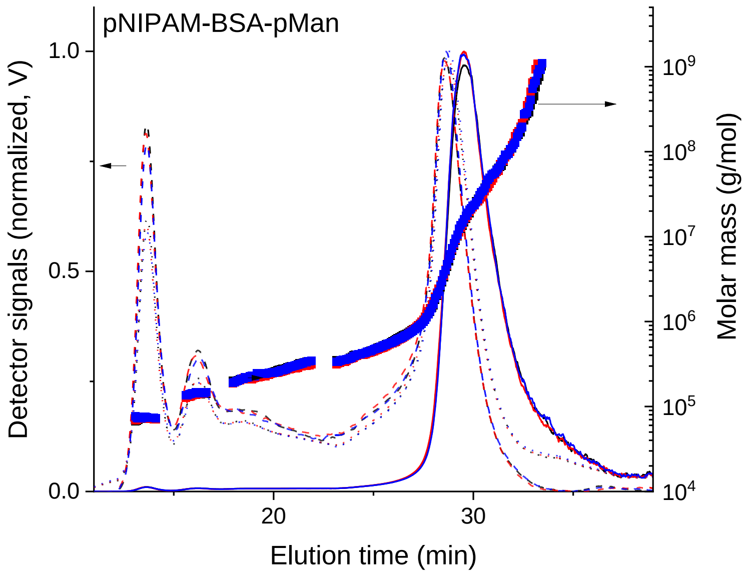

*Fig. S35: Triplicate measurements and presentation of fractograms of pNIPAM-BSA-pMan (**8**) opt. separation, RI (dashed line), UV (dotted line) and LS signal (solid line), molar masses (symbols) vs. elution time.RI, UV and LS signal, molar masses vs. elution time.*

Table S9: Results of the triplicate AF4-LS measurements.

measurement	M_n *1 (kg/mol)	M_w *1 (kg/mol)	Đ (M_w/M_n)	R_g (nm)	R_h (nm)
**1.** (50 µl)	232	7,930	34.2	81.3	74.1
**2.** (50 µl)	229	7,740	33.8	75.4	72.1
**3.** (50 µl)	244	8,960	36.8	80.0	79.1
**Average (whole Peak)**	235	**8,210**	**34.9**	**78.9**	**75.1**

*1 dn/dc = 0.172 ml/g, average of all components

Table S10: Analysis of the individual fractions of AF4-LS. The values shown are average values from the triplicate measurements.

fraction	M_n *1 (kg/mol)	M_w *1 (kg/mol)	Đ (M_w/M_n)	mass fraction (%)	mol fraction (%)
1 - monomer	73.4	73.4	1.00	17.9	57.5
2 – dimer	141	141	1.00	11.1	18.7
3- trimer / multimer	264	271	1.03	13.3	13.9
4 - aggregates	1,310	14,300	10.9	57.7	9.9

*1 dn/dc = 0.172 ml/g, average of all components

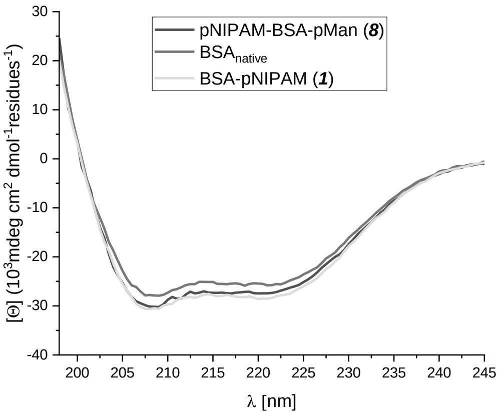

*Fig. S36: Comparison of the CD spectra of the BSA_{native}, BSA-pNIPAM (**1**), BSA-pNIPAM-r.b. (**S5**) und pNIPAM-BSA-pMan (**8**).*

9 – Synthesis of pNIPAM-BSA-pGal

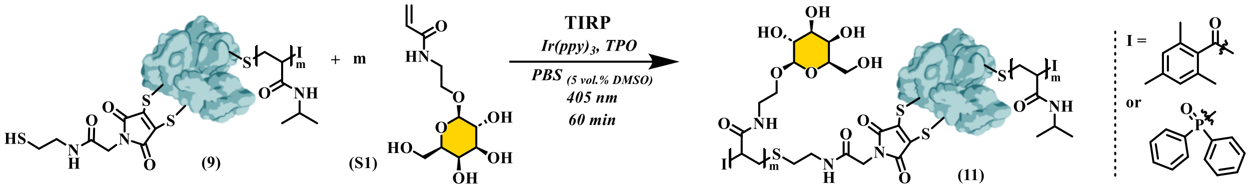

The synthesis was carried out as described in the standard protocol and a yellowish solid was obtained.

^{1}H-NMR (600 MHz, D_2O, 297.9 K) δ (ppm) 8.36 – 6.45 (m, H_{BSA}), 4507 – 0.00 (m, H_{BSA}, $H_{NIPAM\text{-}side\ chain}$, $H_{Gal\text{-}side\ chain}$, $H_{NIPAM\text{-}backbone}$, $H_{Gal\text{-}backbone}$, overlap with water peak), 4.46 (s_{br}, $H_{anomer\ of\ galactose}$) 4.51 – 3.33 ($s_{br}$, $H_{Gal\text{-}side\ chain}$), 1.17 ($s_{br}$, *H2, H´2*).

AF4-LS (1 mM PBS, pH = 7.4, 298,15 K, monomer fraction): Mn = 74.8 kDa, Mw = 74.8 kDa, Đ = 1.00, mol fraction: 54.6% compared to the monomer fraction of BSA-pNIPAM (***1***) with 70.5 kDa, the re-bridged derivative **9** is about 4.3 kDa larger than the mono functional derivate ***1***.

CD: area 215 nm – 245 nm: 219.0 nm, -27.97 mdeg, area 198 nm – 215 nm: 208.5 nm, -30.53 mdeg.

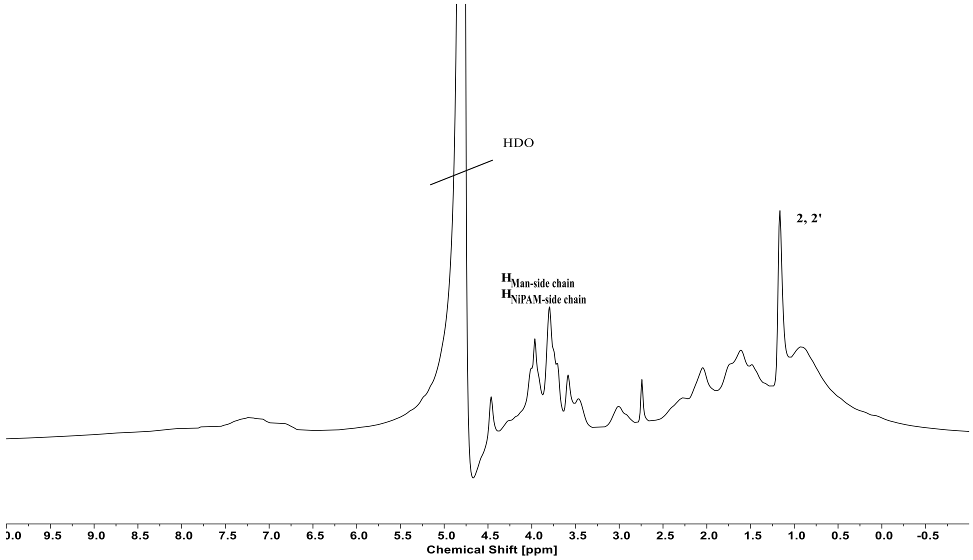

*Fig. S37: ^{1}H-NMR spectrum (600 MHz, DMSO-d_6, 297.9 K) of Synthesis of pNIPAM-BSA-pGal (**9**).*

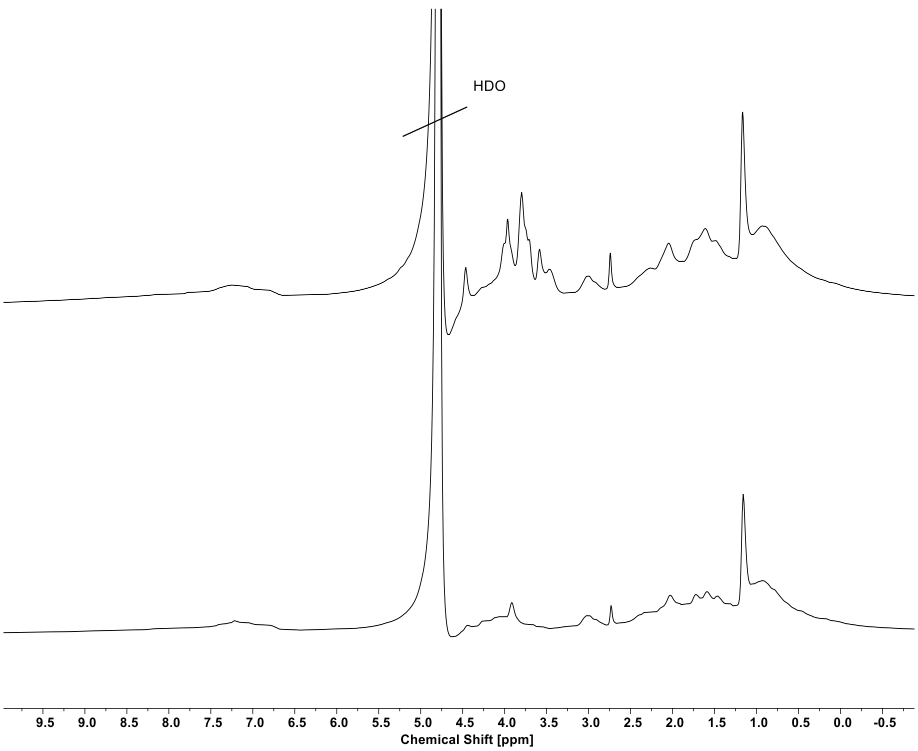

*Fig. S38: Comparison of ^{1}H-NMR spectrum (600 MHz, DMSO-d_6, 297.9 K) of pNIPAM-BSA-pGal (**9**), BSA-pNIPAM (**1**).*

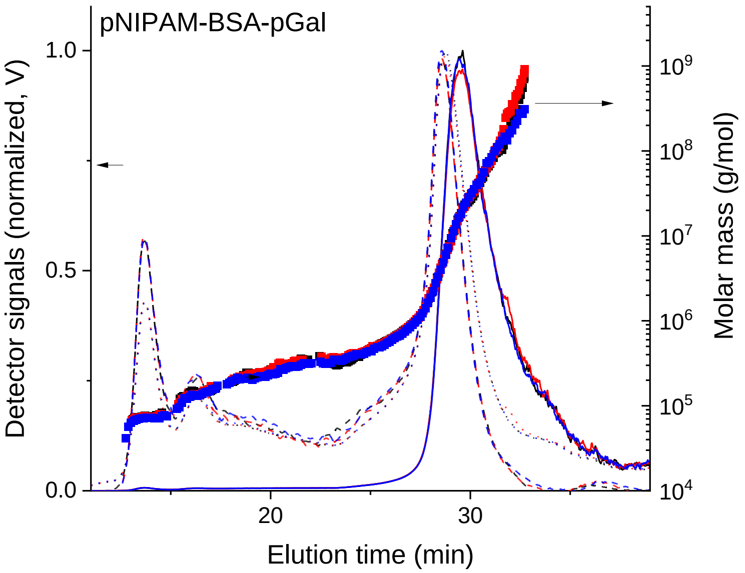

*Fig. S39: Triplicate measurements and presentation of fractograms of pNIPAM-BSA-pGal (**9**) opt. separation, RI (dashed line), UV (dotted line) and LS signal (solid line), molar masses (symbols) vs. elution time.*

Table S11: Results of the triplicate AF4-LS measurements.

**measurement**	$\mathbf{M_n}^{*1}$ (kg/mol)	$\mathbf{M_w}^{*1}$ (kg/mol)	**Đ** (M_w/M_n)	$\mathbf{R_g}$ (nm)	$\mathbf{R_h}$ (nm)
**1.** (50 µl)	272	9,000	33.1	108	78.7
**2.** (50 µl)	266	12,500	47.0	131	78.5
**3.** (50 µl)	256	10,100	39.5	119	86.8
**average (whole peak)**	265	**10,500**	**39.9**	**117**	**81.3**

*1 dn/dc = 0.172 ml/g, average of all components

Table S12: Analysis of the individual fractions of AF4-LS. The values shown are average values from the triplicate measurements.

**fraction**	$\mathbf{M_n}^{*1}$ (kg/mol)	$\mathbf{M_w}^{*1}$ (kg/mol)	**Đ** (M_w/M_n)	**mass fraction** (%)	**mol fraction** (%)
1 - monomer	74.8	74.8	1.00	15.3	54.6
2 – dimer	143	144	1.01	11.0	18.1
3- trimer / multimer	252	253	1.01	12.7	14.6
4 - aggregates	1,260	14,500	11.5	61.0	12.7

*1 dn/dc = 0.172 ml/g, average of all components

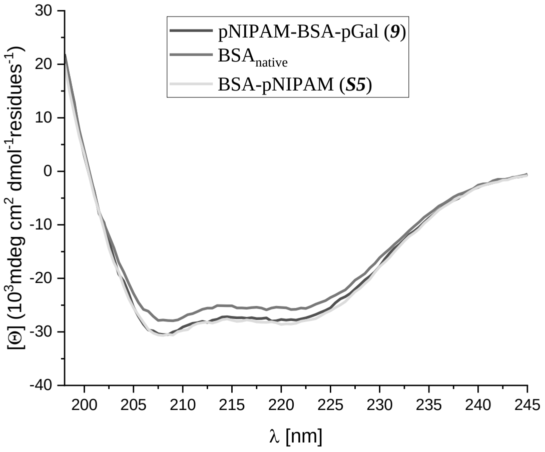

*Fig. S40: Comparison of the CD spectra of the BSAnative, BSA-pNIPAM (**1**), BSA-pNIPAM-r.b. (**S5**) und pNIPAM-BSA-pGal (**9**).*

2.4 DLS Particle Experiments

Particle properties of BSA-pNIPAM samples 1, 2 and 3 by using a 2 min temperature ramp (1°C/min)

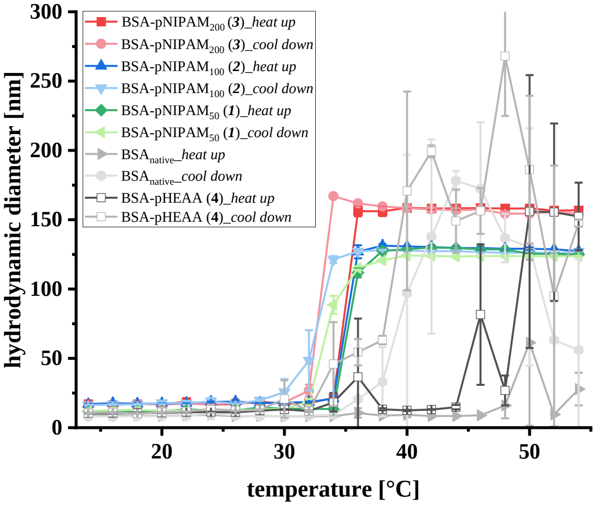

*Fig. S41: Hydrodynamic diameter profiles of temperature-dependent particle formation and dissociation of the BSA-pNIPAM conjugates **1-4** and native BSA at a concentration of 0.5 mg/mL in PBS buffer with a heating rate of 1 °C/min.*

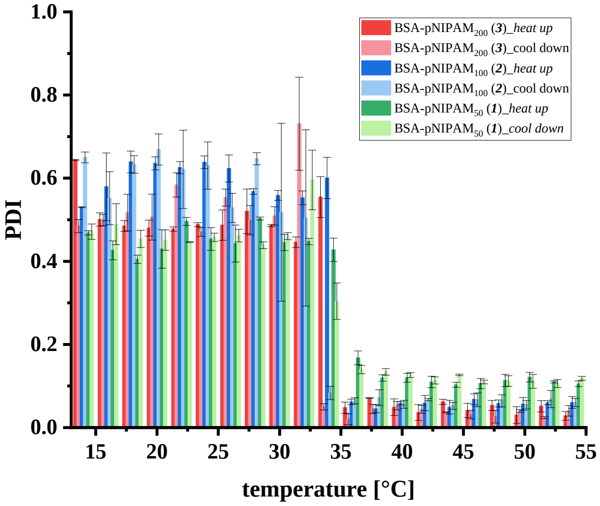

*Fig. S42: Polydispersities of the DLS measurements (PDI) of temperature-dependent particle formation and dissociation of the BSA-pNIPAM conjugates **1**, **2** and **3** at a concentration of 0.5 mg/mL in PBS buffer with a heating rate of 1 °C/min.*

Particle properties of BSA-pNIPAM samples 1, 2 and 3 by using a 10 min temperature ramp (0.2 °C/min)

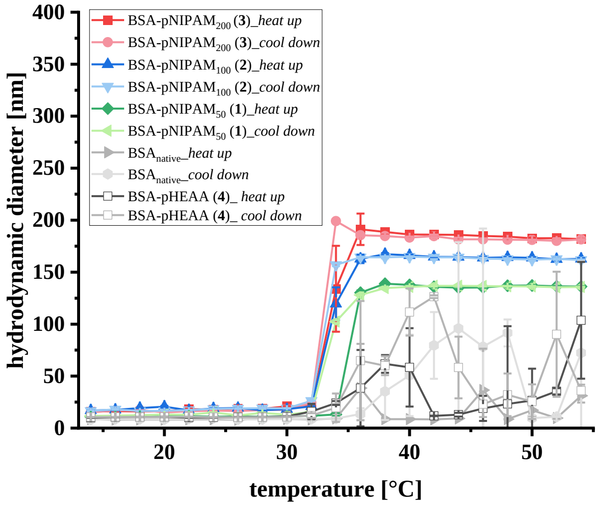

*Fig. S43: Hydrodynamic diameter profiles of temperature-dependent particle formation and dissociation of the BSA-pNIPAM conjugates **1** - **4** and native BSA at a concentration of 0.5 mg/mL in PBS buffer with a heating rate of 0.2 °C/min.*

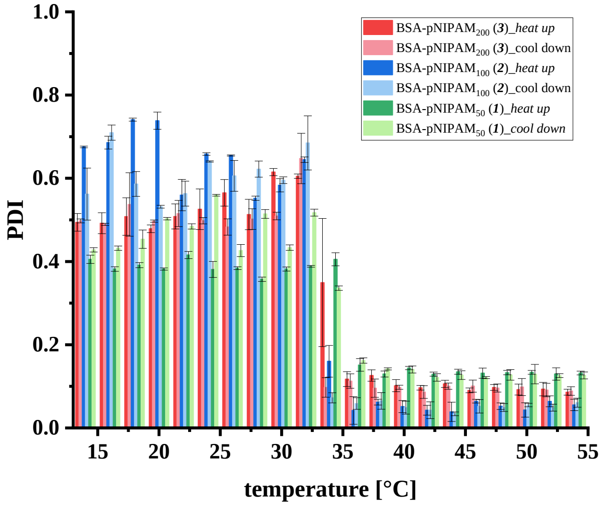

*Fig. S44: Polydispersities of the DLS measurements (PDI) of temperature-dependent particle formation and dissociation of the BSA-pNIPAM conjugates **1**, **2** and **3** at a concentration of 0.5 mg/mL in PBS buffer with a heating rate of 0.2 °C/min.*

Particle properties of BSA-pNIPAM samples 1, 2 and 3 by using a 20 min temperature ramp (0.1 °C/min)

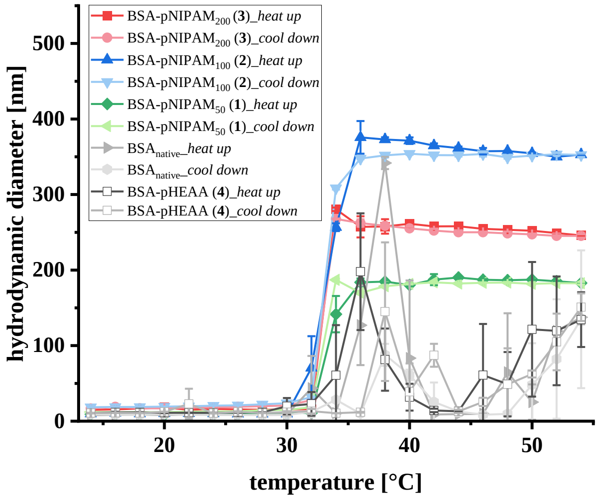

*Fig. S45: Hydrodynamic diameter profiles of temperature-dependent particle formation and dissociation of the BSA-pNIPAM conjugates **1-4** and native BSA at a concentration of 0.5 mg/mL in PBS buffer with a heating rate of 0.1 °C/min.*

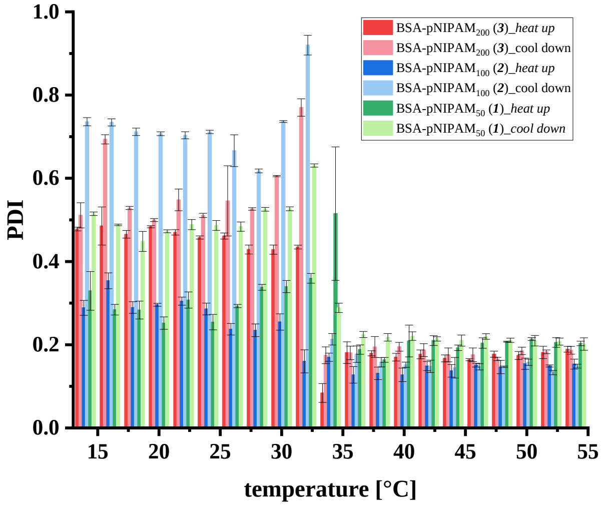

*Fig. S46: Polydispersities of the DLS measurements (PDI) of temperature-dependent particle formation and dissociation of the BSA-pNIPAM conjugates **1**, **2** and **3** at a concentration of 0.5 mg/mL in PBS buffer with a heating rate of 0.2 °C/min.*

Particle properties of BSA-pNIPAM samples 1, 2 and 3 depending on the heating rate

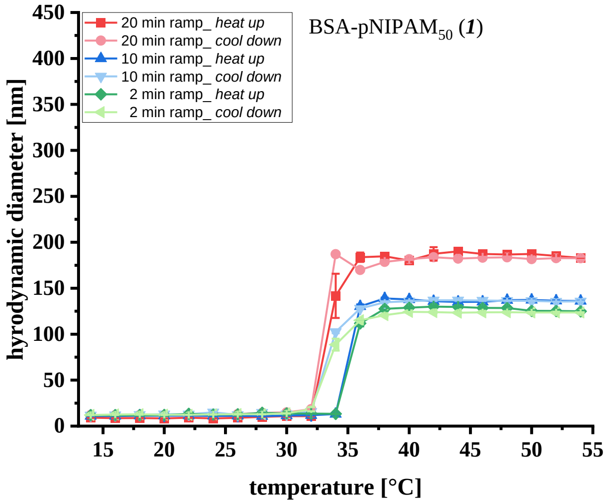

*Fig. S47: Hydrodynamic diameters profile of temperature-dependent particle formation and dissociation of the BSA-pNIPAM conjugates **1** at a concentration of 0.5 mg/mL in PBS buffer with different heating rates from 1°C/min, 0.2°C/min and 0.1 °C/min.*

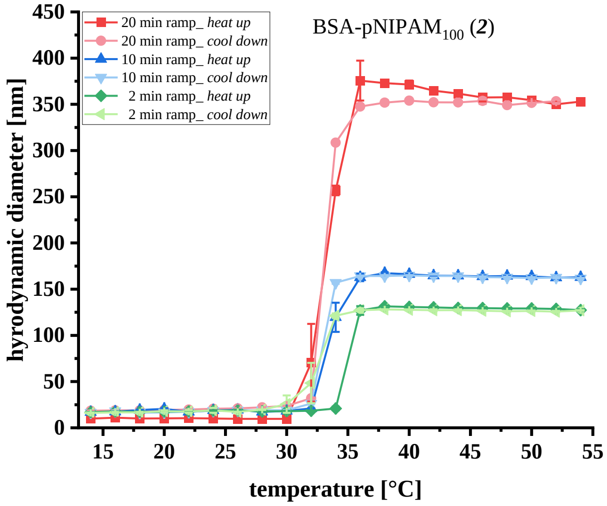

*Fig. S48: Hydrodynamic diameters profile of temperature-dependent particle formation and dissociation of the BSA-pNIPAM conjugates **2** at a concentration of 0.5 mg/mL in PBS buffer with different heating rates from 1°C/min, 0.2°C/min and 0.1 °C/min.*

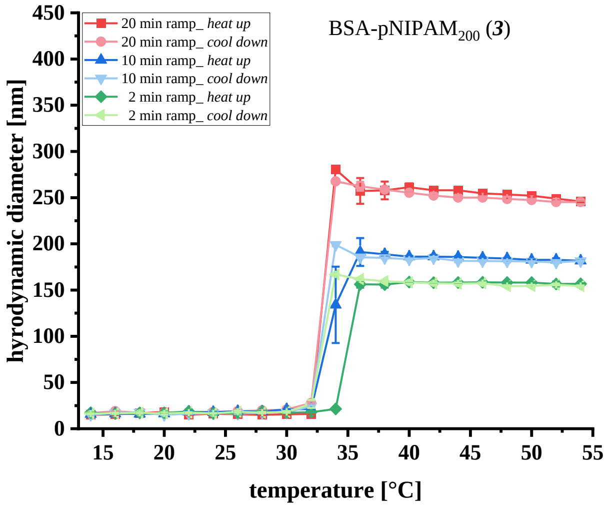

*Fig. S49: Hydrodynamic diameters profile of temperature-dependent particle formation and dissociation of the BSA-pNIPAM conjugates **3** at a concentration of 0.5 mg/mL in PBS buffer with different heating rates from 1°C/min, 0.2°C/min and 0.1 °C/min.*

Particle properties of BSA-pNIPAM samples 1, 2 and 3 depending on the concentration

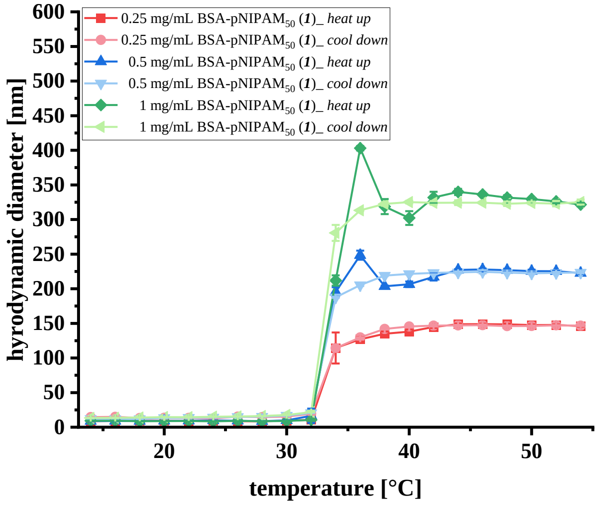

*Fig. SS50: Hydrodynamic diameters profile of temperature-dependent particle formation and dissociation of the BSA-pNIPAM conjugates **1** at different concentration of 0.25 mg/mL, 0.5 mg/mL and 1 mg/mL in PBS buffer with a heating rate of 0.1 °C/min.*

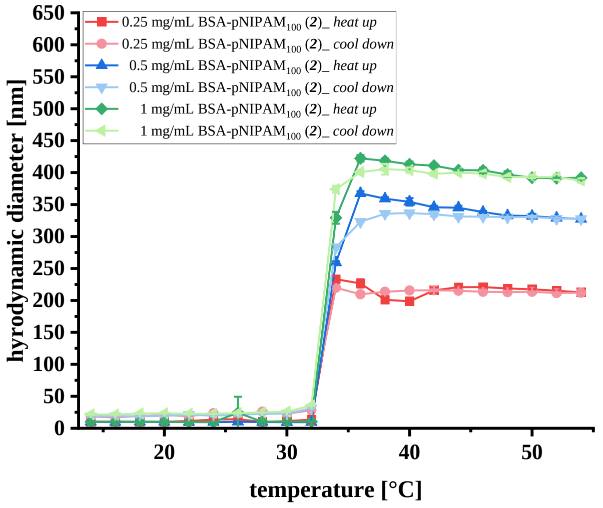

*Fig. S51: Hydrodynamic diameters profile of temperature-dependent particle formation and dissociation of the BSA-pNIPAM conjugates **2** at different concentration of 0.25 mg/mL, 0.5 mg/mL and 1 mg/mL in PBS buffer with a heating rate of 0.1 °C/min.*

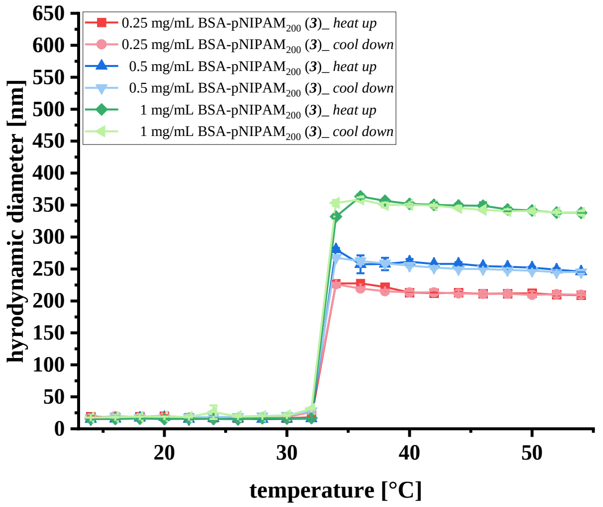

*Fig. S52: Hydrodynamic diameters profile of temperature-dependent particle formation and dissociation of the BSA-pNIPAM conjugates **3** at different concentration of 0.25 mg/mL, 0.5 mg/mL and 1 mg/mL in PBS buffer with a heating rate of 0.1 °C/min.*

2.5 Inhibition-Competition experiment of double switchable systems

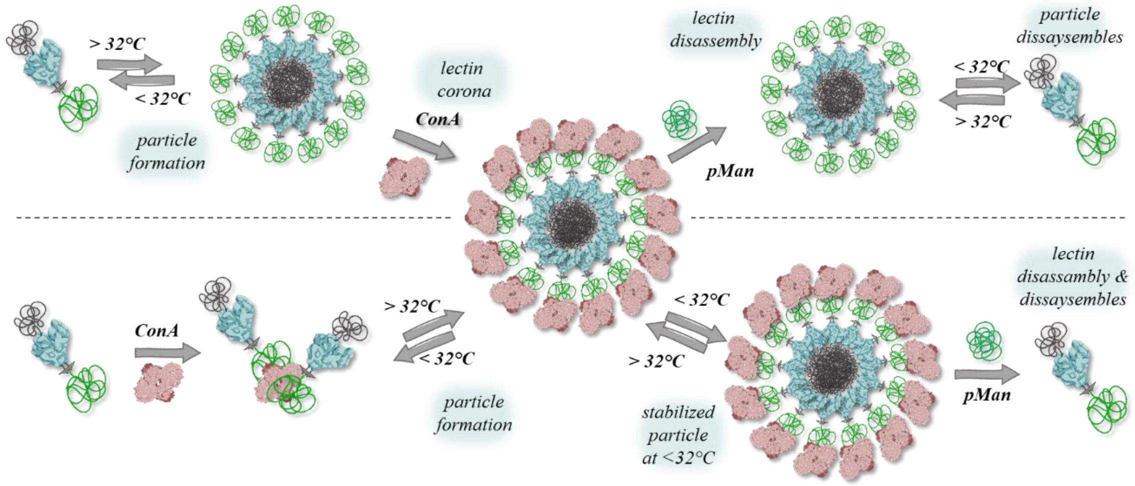

Experiments based on pNIPAM-BSA-pMan (8)

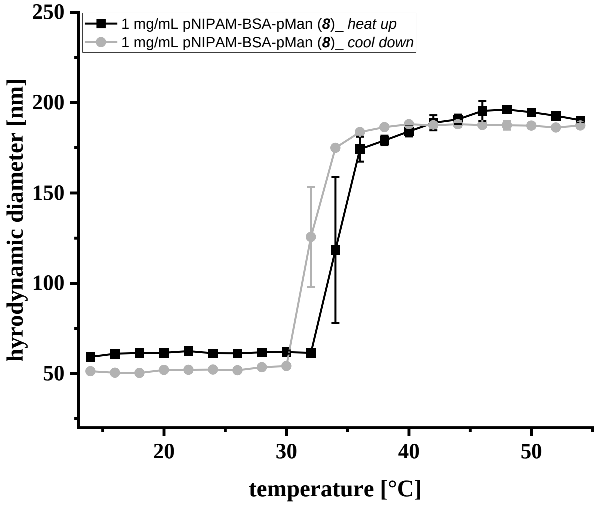

*Fig. S53: Hydrodynamic diameter profile of temperature-dependent particle formation and dissociation of the pNIPAM-BSA-pMan conjugates **8** at a concentration of 1 mg/mL in PBS buffer with a heating rate of 1 °C/min.*

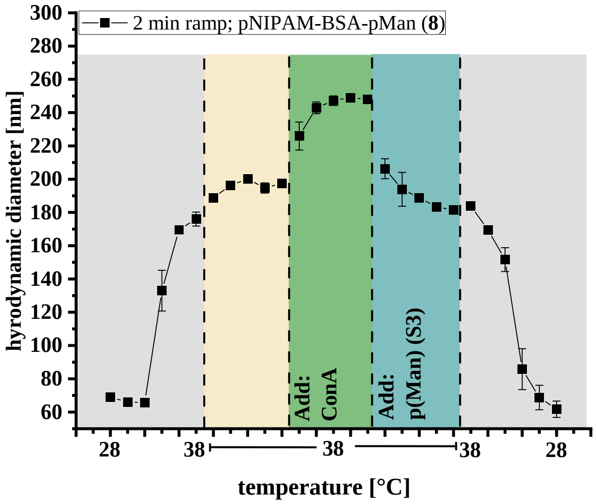

*Fig. S54: Hydrodynamic diameter profil of temperature-dependent particle formation and dissociation with the polymeric inhibitor pMan (**S3**) (15 mM in LBB) of the PNIPAM-BSA-pMan conjugates **8** at a concentration of 13.8 µM in LBB buffer with a heating rate of 1 °C/min.*

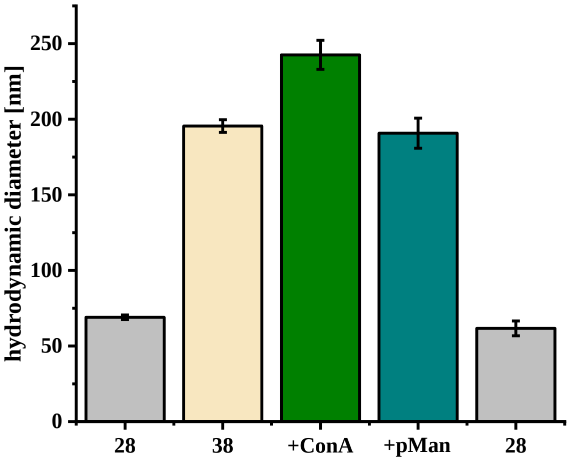

*Fig. S55: The mean values of the hydrodynamic diameter of temperature-dependent particle formation and dissociation from pNIPAM-BSA-pMan conjugates **8** at a concentration of 13.8 µM in LBB buffer with a heating rate of 1 °C/min and with the polymeric inhibitor pMan (**S3**) (15 mM in LBB).*

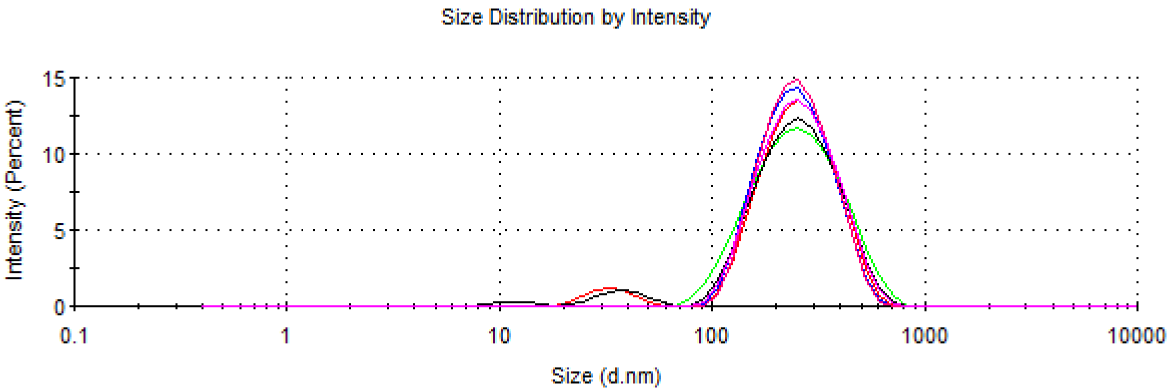

*Fig. S56: Hydrodynamic diameter distribution of pNIPAM-BSA-pMan conjugates **8** at a concentration of 13.8 µM in LBB buffer with a heating rate of 1 °C/min after particle formation (38°C).*

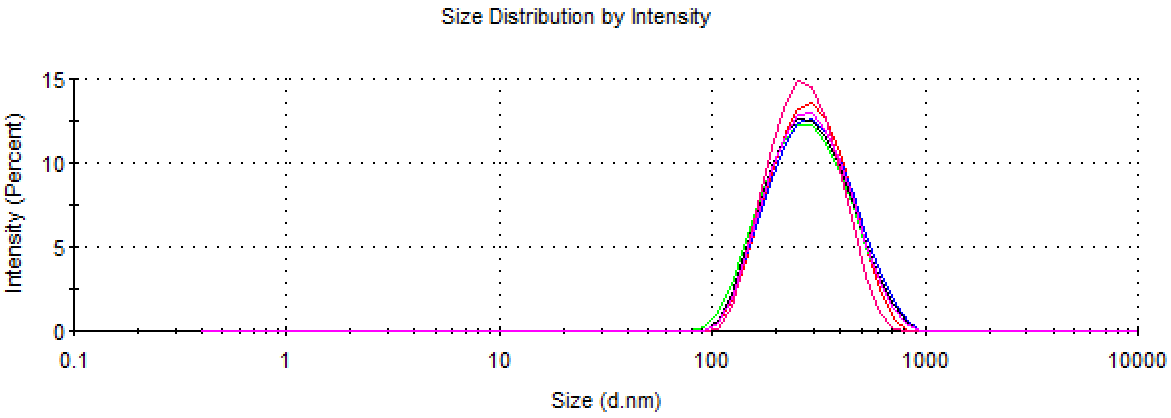

*Fig. S57: Hydrodynamic diameter distribution of pNIPAM-BSA-pMan conjugates **8** at a concentration of 13.8 µM in LBB buffer with a heating rate of 1 °C/min with lectin (ConA) corona at 38°C.*

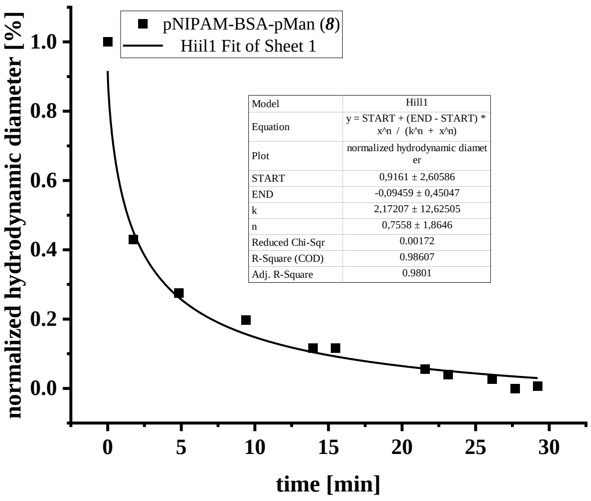

*Fig. S58: Kinetics of pNIPAM-BSA-pMan (**8**) inhibition after pMan addition (Hill 1 Fit).*

Experiments based on pNIPAM-BSA-pGal (*9*)

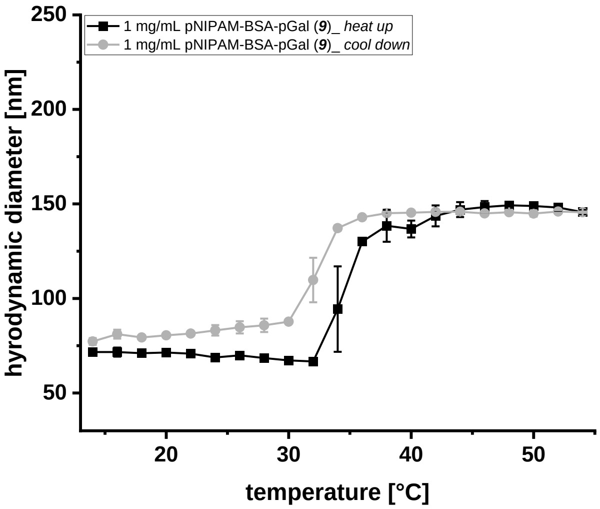

*Fig. S59: Hydrodynamic diameter profile of temperature-dependent particle formation and dissociation of the pNIPAM-BSA-pGal conjugates **9** at a concentration of 1 mg/mL in PBS buffer with a heating rate of 1 °C/min.*

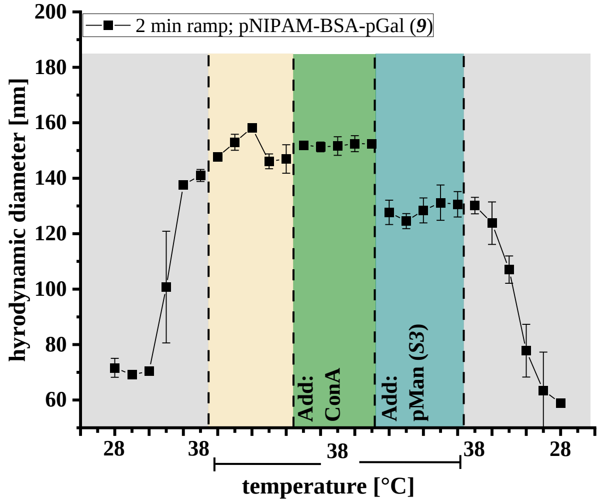

*Fig. S60: Hydrodynamic diameter profile of temperature-dependent particle formation and dissociation with the polymeric inhibitor pMan (**S3**) (15 mM in LBB) of the pNIPAM-BSA-pGal conjugates **9** at a concentration of 13.8 µM in LBB buffer with a heating rate of 1 °C/min.*

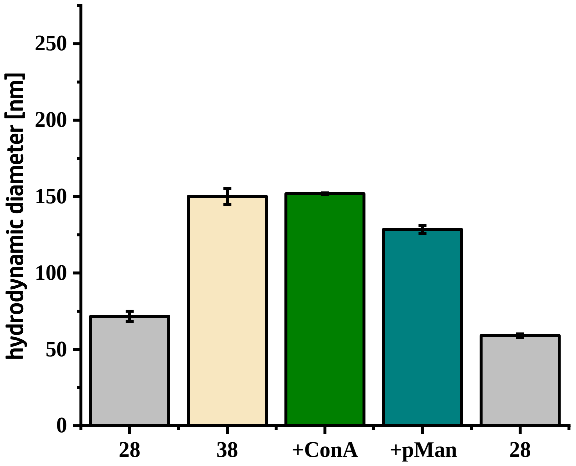

*Fig. S61: The mean values of the hydrodynamic diameter of temperature-dependent particle formation and dissociation from pNIPAM-BSA-pGal conjugates **9** at a concentration of 13.8 µM in LBB buffer with a heating rate of 1 °C/min and with the polymeric inhibitor pMan (**S3**) (15 mM in LBB).*

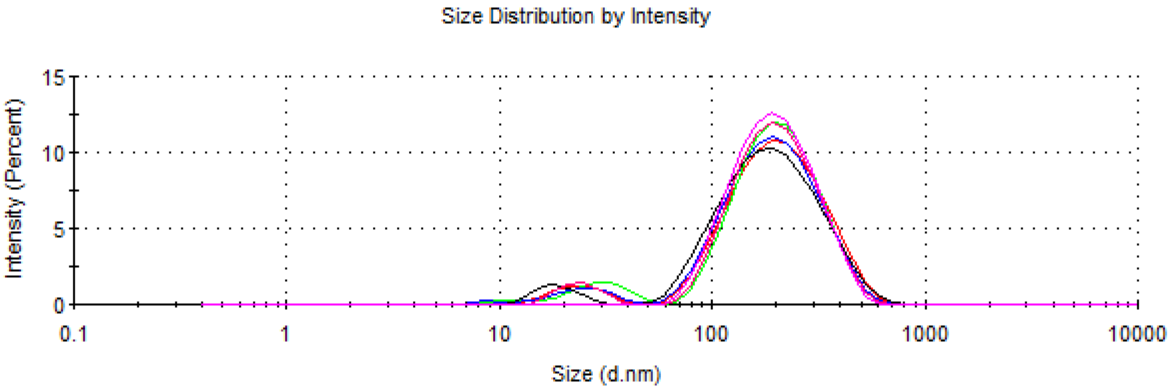

*Fig. S62: Hydrodynamic diameter distribution of pNIPAM-BSA-pGal conjugates **9** at a concentration of 13.8 µM in LBB buffer with a heating rate of 1 °C/min after particle formation (38°C).*

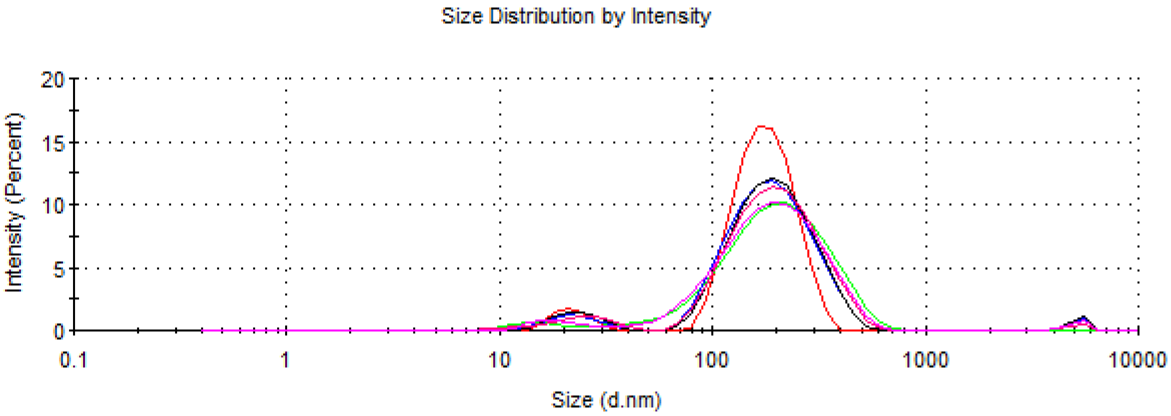

*Fig. S63: Hydrodynamic diameter distribution of pNIPAM-BSA-pGal conjugates **9** at a concentration of 13.8 µM in LBB buffer with a heating rate of 1 °C/min with lectin (ConA) corona at 38°C.*

Lectin stabilized particle below LCST followed by inhibition

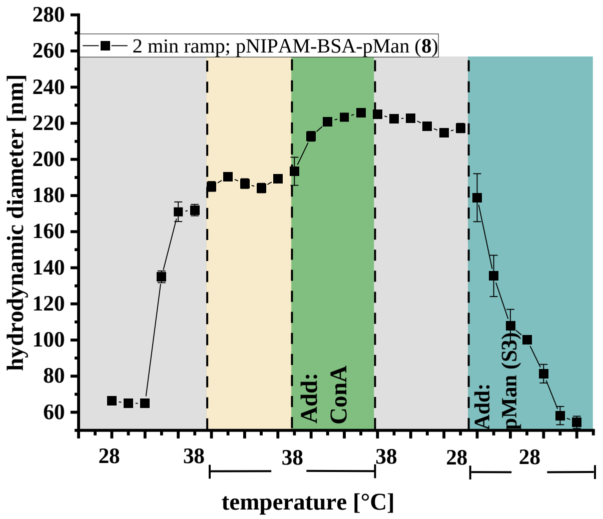

*Fig. S64: Hydrodynamic diameter profile of temperature-dependent particle formation and dissociation with the polymeric inhibitor pMan (**S3**) (15 mM in LBB) of the pNIPAM-BSA-pMan conjugates **8** at a concentration of 13.8 µM in LBB buffer with a heating rate of 1 °C/min. The ConA fixed particle is also stable below the LCST.*

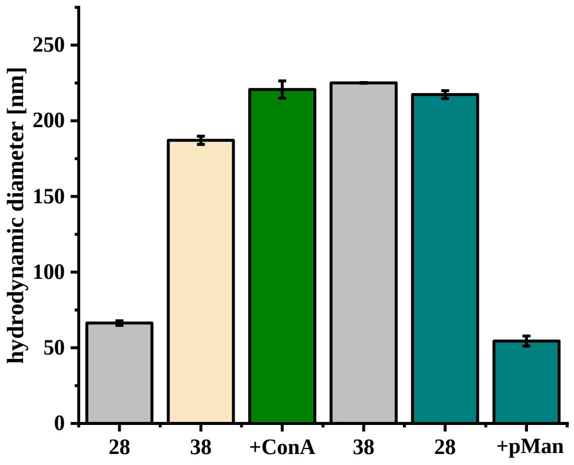

*Fig. S65: The mean values of the hydrodynamic diameter of temperature-dependent particle formation and dissociation from PNIPAM-BSA-pMan conjugates **8** at a concentration of 13.8 µM in LBB buffer with a heating rate of 1 °C/min and with the polymeric inhibitor pMan (**S3**) (15 mM in LBB).*

2.6 DLS inhibition Competition Man mix Gal

50% pNIPAM-BSA-pMan (**8**)/ 50% of pNIPAM-BSA-pGal (**9**)

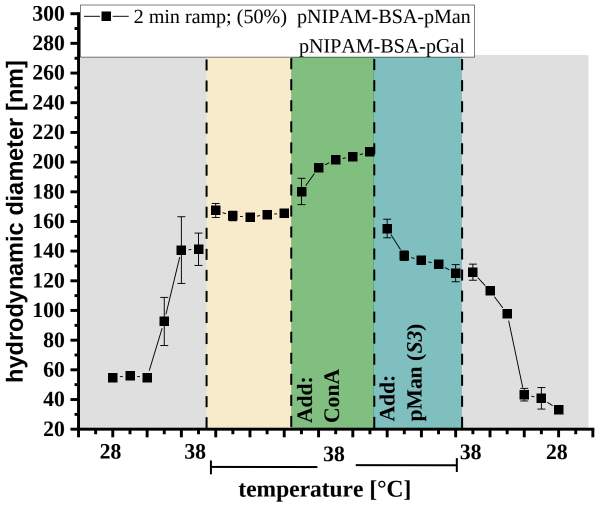

*Fig. S66: Hydrodynamic diameter profile of temperature-dependent particle formation and dissociation with the polymeric inhibitor pMan (**S3**) (15 mM in LBB) of a mixture of 50% pNIPAM-BSA-pMan (**8**) and the other50% of pNIPAM-BSA-pGal (**9**) conjugates at a concentration of 13.8 µM in LBB buffer each with a heating rate of 1 °C/min.*

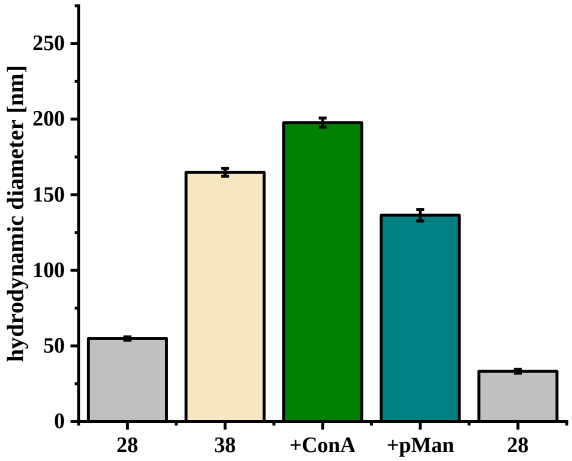

*Fig. S67: The mean values of the hydrodynamic diameter of temperature-dependent particle formation and dissociation from the mixture of 50% pNIPAM-BSA-pMan (**8**) and the other 50% of pNIPAM-BSA-pGal (**9**) at a concentration of 13.8 µM in LBB buffer with a heating rate of 1 °C/min and with the polymeric inhibitor pMan (**S3**) (15 mM in LBB).*

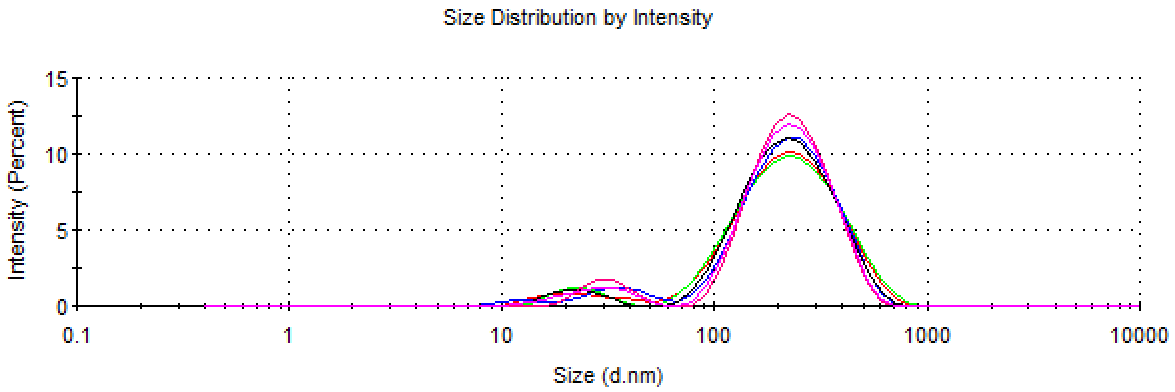

*Fig. S68: Hydrodynamic diameter distribution from the mixture of 50% pNIPAM-BSA-pMan (**8**) and the other 50% of pNIPAM-BSA-pGal (**9**) at a concentration of 13.8 µM in LBB buffer with a heating rate of 1 °C/min after particle formation (38°C).*

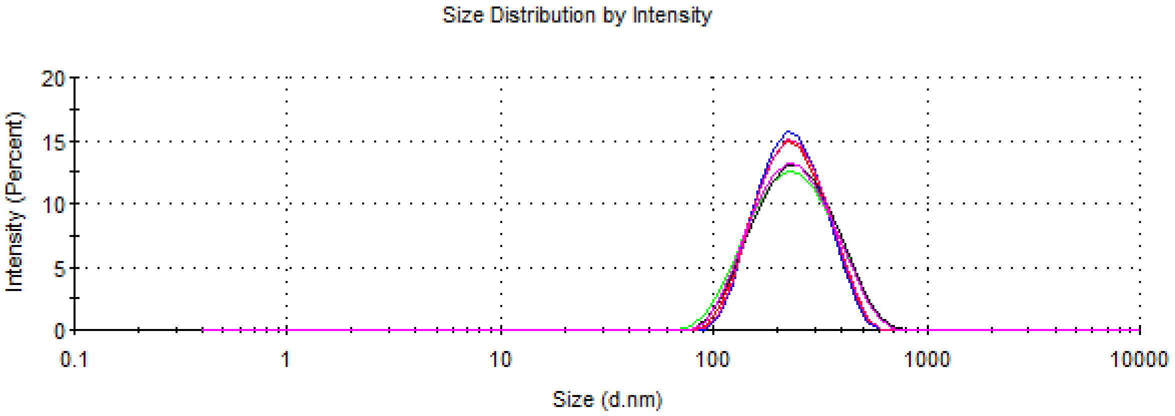

*Fig. S69: Hydrodynamic diameter distribution from the mixture of 50% pNIPAM-BSA-pMan (**8**) and the other 50% of pNIPAM-BSA-pGal (**9**) at a concentration of 13.8 µM in LBB buffer with a heating rate of 1 °C/min with lectin (ConA) corona at 38°C.*

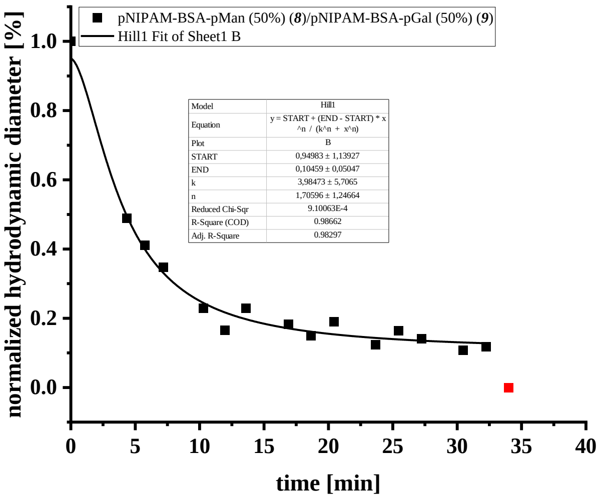

*Fig. S70: Kinetics of 50% pNIPAM-BSA-pMan (**8**) inhibition after pMan addition (Hill 1 Fit).*

30% pNIPAM-BSA-pMan (**8**)/ 70% pNIPAM-BSA-pGal (**9**)

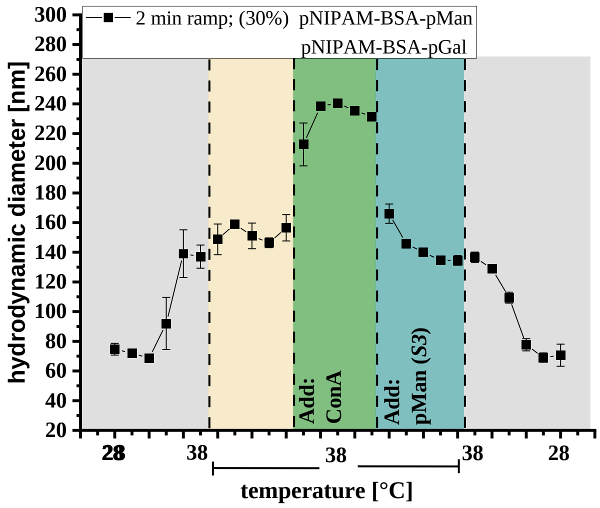

*Fig. S71: Hydrodynamic diameter profile of temperature-dependent particle formation and dissociation with the polymeric inhibitor pMan (**S3**) (15 mM in LBB) of a mixture of 30% pNIPAM-BSA-pMan (**8**) and the other70% of pNIPAM-BSA-pGal (**9**) conjugates at a concentration of 13.8 µM in LBB buffer each with a heating rate of 1 °C/min.*

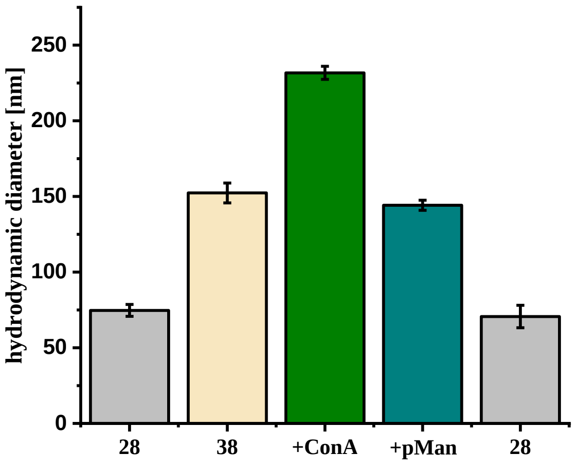

*Fig. S72: The mean values of the hydrodynamic diameter of temperature-dependent particle formation and dissociation from the mixture of 30% pNIPAM-BSA-pMan (**8**) and the other 70% of pNIPAM-BSA-pGal (**9**) at a concentration of 13.8 µM in LBB buffer with a heating rate of 1 °C/min and with the polymeric inhibitor pMan (**S3**) (15 mM in LBB).*

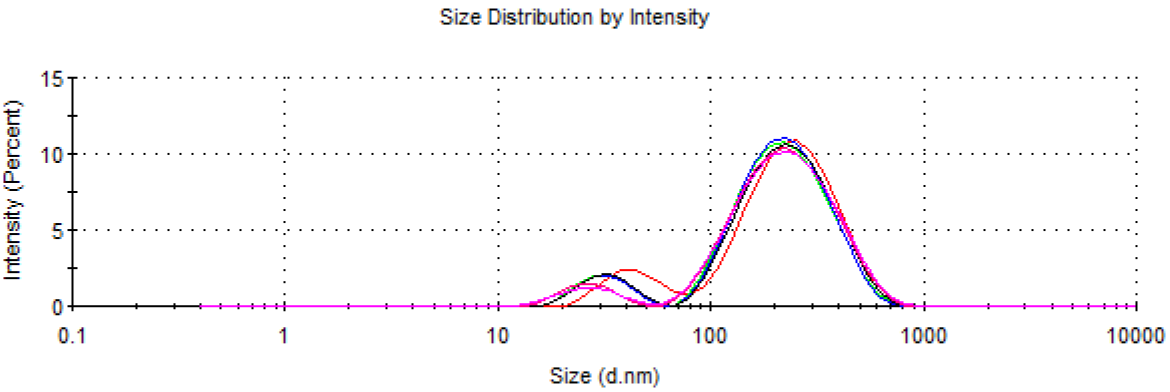

*Fig. S73: Hydrodynamic diameter distribution from the mixture of 30% pNIPAM-BSA-pMan (**8**) and the other 70% of pNIPAM-BSA-pGal (**9**) at a concentration of 13.8 µM in LBB buffer with a heating rate of 1 °C/min after particle formation (38°C).*

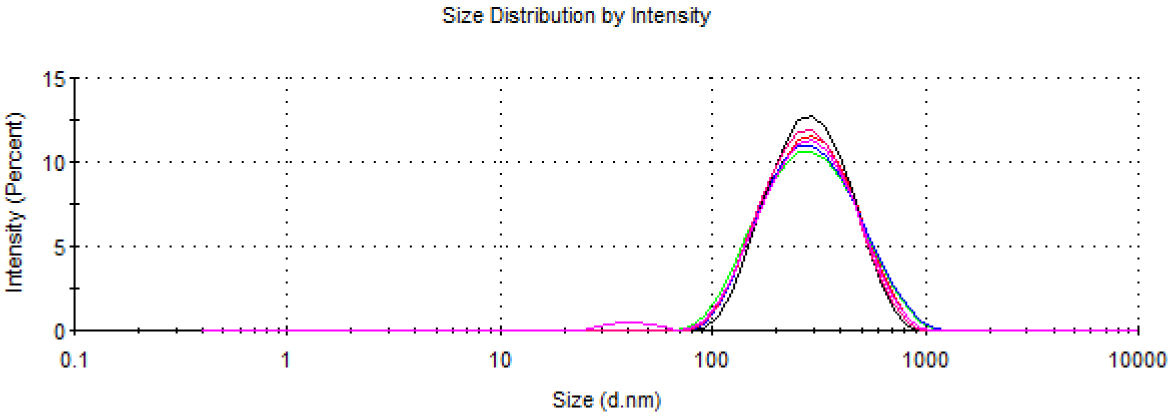

*Fig. S74: Hydrodynamic diameter distribution from the mixture of 30% pNIPAM-BSA-pMan (**8**) and the other 70% of pNIPAM-BSA-pGal (**9**) at a concentration of 13.8 µM in LBB buffer with a heating rate of 1 °C/min with lectin (ConA) corona at 38°C.*

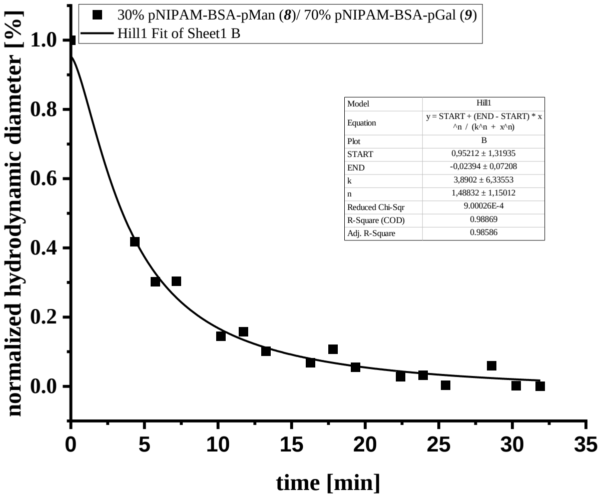

*Fig. S75: Kinetics of 30% pNIPAM-BSA-pMan (**8**) inhibition after pMan addition (Hill 1 Fit).*

20% pNIPAM-BSA-pMan (**8**)/ 80% pNIPAM-BSA-pGal (**9**)

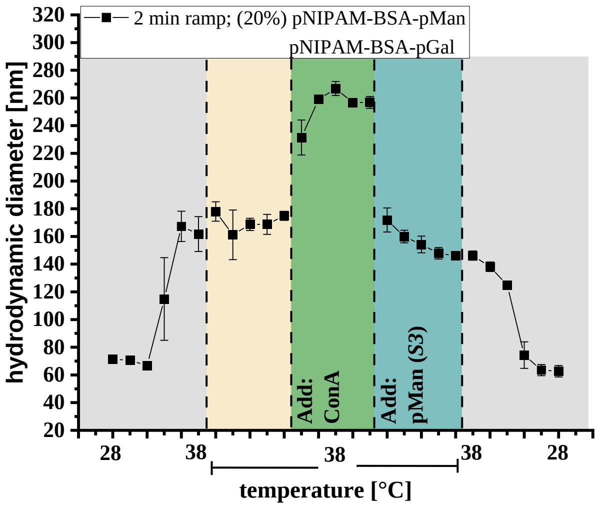

*Fig. S76: Hydrodynamic diameter profile of temperature-dependent particle formation and dissociation with the polymeric inhibitor pMan (**S3**) (15 mM in LBB) of a mixture of 20% pNIPAM-BSA-pMan (**8**) and the other 80% of pNIPAM-BSA-pGal (**9**) conjugates at a concentration of 13.8 µM in LBB buffer each with a heating rate of 1 °C/min.*

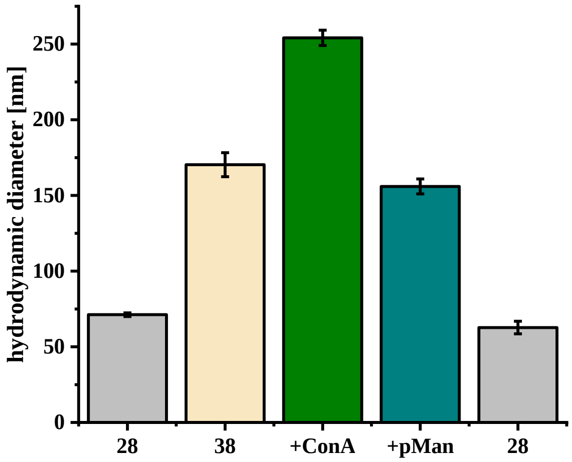

*Fig. S77: The mean values of the hydrodynamic diameter of temperature-dependent particle formation and dissociation from the mixture of 20% pNIPAM-BSA-pMan (**8**) and the other 80% of pNIPAM-BSA-pGal (**9**) at a concentration of 13.8 µM in LBB buffer with a heating rate of 1 °C/min and with the polymeric inhibitor pMan (**S3**) (15 mM in LBB).*

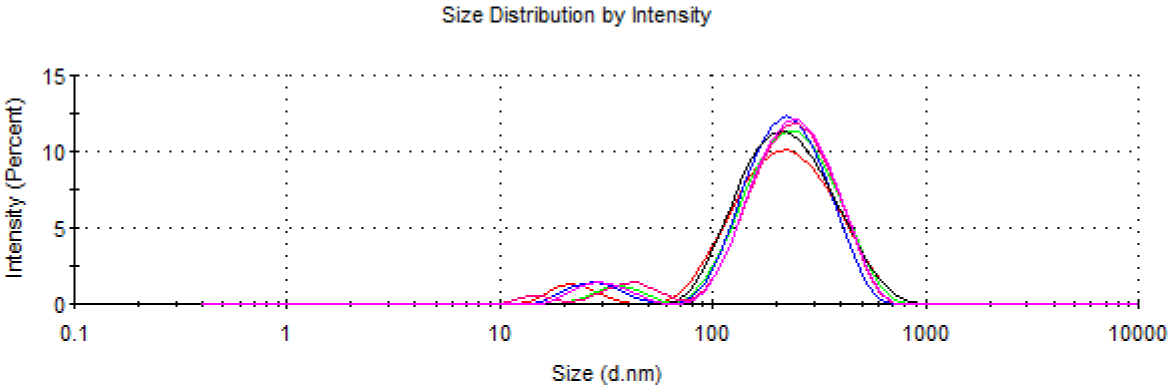

*Fig. S78: Hydrodynamic diameter distribution from the mixture of 20% pNIPAM-BSA-pMan (**8**) and the other 80% of pNIPAM-BSA-pGal (**9**) at a concentration of 13.8 µM in LBB buffer with a heating rate of 1 °C/min after particle formation (38°C).*

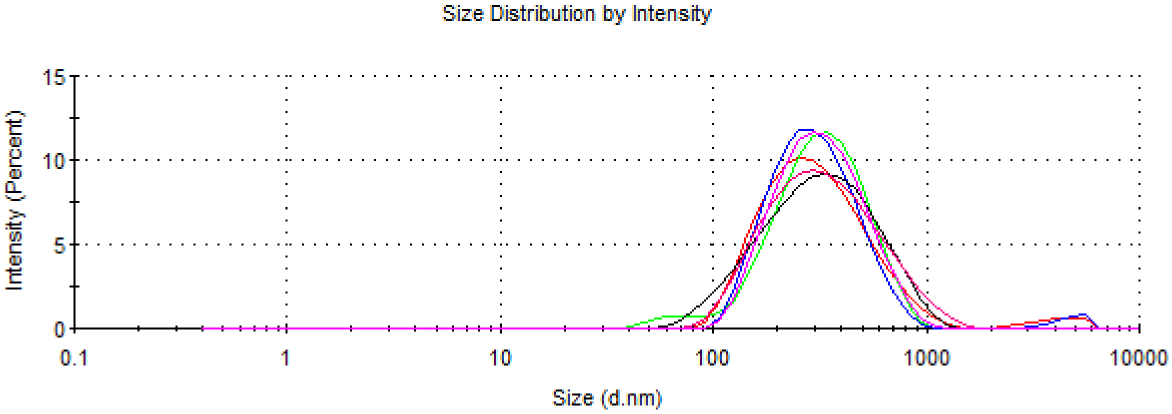

*Fig. S79: Hydrodynamic diameter distribution from the mixture of 30% pNIPAM-BSA-pMan (**8**) and the other 70% of pNIPAM-BSA-pGal (**9**) at a concentration of 13.8 µM in LBB buffer with a heating rate of 1 °C/min with lectin (ConA) corona at 38°C.*

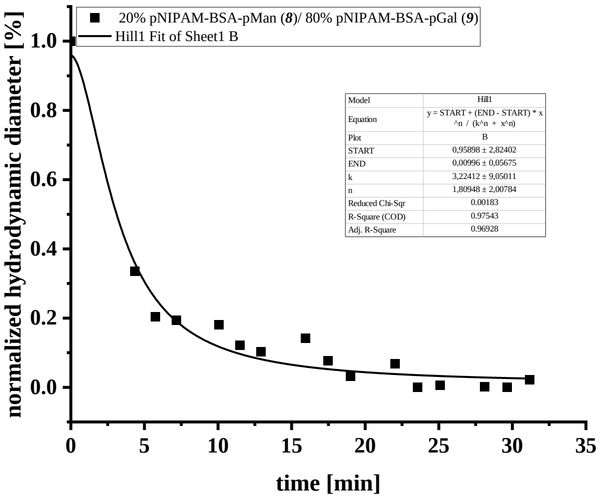

*Fig. S80: Kinetics of 20% pNIPAM-BSA-pMan (**8**) inhibition after pMan addition (Hill 1 Fit).*

11% pNIPAM-BSA-pMan (**8**)/ 89% pNIPAM-BSA-pGal (**9**)

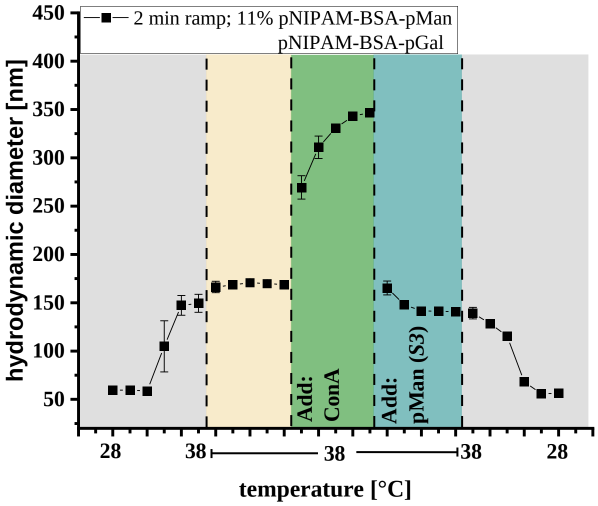

*Fig. S81: Hydrodynamic diameter profile of temperature-dependent particle formation and dissociation with the polymeric inhibitor pMan (**S3**) (15 mM in LBB) of a mixture of 11% pNIPAM-BSA-pMan (**8**) and the other 89% of pNIPAM-BSA-pGal (**9**) conjugates at a concentration of 13.8 µM in LBB buffer each with a heating rate of 1 °C/min.*

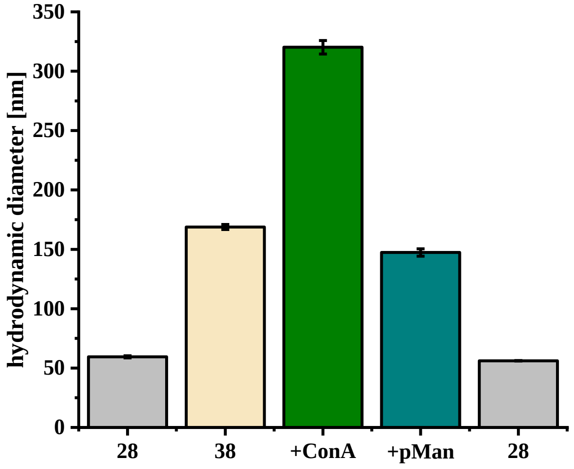

*Fig. S82: The mean values of the hydrodynamic diameter of temperature-dependent particle formation and dissociation from the mixture of 11% pNIPAM-BSA-pMan (**8**) and the other 89% of pNIPAM-BSA-pGal (**9**) at a concentration of 13.8 µM in LBB buffer with a heating rate of 1 °C/min and with the polymeric inhibitor pMan (**S3**) (15 mM in LBB).*

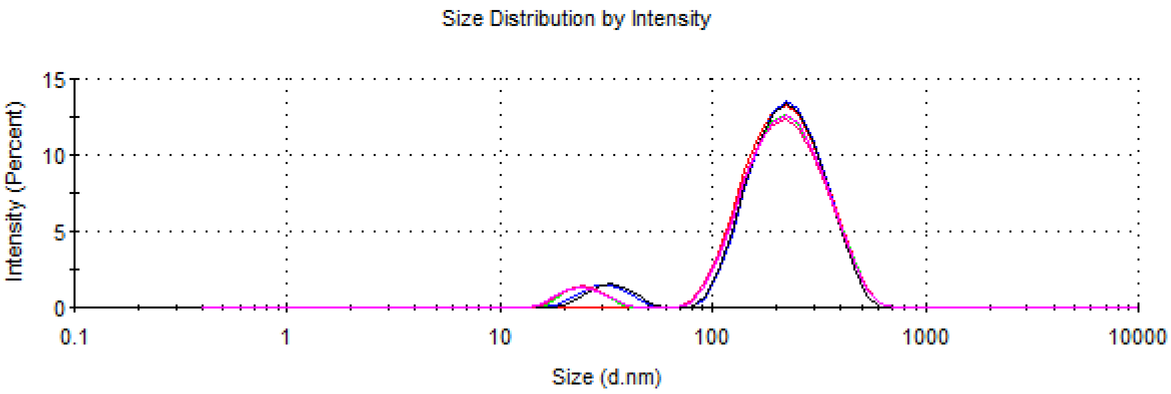

*Fig. S83: Hydrodynamic diameter distribution from the mixture of 11% pNIPAM-BSA-pMan (**8**) and the other 89% of pNIPAM-BSA-pGal (**9**) at a concentration of 13.8 µM in LBB buffer with a heating rate of 1 °C/min after particle formation (38°C).*

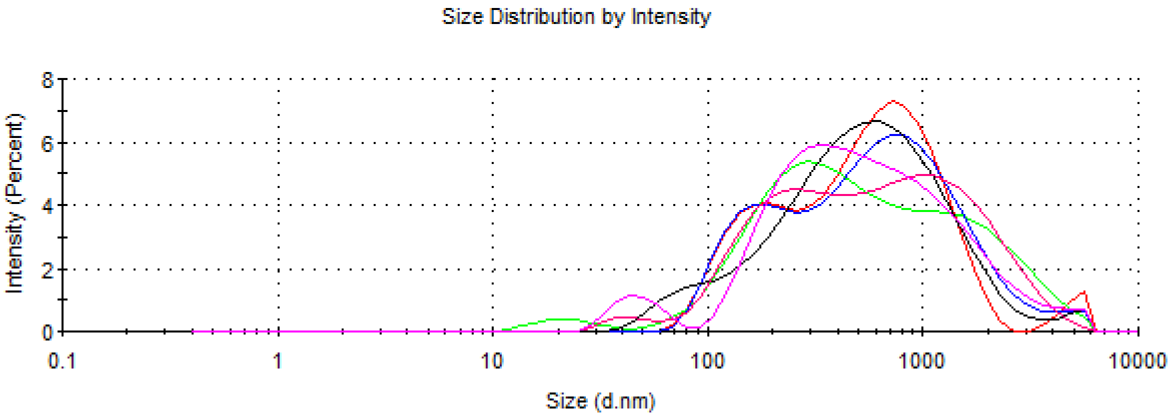

*Fig. S84: Hydrodynamic diameter distribution from the mixture of 11% pNIPAM-BSA-pMan (**8**) and the other 89% of pNIPAM-BSA-pGal (**9**) at a concentration of 13.8 µM in LBB buffer with a heating rate of 1 °C/min with lectin (ConA) corona at 38°C.*

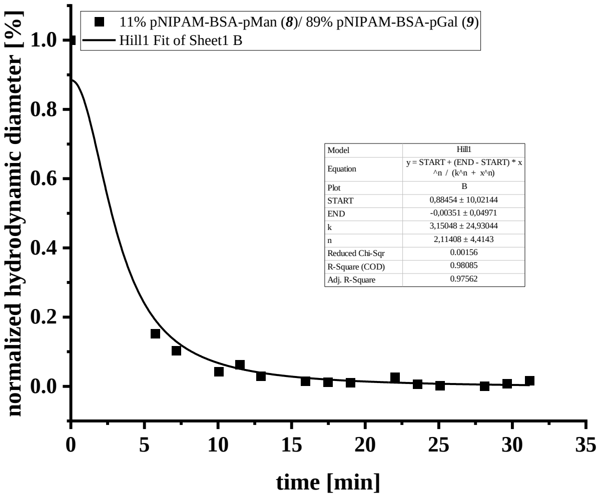

*Fig. S85: Kinetics of 11% pNIPAM-BSA-pMan (**8**) inhibition after pMan addition (Hill 1 Fit).*

6% pNIPAM-BSA-pMan (**8**)/ 94% pNIPAM-BSA-pGal (**9**)

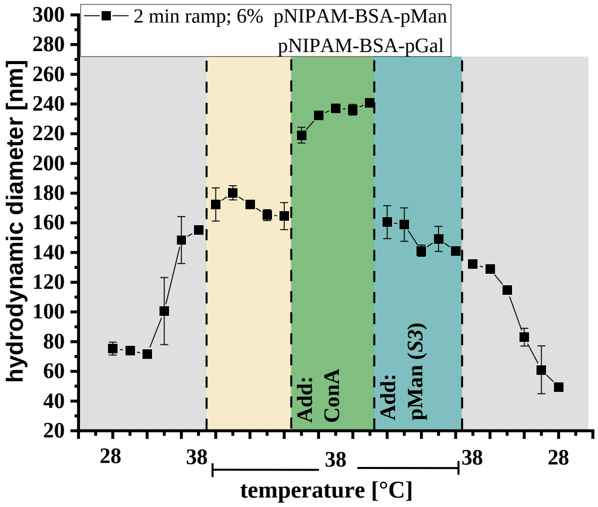

*Fig. S86: Hydrodynamic diameter profile of temperature-dependent particle formation and dissociation with the polymeric inhibitor pMan (**S3**) (15 mM in LBB) of a mixture of 6% pNIPAM-BSA-pMan (**8**) and the other 94% of pNIPAM-BSA-pGal (**9**) conjugates at a concentration of 13.8 µM in LBB buffer each with a heating rate of 1 °C/min.*

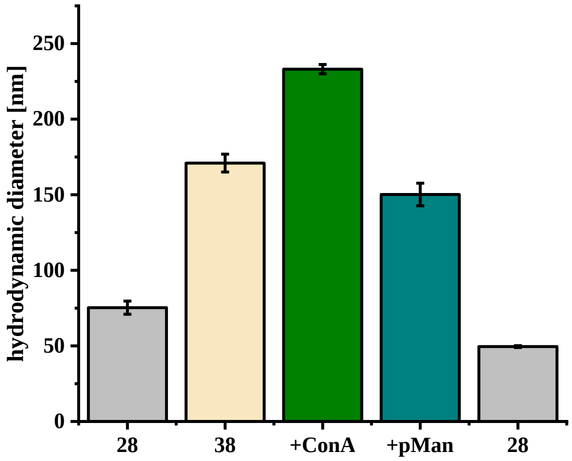

*Fig. S87: The mean values of the hydrodynamic diameter of temperature-dependent particle formation and dissociation from the mixture of 6% pNIPAM-BSA-pMan (**8**) and the other 94% of pNIPAM-BSA-pGal (**9**) at a concentration of 13.8 µM in LBB buffer with a heating rate of 1 °C/min and with the polymeric inhibitor pMan (**S3**) (15 mM in LBB).*

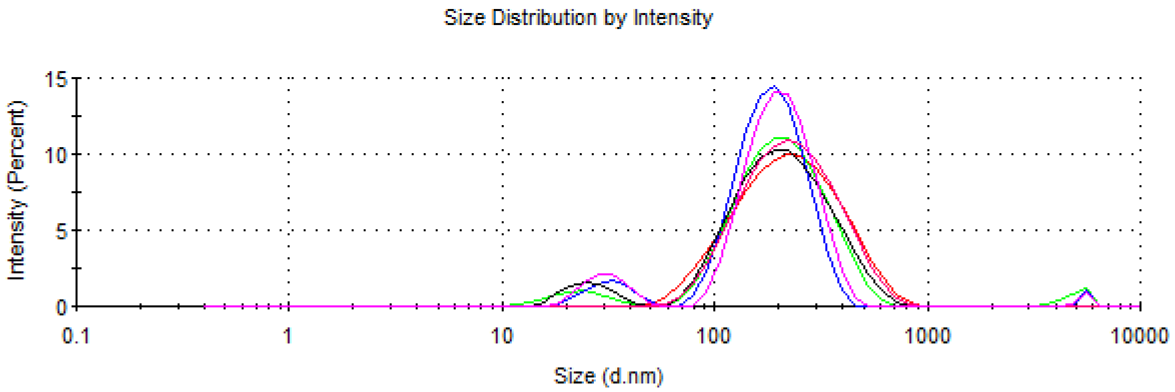

*Fig. S88: Hydrodynamic diameter distribution from the mixture of 6% pNIPAM-BSA-pMan (**8**) and the other 94% of pNIPAM-BSA-pGal (**9**) at a concentration of 13.8 µM in LBB buffer with a heating rate of 1 °C/min after particle formation (38°C).*

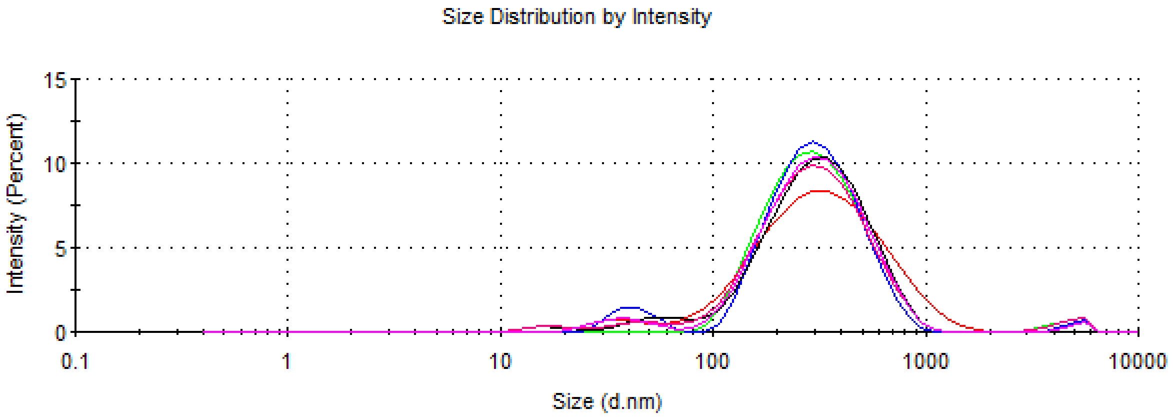

*Fig. S89: Hydrodynamic diameter distribution from the mixture of 6% pNIPAM-BSA-pMan (**8**) and the other 94% of pNIPAM-BSA-pGal (**9**) at a concentration of 13.8 µM in LBB buffer with a heating rate of 1 °C/min with lectin (ConA) corona at 38°C.*

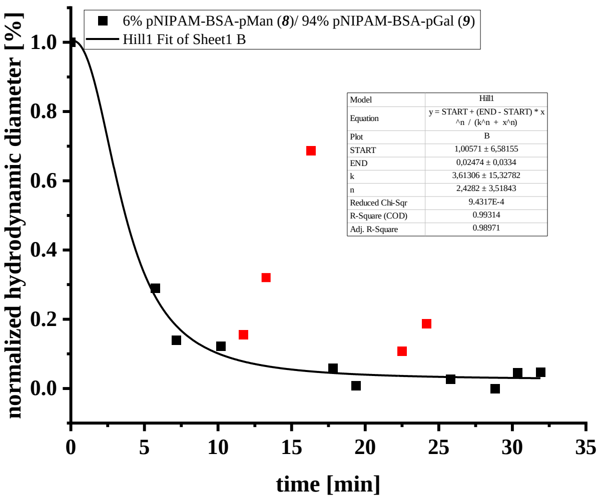

*Fig. S90: Kinetics of 6% pNIPAM-BSA-pMan (**8**) inhibition after pMan addition (Hill 1 Fit).*

3% pNIPAM-BSA-pMan (**8**)/ 97% pNIPAM-BSA-pGal (**9**)

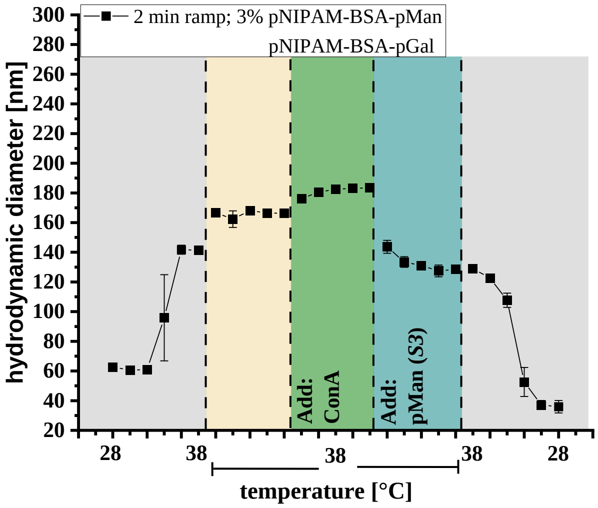

*Fig. S91: Hydrodynamic diameter profile of temperature-dependent particle formation and dissociation with the polymeric inhibitor pMan (**S3**) (15 mM in LBB) of a mixture of 3% pNIPAM-BSA-pMan (**8**) and the other 97% of pNIPAM-BSA-pGal (**9**) conjugates at a concentration of 13.8 µM in LBB buffer each with a heating rate of 1 °C/min.*

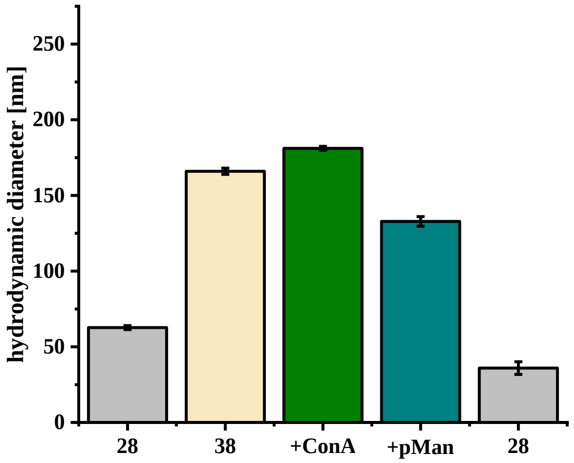

*Fig. S92: The mean values of the hydrodynamic diameter of temperature-dependent particle formation and dissociation from the mixture of 3% pNIPAM-BSA-pMan (**8**) and the other 97% of pNIPAM-BSA-pGal (**9**) at a concentration of 13.8 µM in LBB buffer with a heating rate of 1 °C/min and with the polymeric inhibitor pMan (**S3**) (15 mM in LBB).*

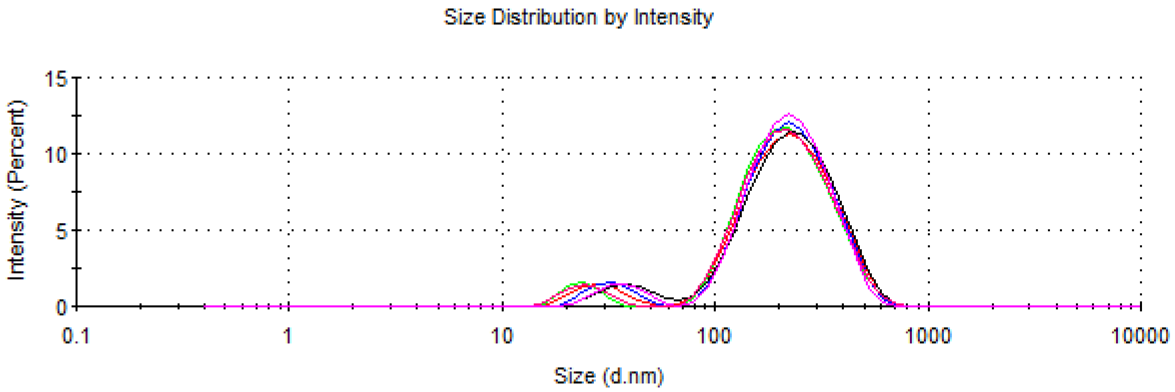

*Fig. S93: Hydrodynamic diameter distribution from the mixture of 3% pNIPAM-BSA-pMan (**8**) and the other 97% of pNIPAM-BSA-pGal (**9**) at a concentration of 13.8 µM in LBB buffer with a heating rate of 1 °C/min after particle formation (38°C).*

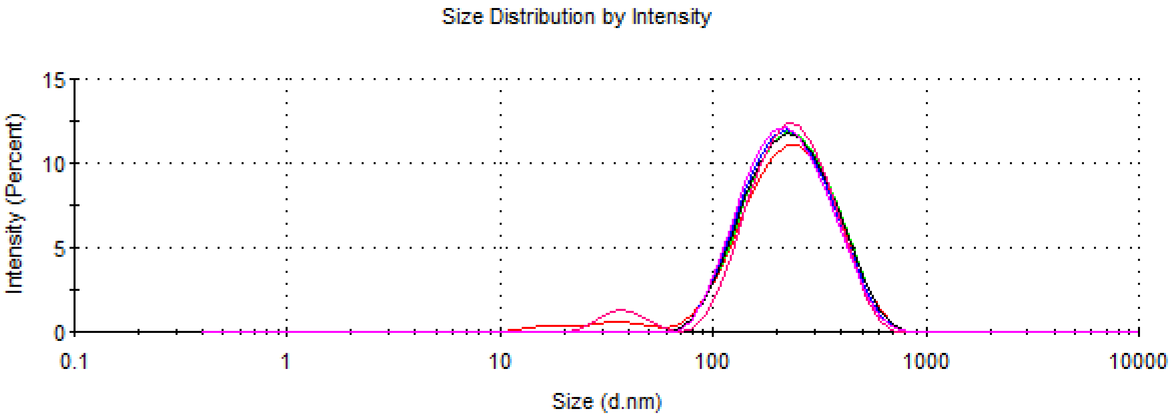

*Fig. S94: Hydrodynamic diameter distribution from the mixture of 3% pNIPAM-BSA-pMan (**8**) and the other 97% of pNIPAM-BSA-pGal (**9**) at a concentration of 13.8 µM in LBB buffer with a heating rate of 1 °C/min with lectin (ConA) corona at 38°C.*

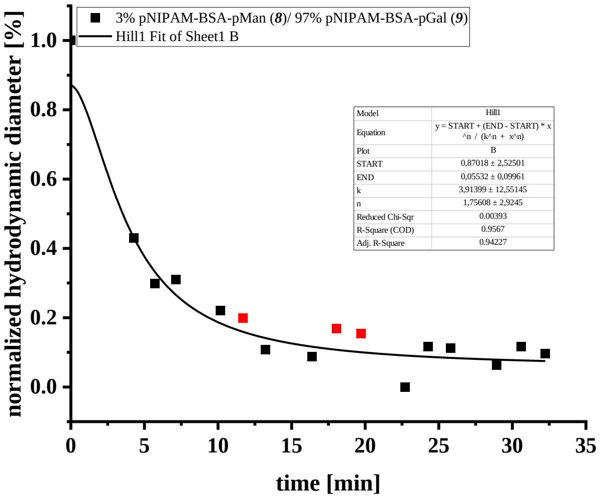

*Fig. S95: Kinetics of 3% pNIPAM-BSA-pMan (**8**) inhibition after pMan addition (Hill 1 Fit).*

pNIPAM-BSA-pMan (**8**) long-term experiment

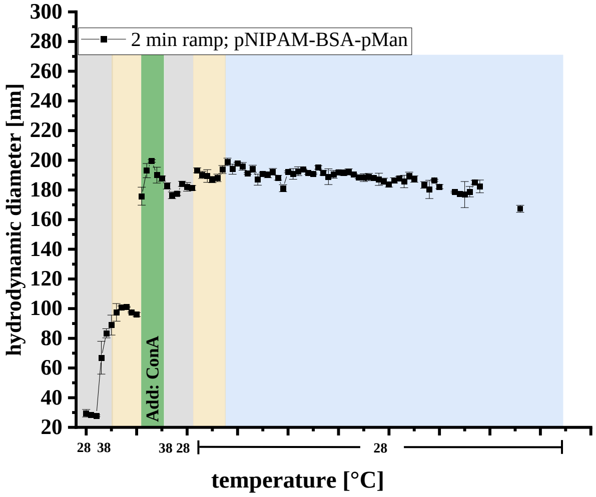

*Fig. S96: Hydrodynamic diameter profile of temperature-dependent particle formation and dissociation with the polymeric inhibitor pMan (**S3**) (15 mM in LBB) of pNIPAM-BSA-pMan (**8**) conjugate at a concentration of 13.8 µM in LBB buffer each with a heating rate of 1 °C/min. The last measurement point took place after approx. 4 days.*

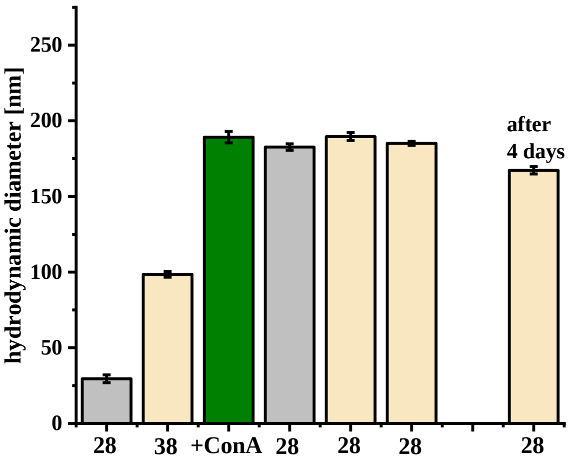

*Fig. S97: The mean values of the hydrodynamic diameter of temperature-dependent particle formation and dissociation from pNIPAM-BSA-pMan conjugates **8** at a concentration of 13.8 µM in LBB buffer with a heating rate of 1 °C/min and with the polymeric inhibitor pMan (**S3**) (15 mM in LBB). The last measurement point took place after approx. 4 days.*

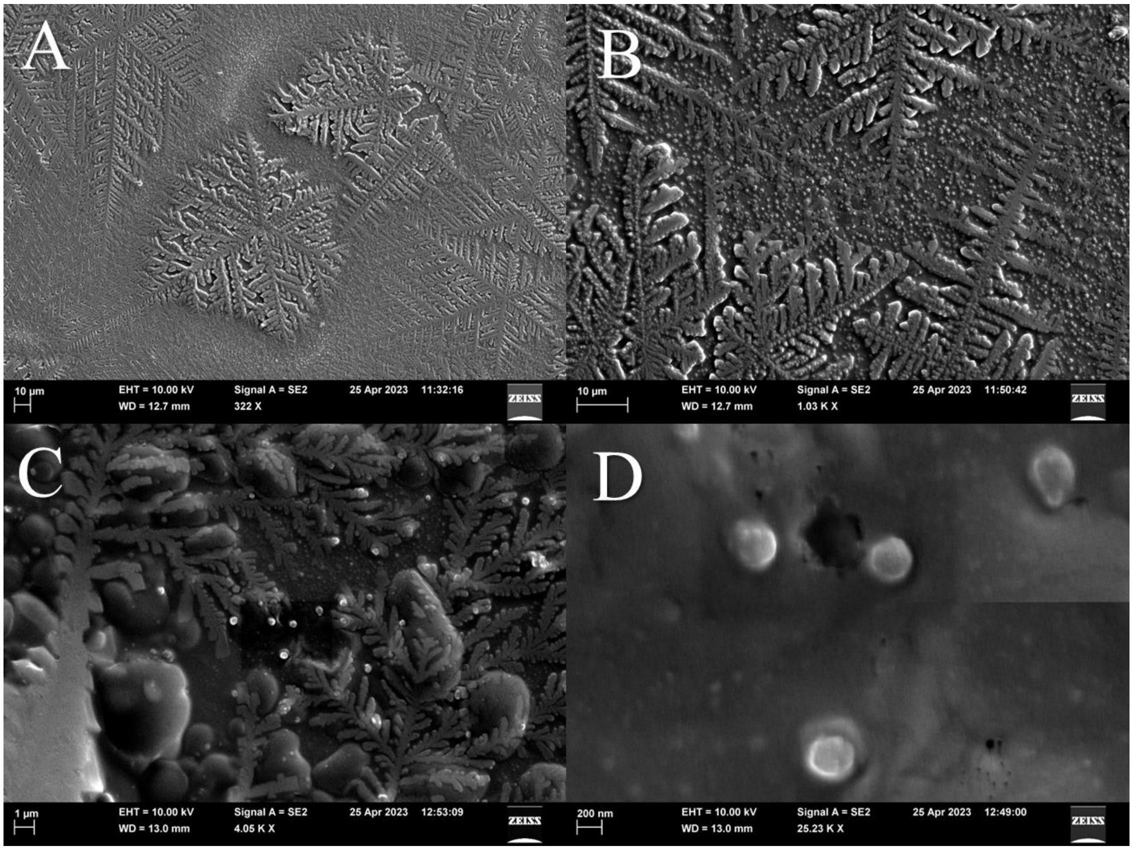

*Fig. S98: SEM images of pNIPAM-BSA-pMan conjugate (**8**) fixed via ConA as nanoparticles in LBB below the LCST. **A** and **B** show concentrations at 4.6µM and **C** and **D** at 0.46µM.*

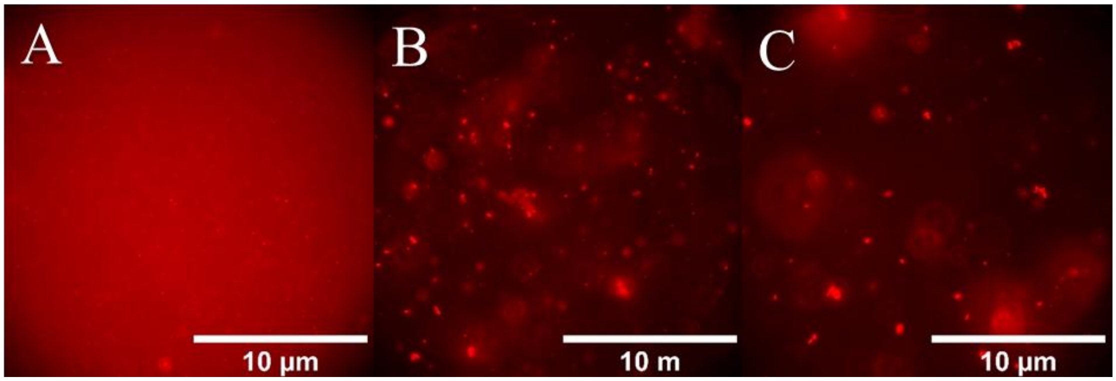

*Fig. S99: Microscopic images of the pNIPAM-BSA-pMan conjugate (**8**) below and above the LCST with the addition of the lectine ConA$^{Alexa\ 647}$: **A**: Fluorescence image (excitation: 641 nm, emission: 690 nm) of conjugate **8** after addition of ConA below the LCST (28°C) - entire solution fluoresces and no local enrichments, **B**: Fluorescence image (excitation: 641 nm, emission: 690 nm) of conjugate **8** after addition of ConA above the LCST (38°C) - local concentration of ConA at formed particles, **C**: Fluorescence image (excitation: 641 nm, emission: 690 nm) of conjugate **8** after addition of ConA above the LCST (10 min, 38°C) - formation of the nanoparticles with binding of the lectin to the mannose-presenting micelles.*

2.7 Synthesis of inhibitor polymers S3 and S4

Synthesis of pMan (S3) and pGal (S4)

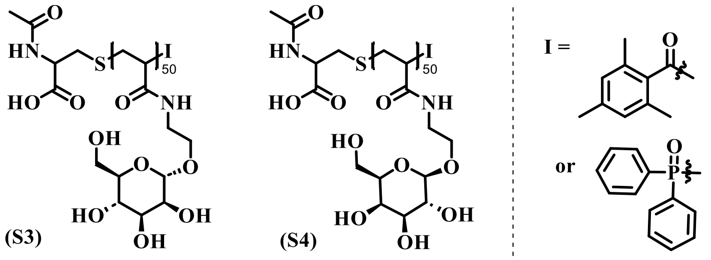

The syntheses of structures **S3** and **S4** have already been published by Feldhof et.al.[2]

References

[1] K. L. Heredia, D. Bontempo, T. Ly, J. T. Byers, S. Halstenberg, H. D. Maynard, *J. Am. Chem. Soc.* **2005**, *127*, 16955-16960.

[2] M. Feldhof, S. Sperzel, L. Bonda, S. Boye, A. Braunschweig, U. I. Gerling-Driessen, L. Hartmann, *Chem. Sci.* **2024**, *15*, 16768-16777.
